# Synthesis, structural analysis and reactivity of alkynyl sulfide anions

**DOI:** 10.1039/d6qi01030j

**Published:** 2026-07-10

**Authors:** Sunita Mondal, Quentin Le Dé, Daniel A. Santos Oliveira, Daniela Rodrigues Silva, Angela De Feudis, F. Matthias Bickelhaupt, Viktoria H. Gessner

**Affiliations:** a Faculty of Chemistry and Biochemistry, Ruhr-University Bochum Universitätsstrasse 150 44801 Bochum Germany viktoria.gessner@rub.de; b Department of Chemistry and Pharmaceutical Sciences, Vrije Universiteit Amsterdam De Boelelaan 1108 1081 HZ Amsterdam The Netherlands f.m.bickelhaupt@vu.nl; c Department of Fundamental Chemistry, Institute of Chemistry, University of São Paulo Av. Prof. Lineu Prestes 748 São Paulo 055508-000 Brazil; d Institute of Molecules and Materials, Radboud University Nijmegen Heyendaalseweg 135 6525 AJ Nijmegen The Netherlands; e Department of Chemical Sciences, University of Johannesburg Auckland Park Johannesburg 2006 South Africa

## Abstract

Ketenyl anions are versatile, isolable reagents that facilitate the formation of ketenes, carbonyl-containing compounds, and functionalized heterocycles. In contrast, their thio analogues have only been prepared *in situ* and their alkali metal complexes remain elusive. Here, we report the facile synthesis and isolation of a series of thioketenyl anions through CS-for-CO exchange, using carbon disulfide or thioisocyanates as CS transfer reagents. Spectroscopic and crystallographic studies, combined with quantum chemical calculations, reveal that the electronic structure of these anions is best described by the alkynyl sulfide form with a C–C triple bond, with a minor contribution from the thioketenyl structure. This differs from previously reported related anions and can be attributed to the enhanced π-accepting properties of the carbon monosulfide ligand, which can be rationalized by the Dewar–Chatt–Duncanson model. This insight bridges the bonding in these carbanionic species with that in transition metal complexes. The unique bonding in alkynyl sulfides is reflected in their reactivity patterns, opening pathways to sulfur-heterocycles and neutral alkynyl sulfides *via* S-centered reactivity.

## Abstract 2

Structure elucidation, reactivity studies and detailed bonding analysis of alkali metal alkynyl sulfides are reported.

## Graphical abstract

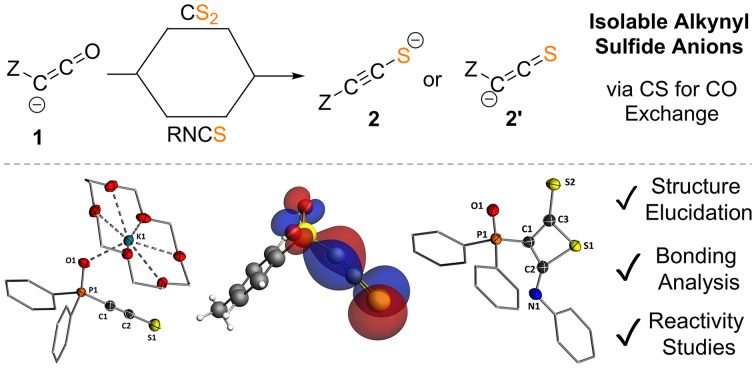

## Introduction

**Fig. 1:**
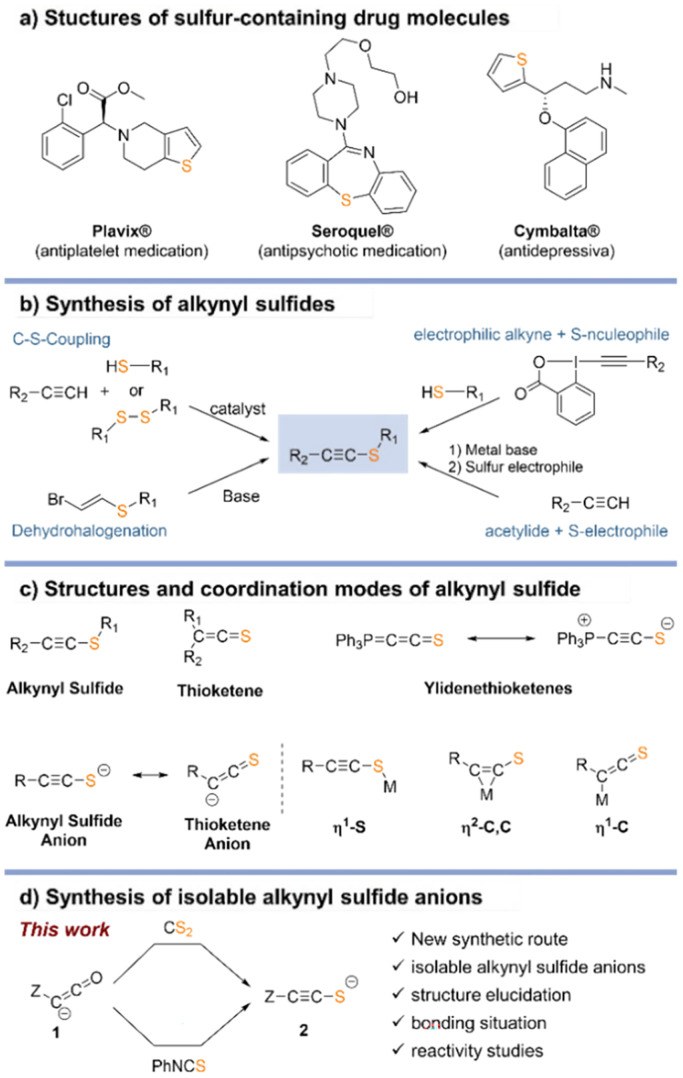
(a) Examples of sulfur-containing drug molecules, (b) structure and synthesis of alkynyl sulfides, (c) canonical structures of anionic alkynyl sulfides and coordination modes with transition metals and (d) synthesis of free alkynyl sulfide anions reported in this work.

Thio compounds, particularly sulfur heterocycles, play a significant role in pharmaceutical^1–3^ and material chemistry,^4,5^ such as for the preparation of sulfur-containing drugs like Plavix, Seroquel or Cymbalta (Fig. 1a). Alkynyl sulfides (thioalkynes) represent versatile sulfur-containing building blocks that provide access to a range of functionalized organosulfur compounds, including sulfur-containing heterocycles.^6–8^ The synthesis of alkynyl sulfides is achieved through various methods,^9^ including the transition metal-catalyzed coupling of terminal alkynes and thiols^10–12^ or the reaction of alkynyl anions with disulfides or other sulfur electrophiles (Fig. 1b).^13,14^ Furthermore, dehydrohalogenation reactions of β-halovinyl thioethers^15^ and the coupling of electrophilic alkyne reagents, such as hypervalent iodine compounds,^16^ have been reported. In contrast to other heteroatom-functionalized alkynes, such as ynamines or alkynyl phosphines, alkynyl sulfides are usually stable reagents. However, in certain cases, they were found to undergo [3,3]-sigmatropic^17,18^ or [1,3]-sigmatropic^19,20^ rearrangements to form thioketene derivatives. The close relationship between alkynyl sufides and thioketenes becomes evident from ylidenethioketenes. Their structure is best described by both, the alkynyl sulfide (R^+^ –C≡C–S^−^) and the thioketene form (R=C=C=S).^21,22^

Anionic alkynyl sulfides, R–C≡C–S^−^, serve as a versatile starting point for the synthesis of alkynyl sulfides and more complex thio compounds (Fig. 1c). They can be prepared by deprotonative cleavage of thiadiazoles^23,24^ and addition of elemental sulfur to lithium acetylides,^25–28^ or can be generated in the coordination sphere of transition metals through oxidative addition^29–32^ or reductive S–C bond cleavage in alkynyl thioethers.^33,34^ Like neutral alkynyl sulfide, the anions can be described by two mesomeric forms: as alkynyl sulfide with a linear geometry and the negative charge localized on the sulfur atom, or as bent thioketenyl anion, with the negative charge residing on the carbon atom. Isolation and structure analysis of various transition metal complexes of alkynyl sulfides revealed a preferred coordination of the anionic alkynyl sulfides either *via* the sulfur atom^25,26^ or the C–C triple bond,^33,34^ suggesting a preference of the alkynyl sulfide form (Fig. 1c). A rearrangement to the thioketenyl form and exclusive coordination to the C1 carbon atom have rarely been discussed.^25^ In contrast, alkali metal alkynyl sulfides have only been utilized as *in situ*-generated reagents and have never been isolated despite their applicability in the synthesis of heterocycles.^35–37^

Previously, we reported the synthesis of the anionic cyano-substituted alkynyl sulfide [NCCCS]^−^ (2^CN^) through the addition of carbon disulfide (CS_2_) to the corresponding ketenyl anion [NCCCO]^−^1^CN^.^38^ Unfortunately, 2^CN^ could not be obtained in crystalline form, preventing its experimental structure elucidation. However, density functional theory (DFT) calculations indicated that 2^CN^ – in contrast to the cyanoketenyl anion – adopts a linear geometry, consistent with its description as alkynyl sulfide anion. This conclusion is in line with earlier computational investigations of the parent H-substituted system.^39^ To date, no other alkali metal alkynyl sulfide has been isolated, thus leaving not only their structural and electronic properties, but also their principal reactivity virtually unexplored. Herein, we close this gap by isolating a series of alkynyl sulfide anions, including the first crystal structure analyses. In contrast to the linear structure of 2^CN^ predicted by quantum-chemical calculations, these anions adopt slightly bent geometries and exhibit diverse reactivities.

## Results and discussion

### Synthesis of alkynyl sulfide anions

**Scheme 1:**
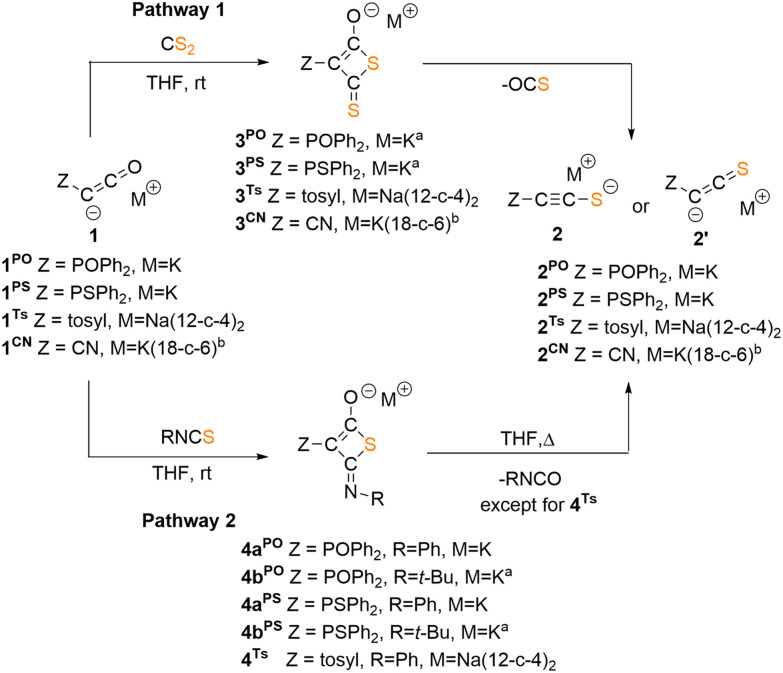
Synthesis of the alkynyl sulfide anions 2^PO^, 2^PS^ and 2^**Ts**^. ^*a*^ Proposed intermediate, not isolated. ^*b*^ Reported in ref. 38.

We began our studies with targeting the phosphinoyl-substituted anions 2^PO^ and 2^PS^. We initially adopted a previously reported synthetic route involving carbon disulfide cycloaddition followed by cycloreversion with carbonyl sulfide (OCS) elimination.^22,40^ Addition of one equivalent of CS_2_ to the potassium salts of ketenyl anions 1^PO^ and 1^PS^ resulted in the formation of a single compound, as evidenced by ^31^P{^1^H} NMR spectroscopy, showing resonances at 2.4 and 12.5 ppm, respectively (Scheme 1).^41,42^ Both compounds could be isolated as yellow solids in excellent 97% yields. X-ray diffraction (XRD) analysis of single crystals of both compounds obtained by addition of one equivalent of 18-crown-6 (18-c-6), confirmed the formation of the targeted alkynyl sulfide anions.

**Fig. 2:**
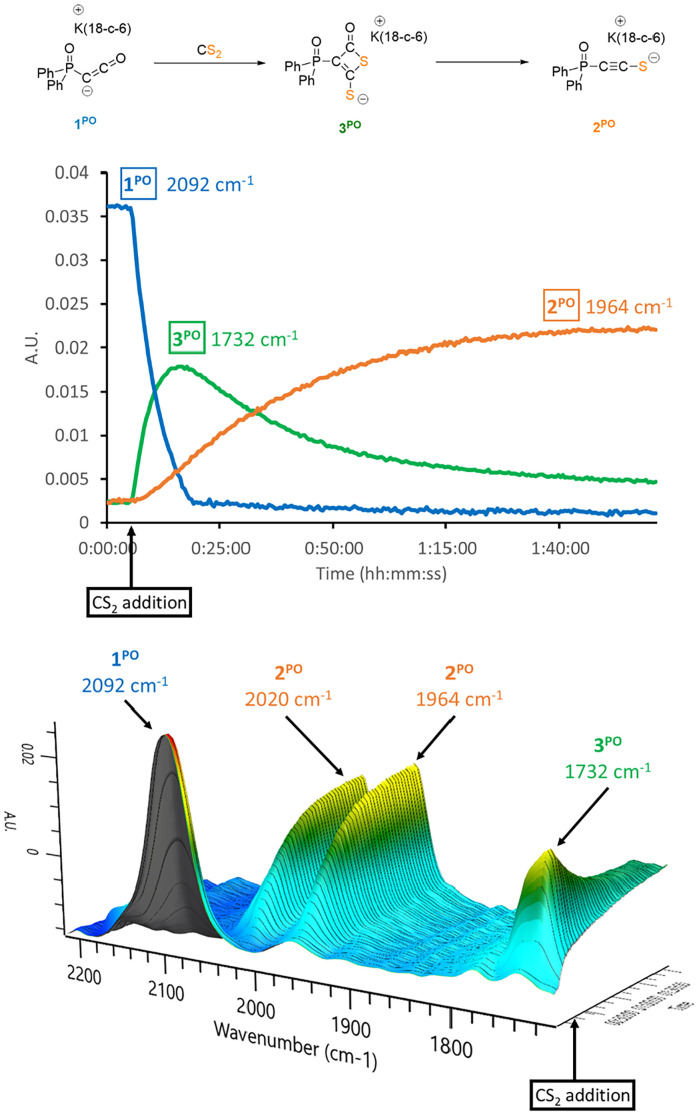
Real-time IR spectroscopic monitoring of the addition of CS_2_ to ketenyl anion 1^PO^ at 0 °C. Before the addition of CS_2_, only 1^PO^ is observed (blue line; *ν*_cco_ = 2092 cm^−1^ [solid state IR data *ν*_cco_ = 2093 cm^−1^]), which subsequently converts to 3^PO^ (green line; *ν*_co_ = 1732 cm^−1^ [gas phase calculate data *ν*_co_ = 1786 cm^−1^]) and finally to 2^PO^ (orange line; *ν*_ccs, sym_ = 1964 cm^−1^ [solid state IR data *ν*_ccs, sym_ = 1950 cm^−1^]).

The facile formation of the potassium salts of 2^PO^ and 2^PS^ contrasts our previous observations with the cyano system 1^CN^. Here, the addition of CS_2_ at room temperature afforded the four-membered ring compound 3^CN^, which only upon heating transformed into the alkynyl sulfide anion 2^CN^. This observation prompted us to investigate whether the formation of 2^PO^ and 2^PS^ also proceeds through the cyclic intermediates 3^PO^ and 3^PS^, respectively. To validate this hypothesis, we monitored the reaction between 1^PO^ and CS_2_ using real-time IR spectroscopy at 0 °C. Upon addition of CS_2_, compound 1^PO^ is rapidly consumed (blue line, Fig. 2), and an intermediate forms, exhibiting an IR band at 1732 cm^−1^ (green line), consistent with the CO stretching frequency calculated for heterocycle 3^PO^. This intermediate subsequently reacts to the final product, exhibiting an IR band at 1964 cm^−1^ (orange line), which matches the spectroscopic features of isolated alkynyl sulfide 2^PO^. Thus, we concluded that the observed intermediate corresponds to heterocycle 3^PO^. Indeed, when the reaction between 1^PS^ and CS_2_ was performed at −40 °C, compound 3^PS^ could be obtained as crystalline solid and its identity was confirmed by XRD analysis (see SI, Fig. S73).

**Fig. 3:**
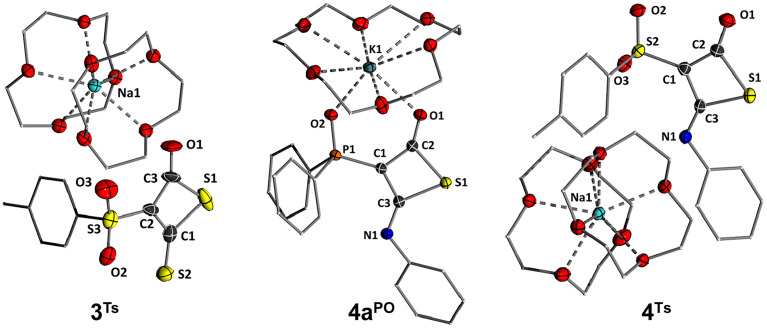
Molecular structures of the crown-ether complexes of 3^Ts^, 4a^PO^ and 4^Ts^ in the solid state. Hydrogen atoms and disordered parts in the molecules are omitted for clarity. Thermal ellipsoids are shown at the 50% probability level.

To further probe the generality of the alkynyl sulfide formation from the corresponding ketenyl anions, we applied the aforementioned strategy to the sodium salt of tosyl ketenyl anion 1^Ts^.^43^ Addition of 1 equivalent of CS_2_ to 1^Ts^ resulted in the formation of an orange compound in an 86% yield, which was identified as intermediate 3^Ts^ based on NMR and XRD analyses (Fig. 3). In contrast to the phosphinoyl-substituted anions, compound 3^Ts^ proved stable towards COS release at room temperature. Even heating a solution of 3^Ts^ in deuterated THF or deuterated 1,2-difluorobenzene for two days in a sealed Young NMR tube at reflux, did not yield the desired alkynyl sulfide. In contrast, heating a THF solution of 3^Ts^ at reflux under static vacuum in a Schlenk tube for two hours, led to the complete conversion to 2^Ts^, as confirmed by NMR spectroscopy and XRD analysis (Fig. 5).

**Fig. 4:**
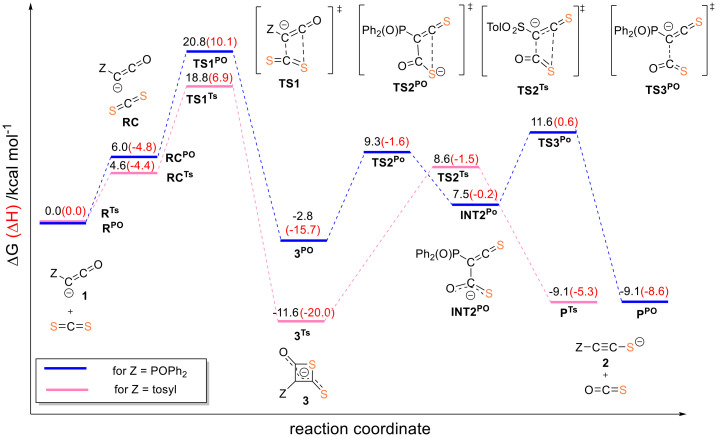
Reaction pathway calculated at the ZORA-BP86/TZ2P level of theory for the formation of 2^PO^ (blue line) and 2^Ts^ (pink line).

To understand the influence of the Z substituent on the reaction conditions required for the synthesis of 2^PO^ and 2^Ts^, we performed DFT calculations at the ZORA^44–46^-BP86^47–49^/TZ2P^50^ level of theory to elucidate the underlying reaction pathways (Fig. 4). The reaction starts with a [2 + 2] cycloaddition affording the four-membered ring intermediate 3. This step is thermodynamically favorable (Δ*G* = −2.8 kcal mol^−1^ for 2^PO^, −11.6 kcal mol^−1^ for 2^Ts^) with activation barriers of 20.8 and 18.8 kcal mol^−1^, consistent with both reactions occurring at ambient temperature. Subsequent OCS extrusion from 3 furnishes the corresponding alkynyl sulfide 2. While for the tosyl system OCS release occurs *via* a concerted [2 + 2] cycloreversion process and is calculated to be endergonic with respect to 3^Ts^ (ΔΔ*G* = +2.5 kcal mol^−1^), the phosphinoyl-substituted system undergoes a stepwise exergonic (ΔΔ*G* = −6.3 kcal mol^−1^) COS release. These findings are consistent with the experimental observations, rationalizing the need for elevated temperature and constant vacuum to yield 2^Ts^ from 3^Ts^, whereas the reaction for the phosphinoyl system proceeds readily at room temperature. The concerted cycloreversion of the tosyl derivative can be rationalized by the instability of the corresponding intermediate INT2^Ts^, which is expected to be less stable than its phosphinoyl analog due to the stronger electron-withdrawing ability of the tosyl group, reducing the nucleophilicity of alkynyl sulfide 2^Ts^.

After successful synthesis of the alkynyl sulfides from CS_2_, we examined whether a similar transformation could also be achieved using other sulfur-containing heteroallenes, such as isothiocyanates. We started our investigation with phenyl isothiocyanate and 1^PO^ as model substrates. Treatment of 1 equiv. of PhNCS with 1^PO^ at room temperature yielded a bright yellow solution. The ^31^P{^1^H} NMR spectrum indicated selective formation of a new species, characterized by a signal appearing at *δ*_P_ = 14.5 ppm. XRD analysis of single crystals grown in presence of 18-c-6 (Fig. 3) identified 4a^PO^ as a four-membered thiete. In contrast to the CS_2_ addition product 3^PO^, which immediately underwent OCS release at room temperature, 4a^PO^ proved to be stable at this temperature, but converted to the alkynyl sulfide anion 2^PO^ upon heating of a THF solution at 60 °C. Mechanistically, formation of 2^PO^ from 4a^PO^ is proposed to proceed through initial rearrangement to azetidine ring (INT3^A^), which subsequently undergoes stepwise [2 + 2] cycloreversion to release PhNCO and thioketenyl anion 2^PO^ (see the SI section 5.5 for details). Since the reaction was not selective, isolation of 2^PO^ from the reaction mixture proved challenging, and was only achieved by crystallization in presence of 18-c-6. Therefore, we examined the use of *tert*-butyl isothiocyanate as alternative CS transfer reagent. While no reaction was observed at room temperature, heating at 45 °C in presence of 1 equiv. of crown ether led to the selective formation of the crown ether complex of 2^PO^, which could be isolated in a good 62% yield.

To assess the generality of this method, we also examined the reactivity of other ketenyl anions with isothiocyanates. Treatment of potassium ketenyl 1^PS^ with PhNCS at room temperature afforded the corresponding four-membered heterocycle 4a^PS^ as a brown powder in a good yield of 82%. Heating the THF solution 4a^PS^ at 60 °C in presence of 1 equiv. of 18-c-6 led to the formation of crown ether complex of 2^PS^ (*δ*_P_ = 10.5 ppm) in moderate yield of 52%. Reaction of ^*t*^BuNCS with 1^PS^ showed no detectable conversion to heterocycle 4b^PS^ at room temperature but directly led to the formation of 2^PS^ upon heating of the reaction mixture in presence of crown ether at 45 °C. Reaction of the tosyl system 1^Ts^ with PhNCS at room temperature afforded the cycloaddition product 4^Ts^ in a good yield of 69% (Fig. 3). Unfortunately, heating a THF solution of 4^Ts^ at 60 °C did not yield detectable amounts of 2^Ts^. These observations demonstrate that alkynyl sulfide formation *via* cycloaddition of ketenyl anions with thioisocyanates and cycloreversion through isocyanates release is a more general approach, which however depends on the applied thioketenyl anion and the used thioisocyanate.

### Spectroscopic and crystallographic studies

With the series of isolable anionic alkynyl sulfides at hand, we conducted a systematic investigation of their structural and spectroscopic properties to elucidate whether these anions are more appropriately described as anionic alkynyl sulfides 2 or thioketenyl anions 2′ (Scheme 1). The anions feature a signal in the range between 51 and 63 ppm in the ^13^C{^1^H} NMR spectrum, corresponding to the *C*1-CS carbon (Table 1). This signal appears significantly high-field shifted relative to those reported for neutral thioketenes (*e.g.*, 86 ppm for (Mes)_2_C=C=S)^51^ and neutral alkynyl sulfides (*e.g.*, 75 ppm for TolC≡CSTol),^52^ indicating the presence of an electron pair on the C1 carbon and supporting the thioketenyl form. However, comparison of the ^13^C{^1^H} NMR spectroscopic data of thioketenyl anion 2^PO^ with those of the corresponding ketenyl anion 1^PO^,^41^ keteniminyl anion 5 ^53^ and diazomethanide 6 ^54^ reveals that 2^PO^ features the most deshielded C1 signal, indicating reduced charge accumulation on the C1 carbon in 2^PO^ relative to the other anions. The IR spectra of 2 display two characteristic CCS stretching bands: an asymmetric stretch at 2009–2024 cm^−1^ and a symmetric stretch at 1950–1957 cm^−1^,^55^ which fall within the range of values reported for neutral thioketenes (around 1750 cm^−1^)^51^ and neutral alkynyl sulfides (around 2100 cm^−1^).^56^ Overall, this suggests an intermediate bonding situation between the thioketenyl 2′ and alkynyl sulfide 2 form, but more shifted towards the alkynyl form.

**Table 1:** Comparison of the spectroscopic and crystallographic data of the alkynyl sulfide anions 2 with related ketenyl anion 1, keteniminyl anion 5 and diazomethanide 6

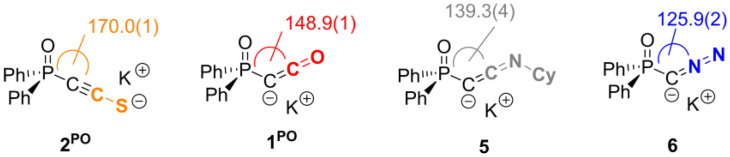						
	2^Ts^	2^PS^	2^PO^	1^PO^ a	5^b^	6^c^
*δ*(C1) (ppm)	62.7	51.6	54.0	3.1	40.2	19.8
*δ*(C2) (ppm)	145.8	140.4	141.6	140.8	151.4	—
^1^ *J*_PC_ (Hz)	—	189.8	207.6	209.6	133.8	57.6
IR (cm^−1^)	2018, 1957	2024, 1957	2009, 1950	2093	1981	1970
C1–C2 (Å)	1.228(9)	1.226(2)	1.223(2)	1.240(2)	1.283(6)	—
C2–S1 (Å)	1.618(6)	1.626(1)	1.631(1)	—	—	—
Z–C1–C2 (°)	157.9(6)	150.9(1)	170.0(1)	148.9(1)	139.3(4)	125.9(2)

a NMR and structure parameters are taken from ref. 41.

b NMR and XRD parameters are taken from ref. 53.

c NMR and structure parameters are taken from ref. 54.

**Fig. 5:**
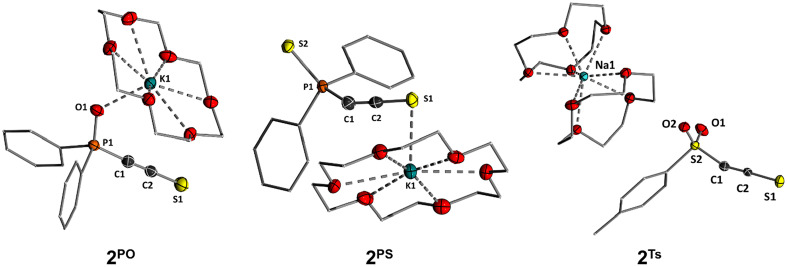
Molecular structures of the crown-ether complexes of the alkynyl sulfide anions 2 in the solid state. Hydrogen atoms and disordered parts in the molecules are omitted for clarity. Thermal ellipsoids are shown at the 50% probability level. Crystallographic details are given in the SI.

The solid-state structures of the alkynyl sulfide anions 2 were elucidated by XRD analysis (Fig. 5). The alkali metal salts were crystallized as crown ether complexes, which feature distinctly different anion cation interactions. In 2^PO^ and 2^PS^, the potassium ion forms direct contacts to the anion, binding to the phosphinoyl group in 2^PO^ and to the sulfur atom of the alkynyl sulfide moiety in 2^PS^. In contrast, 2^Ts^ represents a “free” alkynyl sulfide anion. In this case, the two 12-crown-4 (12-c-4) molecules fully encapsulate the sodium cation, eliminating any direct metal–anion interaction, enabling a discussion of the alkynyl sulfide structure without interference of the counter cation. Regardless of the presence or nature of the cation anion interaction, all structures exhibit an almost linear, yet slightly bent geometry, with E–C1–C2 angles ranging between 150.9(1) and 170.0(1)°. This contrasts the linear geometry of the cyano system predicted by DFT studies, indicating significant substituent effects on the bending angle.^38^ However, the observed trend in the bending angle (2^PS^ < 2^Ts^ < 2^PO^) does not align with typical substituent properties such as electron-withdrawing ability, implying that cation–anion interactions or even packing effects in the crystal structure override simple electronic effects. The C1–C2 and S1–C2 bond lengths vary only slightly in the different alkynyl sulfide anions. The free anion 2^Ts^ exhibits the shortest S1–C2 bond length (1.618(6) Å), which lies between typical C–S single and double bond values, and the longest C1–C2 bond length of 1.228(9) Å, consistent with an elongated C–C triple bond and the most pronounced thioketenyl character in this anion. Comparison of the structural parameters of the alkynyl sulfide anion 2^PO^ with the ketenyl anion 1^PO^, keteniminyl anion 5 and diazomethanide 6 reveals that the bending angle decreases with increasing acceptor ability of the ligand at C1 (CS, CO, CNCy, N_2_) in the order 2^PO^ > 1^PO^ > 5 > 6. Similarly, the C1–C2 bond length increases in the same order from the alkynyl sulfide to the keteniminyl anion. These trends, together with the IR spectroscopic data, clearly confirm the more pronounced contribution of the alkynyl sulfide structure to the bonding situation.

### Computational analysis of the bonding in alkynyl sulfide anions

**Fig. 6:**
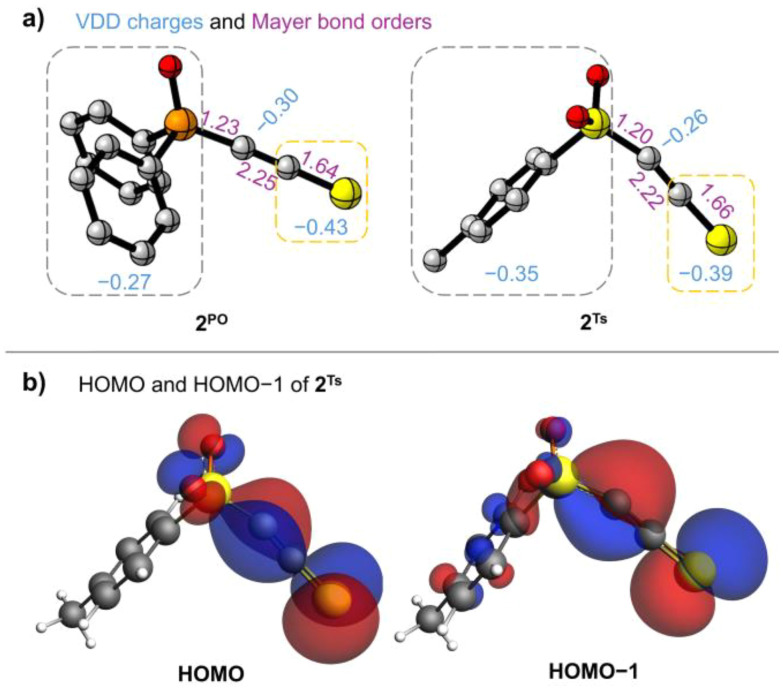
Bonding situation in the alkynyl sulfide anions. (a) Comparison of the Voronoi deformation density (VDD) atomic charges (in electrons) and Mayer bond orders in 2^PO^ and 2^Ts^. (b) Display of the isosurface (at 0.03 au) of the HOMO and HOMO−1 of 2^Ts^. Computed at ZORA-BP86/TZ2P level of theory.

To shed light on the electronic structure of the alkynyl sulfide anions, we carried out DFT calculations at the ZORA-BP86/TZ2P level of theory. To assess the influence of substituents independent of cation effects, we analyzed the free anions 2 [Z–C–CS]^−^ with Z = P(O)Ph_2_ (2^PO^) and Z = SO_2_Tol (2^Ts^). The energy-optimized gas-phase structure of 2^Ts^ shows a more acute bending angle of 161.7° at C1 atom compared to 2^PO^ (176.9°), in agreement with the experimentally observed trend (Table S5). Likewise, the tosyl system also features a shorter C–S bond than 2^PO^ (1.619 and 1.625 Å, respectively), suggesting that the thioketenyl resonance form 2′ (Scheme 1) is more strongly favored in 2^Ts^ than in 2^PO^. This preference is attributed to the stronger electron-withdrawing ability of the tosyl substituent, which more efficiently stabilizes the negative charge at carbon in the thioketenyl form. Accordingly, the calculated Voronoi deformation density (VDD)^57^ atomic charges at both the C1 atom [*Q*(C1) = −0.26] and the CS moiety [*Q*(CS) = −0.39] in 2^Ts^ are less negative than the corresponding values in 2^PO^ (*Q*(C1) = −0.30 and *Q*(CS) = −0.43), reflecting an increased charge transfer to the tosyl group (Fig. 6a). Similarly, the calculated Mayer bond order (MBO)^58,59^ of the C1–C2 bond in 2^Ts^ (2.22) is smaller than in 2^PO^ (2.25), further supporting that more electron-withdrawing substituents favor the thioketenyl resonance form 2′. However, analysis of the frontier orbitals of both anions 2 reveals that the two highest molecular orbitals (HOMO and HOMO−1) in both 2^Ts^ and 2^PO^ (Fig. 6b and Table S6) correspond to the π-bonds of the C–C–S linkage, suggesting a C≡C triple bond, yet with a polarization towards the C1 carbon atom. The LUMO is predominantly localized on the Z group.

After understanding the effect of the Z group, we next analyzed the electronic structure of alkynyl sulfide anion 2^PO^ {[Ph_2_P(O)–C–L2]^−^; L2 = CS} in comparison with the isoelectronic ketenyl anion 1^PO^ {L2 = CO}, keteniminyl anion 5 {L2 = CNCy}, and diazomethanide 6 {L2 = N_2_}. To the best of our knowledge, this compound series represents the only set of isolated compounds featuring a systematic variation of these fundamental ligands and therefore provides general insights into their intrinsic properties. The calculated geometries of the naked anions 2^PO^, 1^PO^, 5, and 6 (Table S5) exhibit bending angles at the C1 atom that follow the trend CS > CO > CNCy > N_2_, in good agreement with the experimental data. This series of anions, featuring bent geometry and negative partial charge accumulation (Table S10) around the C1 atom, shows close resemblance with carbones, where a divalent C(0) atom retains its valence electrons as two lone pairs, and the chemical bonds are best described as dative interactions with concomitant L2 → C ← L2 σ-donation and L2 ← C → L2 π-backdonation.^60,61^ In carbone systems, the Dewar–Chatt–Duncanson model^62,63^ (often used for transition metal complexes) was used to demonstrate that the bending angle around C atom becomes more acute with decreasing π-acceptor strength of the ligands [*e.g.*, C(CO)(CO) (156°); C(CO)(PPh_3_) (152°); C(PPh_3_)(PPh_3_) (146°)].^64^ Similarly, in our case, with decreasing π-acceptor strength of the L2 ligands (CS > CO ≈ CNCy > N_2_),^65–67^ the bending angle around C1 decreases 2^PO^ > 1^PO^ > 5 > 6.

**Fig. 7:**
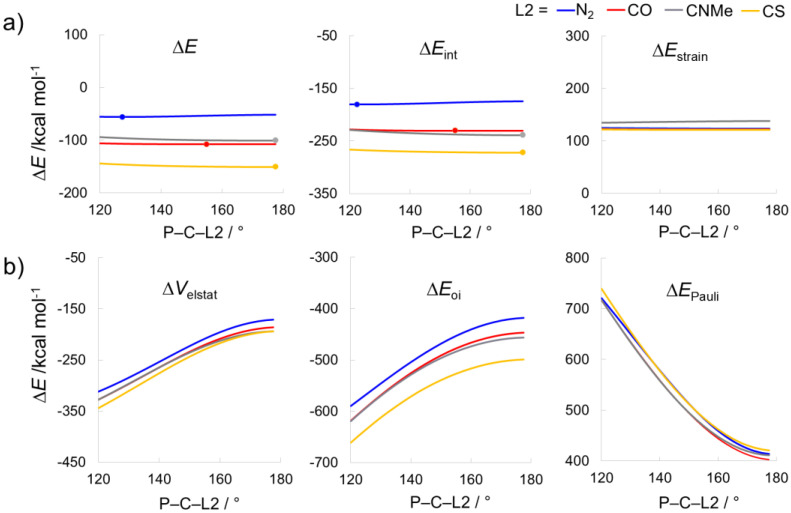
(a) Activation strain model (ASM) and (b) energy decomposition analysis (EDA) of the C–L2 bond in anionic systems [Me_2_P(O)–C–L2]^−^ (where L2 = CS, CO, CNMe, N_2_) as a function of the P–C–L2 bond angle, computed at ZORA-BP86/TZ2P level of theory.

To gain further insight into the nature of the chemical bonding, we analyzed the bonding energy (Δ*E*) between [Me_2_P(O)C]^−^ and L2 [L2 = CS (2^PO^_S_), CO (1^PO^_S_), CNMe (5_S_) and N_2_ (6_S_)] along the bending coordinate around C1, using the Activation Strain Model (ASM)^68–73^ combined with the Energy Decomposition Analysis (EDA)^74–76^ (see the SI for technical details). To reduce computational cost, simplified model systems (2^PO^_S_, 1^PO^_S_, 5_S_, and 6_S_; Fig. S83) were employed, in which all aryl and alkyl substituents were replaced by methyl groups. All trends in C–L2 bond strength observed in the actual system [Ph_2_P(O)–C–L2]^−^ are reproduced in the simplified model systems [Me_2_P(O)–C–L2]^−^ (Tables S5 and S9). The calculated electronic bonding energy (Δ*E*) of the C–L2 bond in [Me_2_P(O)–C–L2]^−^ becomes less stabilizing from CS to CO, CNMe, and N_2_. Note that the potential energy surface as a function of the P–C–L2 is flat, and the trends in Δ*E* remain the same at any given P–C–L2 bending angle. In general, these trends in Δ*E* arise from the trends in the interaction energy Δ*E*_int_ (Fig. 7a). Only for L2 = CNMe (5_S_), the strain energy Δ*E*_strain_ is more destabilizing than for the other L2 ligands, which offsets Δ*E*_int_ and causes a bond weakening for CNMe (5_S_) relative to CO (1^PO^_S_).

Further insights into the interaction energy Δ*E*_int_ are obtained by decomposing it into electrostatic interactions Δ*V*_elstat_, Pauli (steric) repulsion Δ*E*_Pauli_, and stabilizing orbital interactions Δ*E*_oi_ (Fig. 7b). Overall, all EDA terms increase in magnitude as the P–C–L2 bending angle becomes more acute, that is, Δ*E*_Pauli_ becomes more destabilizing, whereas Δ*V*_elstat_ and Δ*E*_oi_ become more stabilizing. Most importantly, the observed trends in Δ*E*_int_ are mainly dictated by the trends in Δ*E*_oi_, and to a lesser extent by Δ*V*_elstat_, which becomes less stabilizing along CS(2^PO^_S_), CNMe(5_S_), CO(1^PO^_S_), and N_2_(6_S_). In contrast, the trends in the Pauli repulsion (Δ*E*_Pauli_) along L2 are inconsistent and, therefore, do not determine the observed trends in bonding energy Δ*E*.

**Fig. 8:**
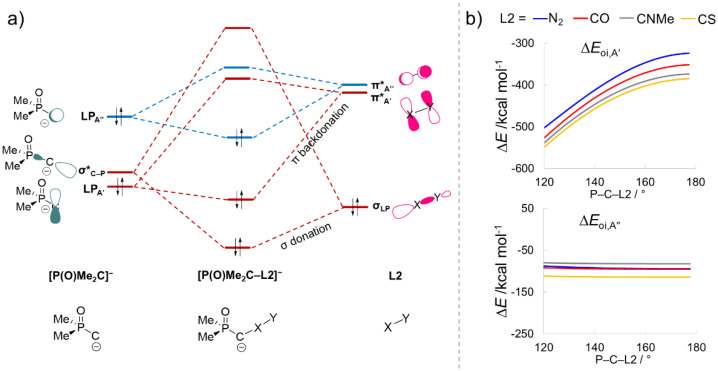
(a) Molecular orbital diagram of the bonding interactions (σ-donation and π-backdonation) between [P(O)Me_2_C]^−^and L2 in [P(O)Me2C–L2]^−^ (where L2 = CS, CO, CNMe, N_2_) at P–C–L2 = 120°. (b) Decomposition of the orbital interaction (Δ*E*_oi_) term in the *A*′ and *A*″ irreducible representations of *C*_S_ symmetry as a function of the P–C–L2 bond angle. All values computed at ZORA-BP86/TZ2P level of theory.

Quantitative molecular orbital analysis reveals a metallomimetic nature of these anionic systems, which are dominated by the [(POMe_2_)C]^−^ ← L2 σ-donation and the in-plane and out-of-plane [(POMe_2_)C]^−^ → L2 π-backdonation, in accordance with the classical Dewar–Chatt–Duncanson model (Fig. 8a). Further decomposition of the orbital-interaction term Δ*E*_oi_ into Δ*E*_oi,*A*′_ and Δ*E*_oi,*A*″_ components reveals that the orbital interactions primarily originate from the interactions in the Δ*E*_oi,*A*′_ (Fig. 8b), which become less stabilizing along CS(2^PO^_S_), CO(1^PO^_S_), CNMe(5_S_), and N_2_(6_S_). The Δ*E*_oi,*A*′_ term includes both [(POMe_2_)C]^−^ ← L2 σ-donation and the in-plane [(POMe_2_)C]^−^ → L2 π-backdonation (depicted in red in Fig. 8a). Analysis at a consistent geometry with the P–C–L2 angle fixed at 120° (Fig. S86) shows that the in-plane π*-antibonding orbital of the L2 ligand rises in energy going from CS to CNMe to CO to N_2_, indicating a reduction of π-acceptor ability of the L2 ligand.

Overall, CS is the best π-acceptor ligand, which experiences the strongest π-backdonation, both in-plane (Δ*E*_oi,*A*′_) and out-of-plane (Δ*E*_oi,*A*″_). The stronger the π-backdonation, the larger the C≡L2 triple bond character. Therefore, the weakening of the π-backdonation from CS(2^PO^_S_), CO(1^PO^_S_), CNMe(5_S_), and N_2_(6_S_) is perfectly in line with the experimentally observed decrease of the bending angle along this series of ligands and confirms that the alkynyl resonance form 2 is predominant for CS.

### Reactivity studies of 2

**Fig. 9:**
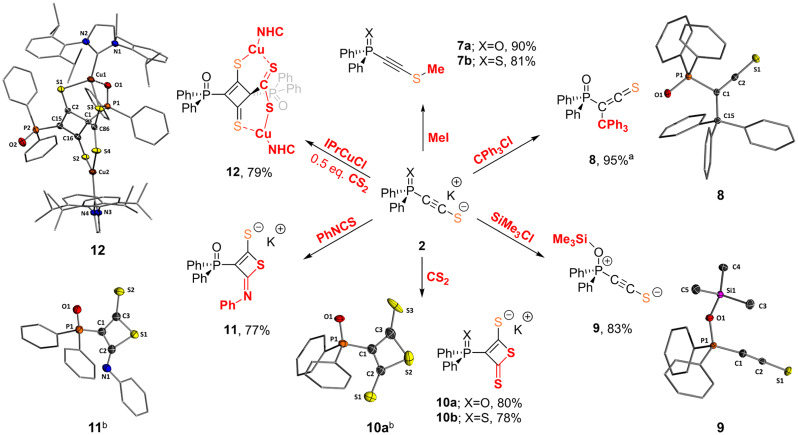
Reactivity studies of anionic alkynyl sulfides 2. For details on reaction conditions, see the SI. For clarity only the anionic component of the salts 10 and 11 are depicted. The cation [K·(18-c-6)], solvents and disordered parts in the solid-state structures have been omitted for clarity. Thermal ellipsoids are shown at the 50% level. ^*a*^ NMR yield; all others are isolated yields. ^*b*^ Crystal data of low quality.

With the first isolable alkynyl sulfide anions at hand, we next investigated their reactivity, in particular the chemoselectivity toward different electrophiles. We particularly focused on reactivity studies of the phosphinoyl substituted alkynyl sulfide 2^PO^ due to its ease of isolation. Reaction of the anions 2 with methyl iodide afforded thioalkynes 7 through selective methylation at the sulfur end. This reactivity contrasts the observations made for ketenyl anions, which showed selective methylation at the C1 carbon center, and underscores the more pronounced alkynyl character. The thioalkynes 7a and 7b were isolated in excellent yields of 90 and 81%, confirming that 2 can indeed serve as an effective precursor for thioalkyne synthesis (Fig. 9).

Following this successful S-centered reaction, we examined whether other electrophiles would promote reactivity at the nucleophile C1 site, thereby enabling access to neutral thioketenes. Indeed, reaction of 2^PO^ with trityl chloride selectively yielded the neutral thioketene 8, indicating that softer electrophiles preferentially react at the C1 carbon atom. Single crystals of compound 8 suitable for XRD analysis were obtained by slow evaporation of benzene solution and unambiguously confirmed the C-centered reactivity. Analysis of the molecular structure of 8 revealed an elongation of the C1–C2 bond from 1.223(2) Å in 2^PO^ to 1.316(2) Å, accompanied by a shortening of the C2–S1 bond from 1.632(1) Å in 2^PO^ to 1.552(1) Å. These changes are consistent with a transformation from a thioalkyne to a thioketene. In contrast to the phosphinoyl-substituted alkynyl sulfide, the tosyl system exhibited low selectivity towards both electrophiles, indicating that excessive stabilizing of the negative charge into the backbone substituent complicates selective transformations.

While trimethylsilyl chloride (TMSCl) showed no selective reaction with both 2^PS^ and 2^Ts^, its reaction with 2^PO^ led to a further reactivity pattern. Addition of 1 equiv. of TMSCl to a THF solution of 2^PO^ at room temperature resulted in the selective formation of new species 9, exhibiting a slightly down-field resonance signal at *δ*_P_ = 8.5 ppm. This signal was assigned to the product of the silylation at the oxygen center of the phosphinoyl group. The strong oxophilicity of the silicon directs the reactivity of TMSCl to the oxygen rather than the carbon or sulfur center (Fig. 9). Compound 9 features an almost linear geometry in the solid state with a P–C–C angle of 165.0(2)° and a C2–C2 distance of 1.232(2) Å.

Besides their reactivity towards electrophiles, the alkali metal salts of 2 showed high reactivity toward unsaturated compounds. Reaction of 2 with carbon disulfide or phenyl isothiocyanate furnished the highly functionalized heterocycles 10 and 11, respectively, *via* [2 + 2] cycloaddition reactions across the C–C triple bond (Fig. 9). Both reactions proceeded selectively, enabling the isolation of the resulting heterocycles as solids in good yields of 77 to 80%. Both heterocycles represent novel scaffolds that, until now, have only been synthesized once as neutral species with either PPh_3_ or an N-heterocyclic carbene ligand as exocyclic substituents.^40,77^ These thiete scaffolds represent untapped functional groups in pharmaceuticals.^78,79^

In addition, we examined whether the alkali metal salts of 2 could also be used for the transmetalation to alkynyl sulfide transition-metal complexes. While no reaction was observed upon treatment of 2^PO^ with 1 equiv. of IPrCuCl (IPr = 1,3-bis(2,6-diisopropylphenyl)imidazol-2-ylidene) at room temperature, addition of 0.5 equiv. of CS_2_ to the reaction mixture in THF led to the formation of a new species, characterized by two low-field shifted ^31^P{^1^H} NMR resonances at *δ*_P_ = 4.60 and 29.73 ppm. Single-crystal XRD analysis revealed the formation of copper complex 12, featuring a cyclobutene-1,3-dithione scaffold with an exocyclic bound CS_2_ moiety. In the complex, each copper center is coordinated by one sulfur atom of the CS_2_ unit and the sulfur atom from the two former alkynyl sulfide anions. Interestingly, cyclobutene-1,3-dithione 12 is not obtained upon treatment of thiete 10a with the copper complex. This suggests that complex 12 instead forms *via* a copper-stabilized CS_2_ adduct of the alkynyl sulfide that undergoes cyclization with a second equivalent of the alkynyl sulfide.

Overall, these reactivity patterns demonstrate that alkynyl sulfide anions – unlike the corresponding ketenyl anions – exhibit a pronounced tendency to react at the sulfur atom, as well as high reactivities toward cyclization reactions involving the C–C triple bond. Thus, these anions serve not only as entry into the diverse chemistry of neutral alkynyl sulfides, but also offer potential for the synthesis of heterocycles.^80,81^

## Conclusions

In conclusion, we report the synthesis of isolable alkynyl sulfide anions through reaction of ketenyl anions with either carbon disulfide or isothiocyanates. Both reactions proceed *via* a [2 + 2] cycloaddition reaction to a four-membered thiete intermediate, which subsequently undergoes cycloreversion through elimination of carbonyl sulfide and isocyanates, respectively. The electronic structure of these anions is best represented by the alkynyl sulfide form, featuring a C–C triple bond, as supported by the IR and NMR data. However, a minor contribution from the thioketenyl structure causes a slight bending of [R–C–CS]^−^ linkage. This differs from previously reported related anions of the type [R–C–L]^−^, such as ketenyl anions (L = CO) or diazomethanides (L = N_2_), which exhibit a strong contribution of the structure with a lone pair at carbon. DFT studies reveal that the trends in the bonding situation in these anions are primarily governed by orbital interactions, particularly the in-plane π-backdonation from carbon to ligand L. This backdonation increases from N_2_ to CO and CS, reflecting fundamental ligand properties observed in transition metal chemistry, thereby unifying bonding concepts between carbanionic compounds and coordination complexes. The distinctive bonding in alkynyl anions is reflected in their reactivity patterns, which include a pronounced tendency for S-centered reactivity relative to the corresponding ketenyl anions and addition reactions across the C–C triple bond. These unique reactivities render alkynyl sulfides as powerful reagents for the synthesis of sulfur-containing heterocycles.

## Author contributions

S. M. performed all synthetic experiments and spectroscopic analyses, except for compound 3^Ts^, 2^Ts^ and 10b, which were synthesized by Q. L., A. D. F. helped with the isolation of compound 7 and 9. Q. L. carried out the *in situ* IR experiments. V. H. G. designed and supervised the experimental part of the project. S. M. carried out the quantum chemical calculations with help of D. A. S and D. R. S. The theoretical part of the work was supervised by F. M. B.

## Conflicts of interest

There are no conflicts to declare.

## Supplementary Material

QI-013-D6QI01030J-s001

QI-013-D6QI01030J-s002

QI-013-D6QI01030J-s003

## Data Availability

The data supporting this article has been included as part of the supplementary information (SI). Supplementary information: the primary experimental data (NMR, IR files) as well as the coordinates of the optimized structures (xyz). See DOI: https://doi.org/10.1039/d6qi01030j. CCDC 2528269–2528277 contain the supplementary crystallographic data for this paper.^82*a*–*i*^ The CCDC numbers and compound assignments are provided in the tables in the SI. We have included these cif files and the corresponding checkcif reports.
