# Supplementary material for: Synthesis, structural analysis and reactivity of alkynyl sulfide anions
Source: Inorg Chem Front. 2026 Jul 10. Online ahead of print. doi: 10.1039/d6qi01030j (PMC13383285; doi:10.1039/d6qi01030j)
Supplement: QI-OLF-D6QI01030J-s001 [file QI-OLF-D6QI01030J-s001.pdf]

Supplementary Information (SI) for Inorganic Chemistry Frontiers.
This journal is © the Partner Organisations 2026

Supporting Information

Synthesis, Structural Analysis and Reactivity of Non-Coordinated Alkynyl Sulfide Anions

Sunita Mondal+,[1,2] Quentin Le Dé+,[1] Daniel A. Santos Oliveira,[2,3] Daniela Rodrigues Silva,[2] Angela De Feudis,[1] F. Matthias Bickelhaupt,*[2,4,5] Viktoria H. Gessner*[1]

[1]Faculty of Chemistry and Biochemistry, Ruhr-University Bochum, Universitätsstrasse 150, 44801 Bochum, Germany

[2]Department of Chemistry and Pharmaceutical Sciences, Vrije Universiteit Amsterdam, De Boelelaan 1108, 1081 HZ Amsterdam, The Netherlands

[3]Department of Fundamental Chemistry, Institute of Chemistry, University of São Paulo, Av. Prof. Lineu Prestes, 748, São Paulo, 055508-000, Brazil

[4]Institute of Molecules and Materials, Radboud University Nijmegen, Heyendaalseweg 135, 6525 AJ Nijmegen, The Netherlands

[5]Department of Chemical Sciences , University of Johannesburg, Auckland Park, Johannesburg 2006, South Africa

*Correspondence: viktoria.gessner@rub.de; f.m.bickelhaupt@vu.nl

Table of Contents

1. Experimental Procedures: 3

1.1. General Information: 3

1.2. Synthesis of compounds 4 4

1.3. Synthesis of compounds 3 6

1.4. Synthesis of compounds 2^{PO} 6

1.5. Synthesis of compounds 2^{PS} 9

1.6. Synthesis of compounds 2^{Ts} 12

1.7. Synthesis of compounds 7 12

1.8. Synthesis of compounds 8 13

1.9. Synthesis of compounds 9 14

1.10. Synthesis of compounds 10 15

1.11. Synthesis of compounds 11 16

1.12. Synthesis of compound 12 16

2. NMR and IR spectra 18

3. Real-Time IR Spectroscopy 52

4. Crystal structure determination 54

4.1. General information 54

4.2. Molecular structure of 4 59

4.3. Molecular structure of 3 60

4.4. Molecular structure of 2 61

4.5. Molecular structure of 8 62

4.6. Molecular structure of 9 63

4.7. Molecular structure of 10a 63

4.8. Molecular structure of 11 64

4.9. Molecular structure of 12 64

5. DFT Calculations 65

5.1. Computational details 65

5.2. Activation strain model and energy decomposition analysis 65

5.3. Results from structure optimization 67

5.4. Results from Bonding Analysis 72

5.5. Calculated Mechanism for the formation of 2 75

6. References 77

The original data obtained from all analysis methods have been deposited in a repository and can be downloaded via the following link. The only exceptions are the data from the single-crystal X-ray diffraction experiments. The CIF files for these experiments have been deposited with the Cambridge Crystallographic Data Centre (CCDC) (see chapter 4 for details).

Link: https://doi.org/10.17877/RESOLV-2026-LQWZBR

1. Experimental Procedures:

1.1. General Information:

Chemical and Conditions

If not stated otherwise, all experiments were carried out using standard Schlenk techniques under an argon atmosphere, which was dry and free of oxygen. Argon (99.999%) was a product of *Air Liquide* and was used without any further drying. An MBraun SPS 800 was used to dry solvents before their usage (THF, toluene, DCM, ACN, *n*-pentane, *n*-hexane). All solvents were stored over molecular sieves under an argon atmosphere. Reagents were purchased from Sigma-Aldrich, ABCR, Acros Organics or TCI Chemicals and used without further purification if not stated otherwise. **1^{PO}**,[1] **1^{PS}**[2] and **1^{Ts}**[3] were synthesized following literature procedures.

Caution! Strong bases such as organopotassium bases, especially as neat compounds, are severely air-/moisture-sensitive and pyrophoric organometallic compounds. These compounds need to be handled under an inert gas atmosphere to exclude reactions with oxygen and water. Guidelines for their handling can be found in literature: T. L. Rathman, J. A. Schwindeman, *Org. Process Res. Dev.* **2014**, *18*, 1192

Analytical methods

NMR Spectroscopy. ^{1}H, $^{13}C\{^{1}H\}$, $^{31}P\{^{1}H\}$ NMR spectra were recorded on Avance-III-400 spectrometers at 22 °C if not stated otherwise. All values of the chemical shift are in ppm regarding the δ-scale. All spin-spin coupling constants (*J*) are printed in Hertz (Hz). To display multiplicities and signal forms correctly the following abbreviations were used: s = singlet, d = doublet, t = triplet m = multiplet, dd = doublet of doublet, br = broad signal. Signal assignment was supported by, HSQC (^{1}H / ^{13}C), HMBC (^{1}H / ^{13}C, ^{1}H / ^{31}P) correlation experiments. The measurement conditions for each spectrum are provided in the corresponding figures included in Chapter 2.

IR spectra were recorded on a Shimadzu IRSpirit with QATR-S module in an argon filled glovebox. Measurement and processing details for all spectra: Temperature: 22 °C; Apodization function – Happ-Genzel, No. of Scans – 40, Resolution – 2 cm^{-1}.

Real-time, in situ infrared (IR) measurement were performed using a Mettler-Toledo ReactIR™ spectrophotometer equipped with a SiComp silicon ATR probe immerged in the reaction mixture. Data were acquired and treated with the iCIR software developed by the same company.

Elemental analyses were performed on an Elementar vario MICRO cube elemental analyzer in our in-house facility.

HRMS-LIFDI MS A JEOL AccuTof GCv (JMS-T100GCV) (JEOL, Tokyo, Japan) was equipped with a LIFDI source from Linden (CMS, Weyhe, Germany). The emitter heating current was set to 20 mA min-1 at a constant rate.

For details about the single-crystal Xray diffraction analyses, see chapter 4.

1.2. Synthesis of compounds 4

Synthesis of compounds 4a^PO

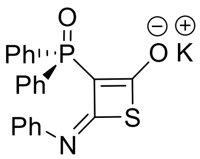

180 mg (0.642 mmol) compound **1^PO** and 88.6 mg (0.642 mmol) phenyl isothiocyanate were dissolved in 5 ml THF and stirred overnight. The clear solution obtained was concentrated to half its volume and 5 mL pentane was added, which resulted in the precipitation of a solid. The solid was filtered and washed with pentane (2 x 5 mL). After drying in vacuo, **4a^PO** was obtained as a light brown colour solid (221 mg, 0.532 mmol, 83%). Single crystals suitable for X-ray diffraction analysis were grown by slow vapor diffusion of pentane into a saturated solution of **4a^PO** in THF at room temperature.

^{31}P{^{1}H}-NMR (162 MHz, THF-d_8): δ = 11.09 (s, *P*Ph_2O) ppm.

^{1}H-NMR (400 MHz, THF-d_8): δ = 8.02 – 7.95 (m, 4H, PC$H_{Ph,ortho}$), 7.37 – 7.32 (m, 6H, PC$H_{Ph,meta,para}$), 7.13 (t, $^{3}J_{HH}$ = 7.3 Hz, 2H, NC$H_{Ph,ortho}$), 6.96 (d, $^{3}J_{HH}$ = 7.0 Hz, 2H, NC$H_{Ph,meta}$), 6.89 (t, $^{3}J_{HH}$ = 7.3 Hz, 1H, NC$H_{Ph,para}$) ppm.

^{13}C{^{1}H}-NMR (101 MHz, THF-d_8): δ = 174.05(s, PC*C*O), 154.03 (d, $^{2}J_{CP}$ = 9.5 Hz, PC*C*N), 153.01 (s, N$C_{Ph,ipso}$), 139.15 (d, $^{1}J_{CP}$ = 104.6 Hz, P$C_{Ph,ipso}$), 132.10 (d, $^{2}J_{CP}$ = 11.0 Hz, P*C*$H_{Ph,ortho}$), 130.97 (d, $^{4}J_{CP}$ = 2.7 Hz, P*C*$H_{Ph,para}$),128.90 (s, N*C*$H_{Ph,ortho}$), 128.38 (d, $^{3}J_{CP}$ = 12.1 Hz, P*C*$H_{Ph,meta}$), 123.17 (s, N*C*$H_{Ph,para}$), 122.66 (s, N*C*$H_{Ph,meta}$), 88.61 (d, $^{1}J_{CP}$ = 110.4 Hz, P*C*CO) ppm.

FT-IR (ATR, cm^{-1}): 2962.8 (m), 1718.4 (bw), 1612.8 (s), 1581.2 (s), 1484.3 (w), 1294.7 (bw), 1258.8 (s), 1012.5 (s), 863.1 (w), 749.0 (w), 690.79 (s), 568.0 (w), 538.6 (s), 525.6 (s).

Anal. Calcd for $C_{21}H_{15}KNO_2PS$: C, 60.71; H, 3.64; N, 3.37; S, 7.72. Found: C, 60.52; H, 3.70; N, 3.05; S, 6.21.

Synthesis of compounds 4a^PS

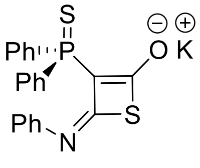

100 mg (0.337 mmol) compound **1^PS** and 46.5 mg (0.337 mmol) phenyl isothiocyanate were dissolved in 5 ml THF and stirred overnight. The clear solution obtained was concentrated to half it's volume and 5 mL pentane was added, which resulted in the precipitation of a solid. The solid was filtered and washed with pentane (2 x 5 mL). After drying in vacuo, **4a^PS** was obtained as a light brown colour solid (120 mg, 0.278 mmol, 82%).

^{31}P{^{1}H}-NMR (162 MHz, THF-d_8): δ = 14.54 (s, *P*Ph$_2$S) ppm.

^{1}H-NMR (400 MHz, THF-d_8): δ = 8.07 – 8.01 (m, 4H, PC*H*$_{Ph,ortho}$), 7.35 – 7.28 (m, 6H, PC*H*$_{Ph,meta,para}$), 7.10(t, $^{3}J_{HH}$ = 7.8 Hz, 2H, NC*H*$_{Ph,ortho}$), 6.88 – 6.84(m, 3H, NC*H*$_{Ph,meta,para}$) ppm.

^{13}C{^{1}H}-NMR (101 MHz, THF-d_8): δ = 174.58(d, $^{2}J_{CP}$ = 8.0 Hz, PC*C*O), 152.75 (d, $^{2}J_{CP}$ = 6.5 Hz, PC*C*N), 152.71 (s, N*C*$_{Ph,ipso}$), 138.87 (d, $^{1}J_{CP}$ = 87.1 Hz, P*C*$_{Ph,ipso}$), 132.97 (d, $^{2}J_{CP}$ = 11.9 Hz, P*C*H$_{Ph,ortho}$), 130.56 (d, $^{4}J_{CP}$ = 3.1 Hz, P*C*H$_{Ph,para}$),128.83 (s, N*C*H$_{Ph,ortho}$), 128.06 (d, $^{3}J_{CP}$ = 12.7 Hz, P*C*H$_{Ph,meta}$), 123.18 (s, N*C*H$_{Ph,para}$), 122.51 (s, N*C*H$_{Ph,meta}$), 85.44 (d, $^{1}J_{CP}$ = 86.5 Hz, P*C*CO) ppm.

FT-IR (ATR, cm^{-1}): 2962.8 (bw), 1717.6 (bw), 1600.0 (s), 1579,8 (s), 1482.1 (m), 1434.7 (s), 1330.6 (bw), 1259.5 (s), 1069.9 (m), 998.1 (m), 902.6 (w), 797.8 (s), 689.4 (s), 563.0 (w),487.6 (s)

Anal. Calcd for $C_{21}H_{15}KNOPS_2$: C, 58.45; H, 3.50; N, 3.25; S, 14.86. Found: C, 58.53; H, 3.54; N, 3.58; S, 14.06.

Synthesis of compounds 4^{Ts}

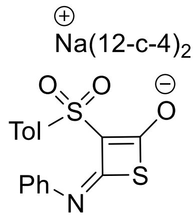

200 mg (0.350 mmol) compound **1^{Ts}** and 48.3 mg (0.350 mmol) phenyl isothiocyanate were dissolved in 5 ml THF and stirred overnight. The clear solution obtained was concentrated to half it's volume and 5 mL pentane was added, which resulted in the precipitation of a solid. The solid was filtered and washed with pentane (2 x 5 mL). After drying in vacuo, **4^{Ts}** was obtained as a light brown colour solid (170 mg, 0.241 mmol, 69%).

^{1}H-NMR (400 MHz, THF-d_8): δ = 7.94 (d, $^{3}J_{HH}$ = 8.1 Hz, 2H, C*H*$_{tol,ortho}$), 7.20 (d, $^{3}J_{HH}$ = 8.0 Hz, 2H, C*H*$_{tol,meta}$), 7.15 – 7.10 (m, 2H, NC*H*$_{Ph,ortho}$), 6.96 (d, $^{3}J_{HH}$ = 7.4 Hz, 2H, NC*H*$_{Ph,meta}$), 6.89 (t, $^{3}J_{HH}$ = 7.3 Hz, 1H, NC*H*$_{Ph,para}$) 3.64 (s, 32H, C*H*$_{2,crown}$ + residual THF), 2.35 (s, 3H, C*H*$_3$) ppm.

^{13}C{^{1}H}-NMR (101 MHz, THF-d_8): δ = 169.5 (s, *C*=O), 152.7 (s, N*C*$_{Ph,ipso}$), 150.3 (s, *C*=N), 145.3 (s, *C*$_{tol,para}$), 141.5 (s, *C*$_{tol,ipso}$), 129.4 (s, *C*H$_{tol,meta}$), 128.9 (s, NCH$_{Ph,ortho}$), 127.2 (s, *C*H$_{tol,ortho}$), 123.4 (s, N*C*$_{Ph,para}$), 122.8 (s, N*C*H$_{Ph,meta}$), 95.9 (s, *C*–SO$_2$tol), 69.0 (s, C$_{crown}$), 21.5 (s, *C*H$_3$) ppm.

FT-IR (ATR, cm^{-1}): 2960.6 (bw),2909.7 (bw), 2866.6 (bw), 1751.4 (m), 1635.1 (s), 1586.2 (s), 1485.0 (m), 1399.5 (w), 1132.4 (s), 1091.5 (s),1020.4 (s), 705.2 (w), 608.9 (w), 556.5 (m), 537.1 (s).

Anal. Calcd for $C_{32}H_{44}NNaO_{11}S_2$: C, 54.45; H, 6.28; N, 1.98; S, 9.09. Found: C, 54.72; H, 6.08; N, 1.85; S, 8.45.

1.3. Synthesis of compounds 3

Synthesis of compounds 3^{Ts}

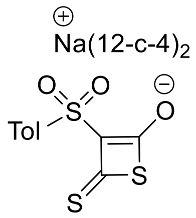

1^{Ts} (100 mg, 0.175 mmol, 1eq) was dissolved in 4 mL of DCM/thf (3:1) mixture and, then, CS_2 (10.6 µL, 0.175 mmol, 1eq) was added. The solution was stirred for 10 min and was crystallized by pentane diffusion to obtain $\mathbf{3^{Ts}}$ as orange crystals (97 mg, 0.150 mmol, 86%).

^{1}H-NMR (400 MHz, CD_2Cl_2): δ = 7.85 (d, $^3J_{HH}$ = 8.3 Hz, 2H, C$H_{tol,ortho}$), 7.25 (d, $^3J_{HH}$ = 7.8 Hz, 2H, C$H_{tol,meta}$), 3.65 (s, 32H, C$H_{2,crown}$+ residual THF), 2.38 (s, 3H, CH_3) ppm.

^{13}C{^{1}H}-NMR (101 MHz, CD_2Cl_2): δ = 200.3 (s, *C*=S), 169.3 (s, *C*=O), 142.7 (s, $C_{tol,para}$), 142.4 (s, $C_{tol,ipso}$), 129.4 (s, $CH_{tol,meta}$), 126.8 (s, $CH_{tol,ortho}$), 114.3 (s, *C*–SO_2tol), 65.9 (s, C_{crown}), 21.6 (s, CH_3) ppm.

FT-IR (ATR, cm^{-1}): 2961.3.0 (bw),2908.8 (bw), 2067.2 (bw), 1747.0 (s), 1653.7 (bw), 1594.8 (w), 1345.6 (s), 1301.1 (m), 1287.9 (m), 1091.4 (s), 1020.4 (s), 993.1(m), 849.5 (s), 575.2 (s), 527.8 (s).

Our repeated attempts on obtaining satisfactory elemental analysis data were unsuccessful.

1.4. Synthesis of compounds 2^{PO}

<u>Pathway 1</u>

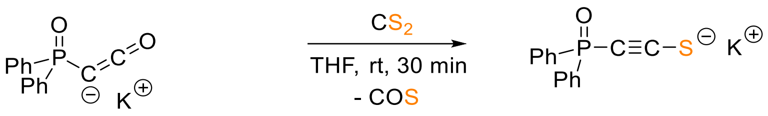

$\mathbf{1^{PO}}$ $\mathbf{2^{PO}}$

<u>Without crown ether</u>: $\mathbf{1^{PO}}$ (40 mg, 0.143 mmol, 1eq) was dissolved in 2 mL of THF and then, CS_2 (8.6 µL, 0.143 mmol, 1eq) was added. After 30 min of stirring, the volatiles were removed in vacuo to yield $\mathbf{2^{PO}}$ as a yellow solid (41 mg, 0.138 mmol, 97%).

<u>With crown ether</u>: $\mathbf{1^{PO}}$ (41 mg, 0.146 mmol, 1eq) and 18-c-6 (38.6 mg, 0.146 mmol, 1eq) were dissolved in 2 mL of THF. Then, CS_2 (8.8 µL, 0.146 mmol, 1eq) was added and stirred for 30 min. Vapour diffusion of pentane into the THF solution yielded $\mathbf{2^{PO}}$ **(18-c-6)** as orange crystals (80 mg, 0.143 mmol, 98%).

Pathway 2 (using Ph–NCS)

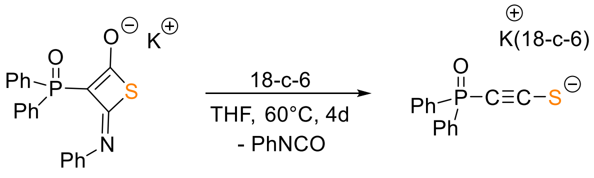

4a^PO　　**2^PO (18-c-6)**

4a^{PO} (40 mg, 0.096 mmol, 1eq) and 18-c-6 (25.4 mg, 0.096 mmol, 1eq) were dissolved in 3 mL of THF. After 4 days of heating at 60°C, the solution obtained was concentrated to half its volume. Vapour diffusion of pentane into the THF solution yielded **2^{PO} (18-c-6)** as orange crystals (22 mg, 0.039 mmol, 41%).

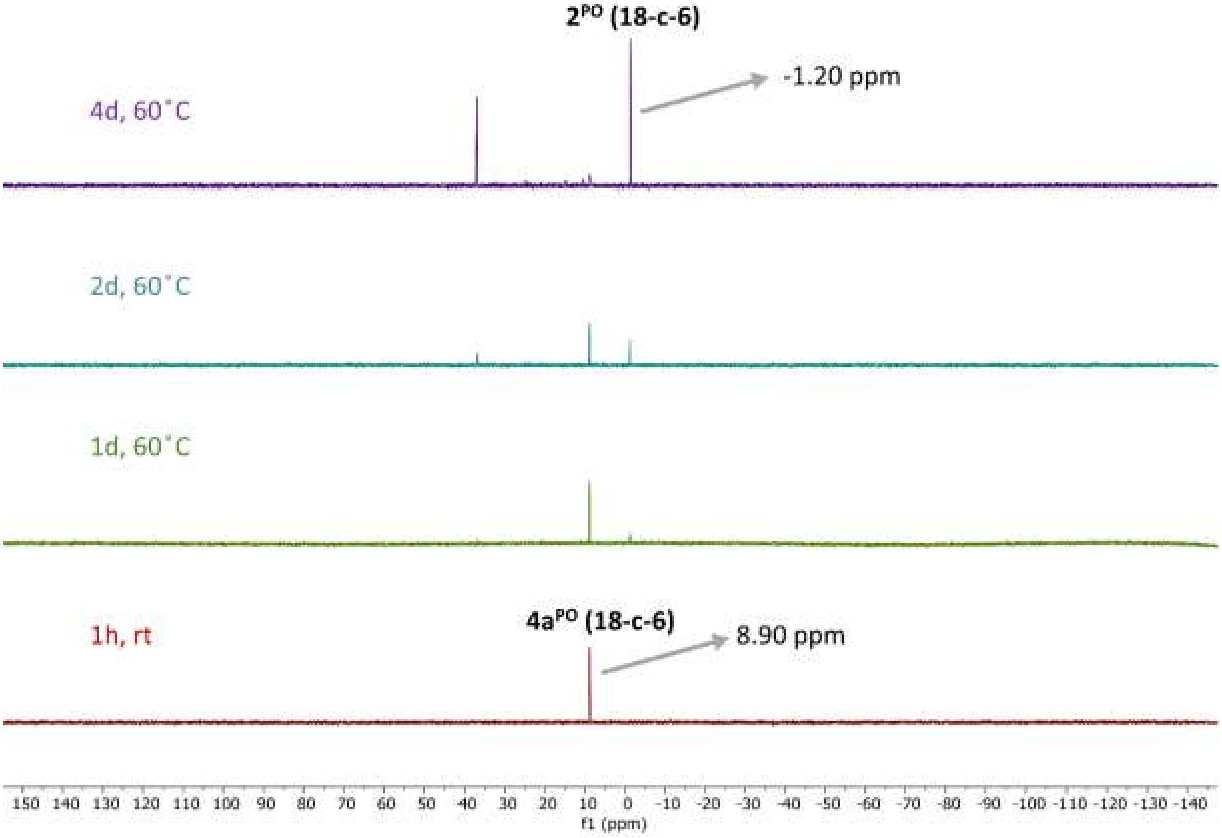

Figure S1 Monitoring of the conversion of **4a^{PO}** into **2^{PO}** by $^{31}P\{^1H\}$ NMR spectroscopy in THF.

Pathway 2 (using ^{t}Bu–NCS)

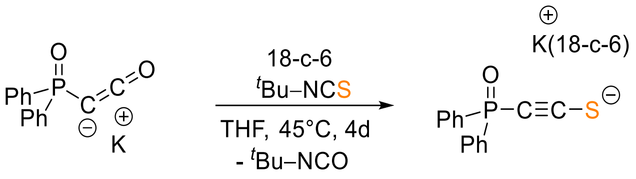

$\mathbf{1^{PO}}$ $\mathbf{2^{PO}}$ **(18-c-6)**

$\mathbf{2^{PO}}$ (30 mg, 0.107 mmol, 1eq) and 18-c-6 (28.3 mg, 0.107 mmol, 1eq) were dissolved in 3 mL of THF and then, ^{t}Bu-NCS (13.7 µL, 0.107 mmol, 1eq) was added. After 4 days of heating at 45° C, the solution obtained was concentrated to half its volume. Vapour diffusion of pentane into the THF solution yielded $\mathbf{2^{PO}}$ **(18-c-6)** as orange crystals (37 mg, 0.066 mmol, 62%).

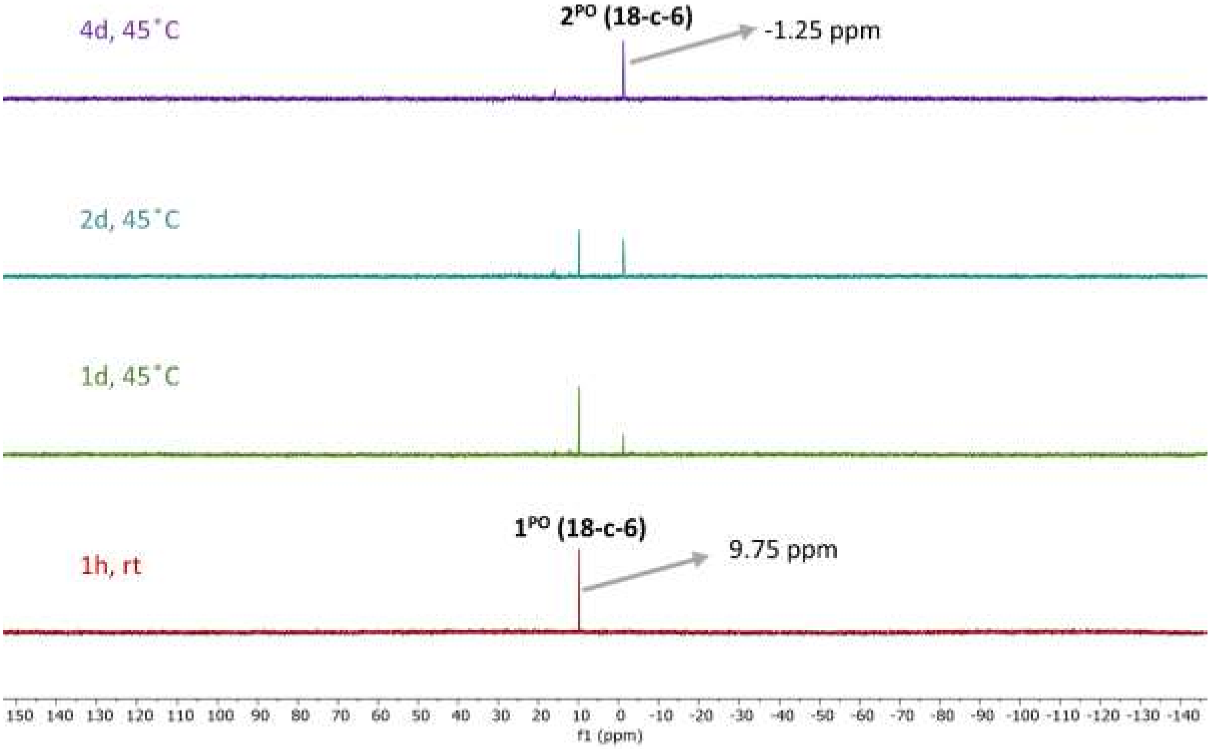

Figure S2 Monitoring of the conversion of $\mathbf{1^{PO}}$ into $\mathbf{2^{PO}}$ upon reaction with ^{t}BuNCS by ^{31}P{^{1}H} NMR spectroscopy in THF.

Without crown ether:

^{31}P{^{1}H}-NMR (162 MHz, THF-d_8): δ = 2.44 (s, *P*Ph$_2$O) ppm.

^{1}H-NMR (400 MHz, THF-d_8): δ = 7.88 – 7.82 (m, 4H, C*H*$_{\text{Ph,}ortho}$), 7.35 – 7.25 (m, 6H, C*H*$_{\text{Ph,}meta,para}$) ppm.

^{13}C{^{1}H}-NMR (101 MHz, THF-d_8): δ = 141.63(d, $^{2}J_{CP}$ = 39.9 Hz, PC*C*S), 140.20 (d, $^{1}J_{CP}$ = 119.9 Hz, *C*$_{\text{Ph,}ipso}$), 131.66 (d, $^{2}J_{CP}$ = 10.9 Hz, *C*H$_{\text{Ph,}ortho}$), 130.78 (d, $^{4}J_{CP}$ = 2.7 Hz, *C*H$_{\text{Ph,}para}$),128.53 (d, $^{3}J_{CP}$ = 12.8 Hz, *C*H$_{\text{Ph,}meta}$), 54.01 (d, $^{1}J_{CP}$ = 207.6 Hz, P*C*CS) ppm.

With crown ether:

^{31}P{^{1}H}-NMR (162 MHz, CD_2Cl_2): δ = 1.18 (s, *P*Ph_2O) ppm.

^{1}H-NMR (400 MHz, CD_2Cl_2): δ = 7.87 – 7.81 (m, 4H, $CH_{Ph,ortho}$), 7.39 – 7.36 (m, 6H, $CH_{Ph,meta,para}$), 3.59 (s, 32H, $CH_{2,crown}$) ppm.

^{13}C{^{1}H}-NMR (101 MHz, CD_2Cl_2): δ = 139.33 (d, $^{1}J_{CP}$ = 119.3 Hz, $C_{Ph,ipso}$), 135.41(d, $^{2}J_{CP}$ = 39.6 Hz, PC*C*S), 131.14 (d, $^{2}J_{CP}$ = 10.8 Hz, $CH_{Ph,ortho}$), 130.48 (d, $^{4}J_{CP}$ = 2.8 Hz, $CH_{Ph,para}$),128.20 (d, $^{3}J_{CP}$ = 12.6 Hz, $CH_{Ph,meta}$), 70.43 (s, C_{crown}), 56.60 (d, $^{1}J_{CP}$ = 211.7 Hz, P*C*CS) ppm.

FT-IR (ATR, cm^{-1}): 2905.2 (bw), 2866.45 (bw), 2009.1 (s, CCS asym. stretching), 1949.5 (s, CCS sym. stretching), 1617.1 (bw), 1435.4 (s), 1349.9 (s), 1283.9(w), 1098.6 (s), 990.9 (w), 957.9 (s), 863.8 (m), 831.5 (m), 533.5 (s), 458.1 (s).

Anal. Calcd for $C_{26}H_{34}KO_7PS$: C,55.70; H, 6.11; S,5.72. Found: C, 55.70; H, 6.08; S, 4.80.

1.5. Synthesis of compounds 2^{PS}

Pathway 1

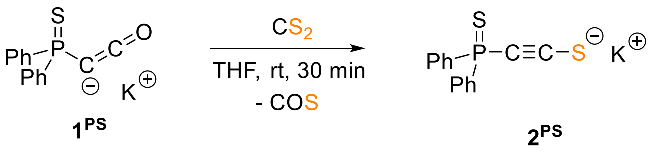

Without crown ether: **1^{PS}** (40 mg, 0.135 mmol, 1eq) was dissolved in 2 mL of THF and then, CS_2 (8.2 µL, 0.135 mmol, 1eq) was added. After 30 min of stirring, the volatiles were removed in vacuo to yield **2^{PS}** as a yellow solid (41 mg, 0.131 mmol, 97%).

With crown ether: **1^{PS}** (150 mg, 0.506 mmol, 1eq) and 18-c-6 (134 mg, 0.506 mmol, 1eq) were dissolved in 5 mL of THF. Then, CS_2 (30.6 µL, 0.506 mmol, 1eq) was added and the THF solution was stirred for 30 min. The solution was crystallized by pentane diffusion to obtain **2^{PS} (18-c-6)** as orange crystals (172 mg, 0.298 mmol, 59%).

Pathway 2 (Using Ph–NCS)

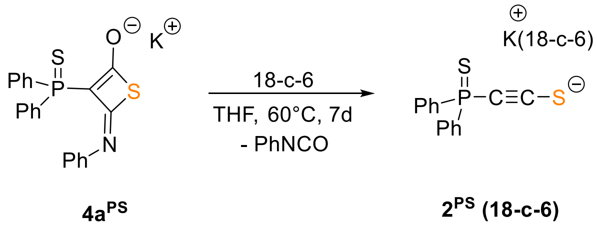

$4a^{PS}$ (40 mg, 0.093 mmol, 1eq) and 18-c-6 (24.5 mg, 0.093 mmol, 1eq) were dissolved in 3 mL of THF. After 7 days of heating at 60°C, the solution obtained was concentrated to half its volume. Vapour diffusion of pentane into the THF solution yielded **2^{PS} (18-c-6)** as orange crystals (28 mg, 0.048 mmol, 52%).

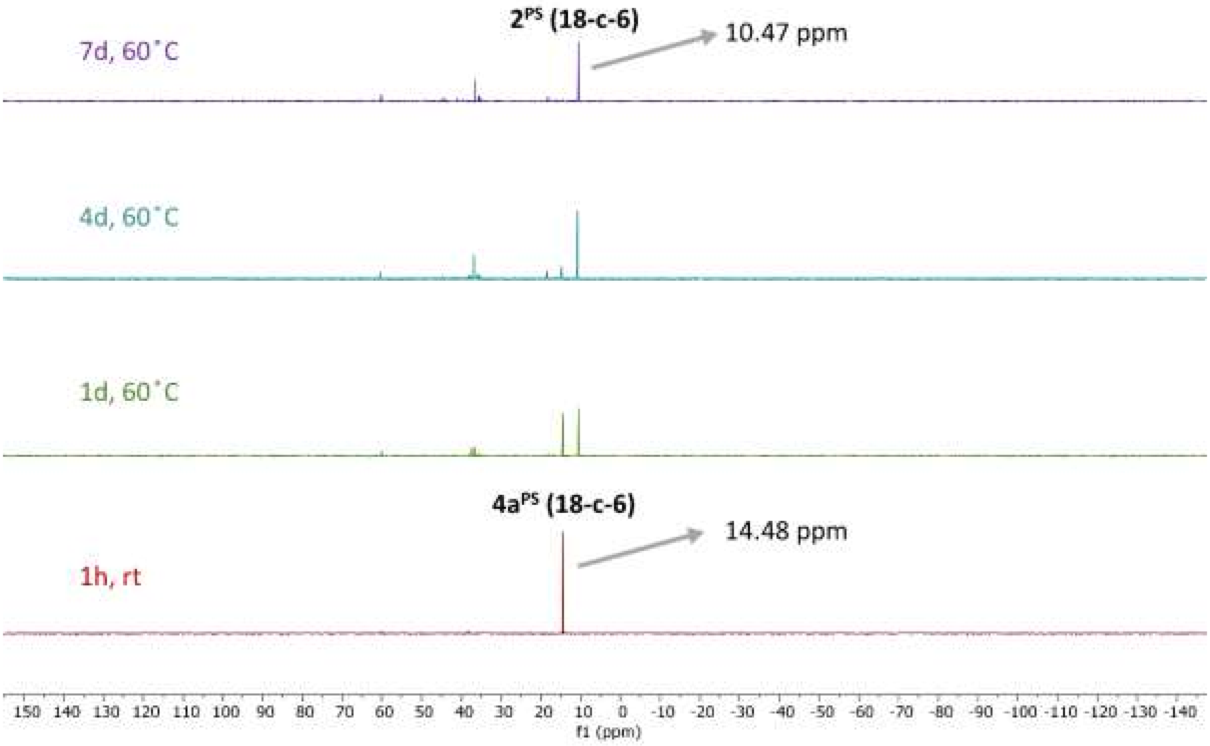

Figure S3 Monitoring of the conversion of **4a^{PS}** into **2^{PS}** by ^{31}P{^{1}H} NMR spectroscopy in THF.

<u>Pathway 2</u> (Using ^{t}Bu-NCS)

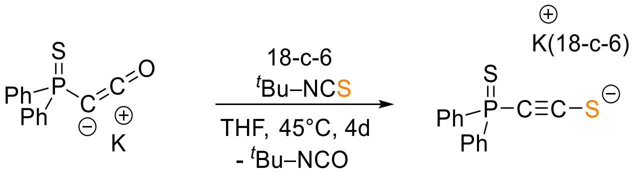

1^{PS} **2^{PS} (18-c-6)**

1a^{PS} (40 mg, 0.135 mmol, 1eq) and 18-c-6 (35.7 mg, 0.135 mmol, 1eq) were dissolved in 3 mL of THF and then, ^{t}Bu-NCS (17.3 µL, 0.135 mmol, 1eq) was added. After 4 days of heating at 45°C, the solution obtained was concentrated to half its volume. Vapour diffusion of pentane into the THF solution yielded **2^{PS} (18-c-6)** as orange crystals (27 mg, 0.047 mmol, 35%).

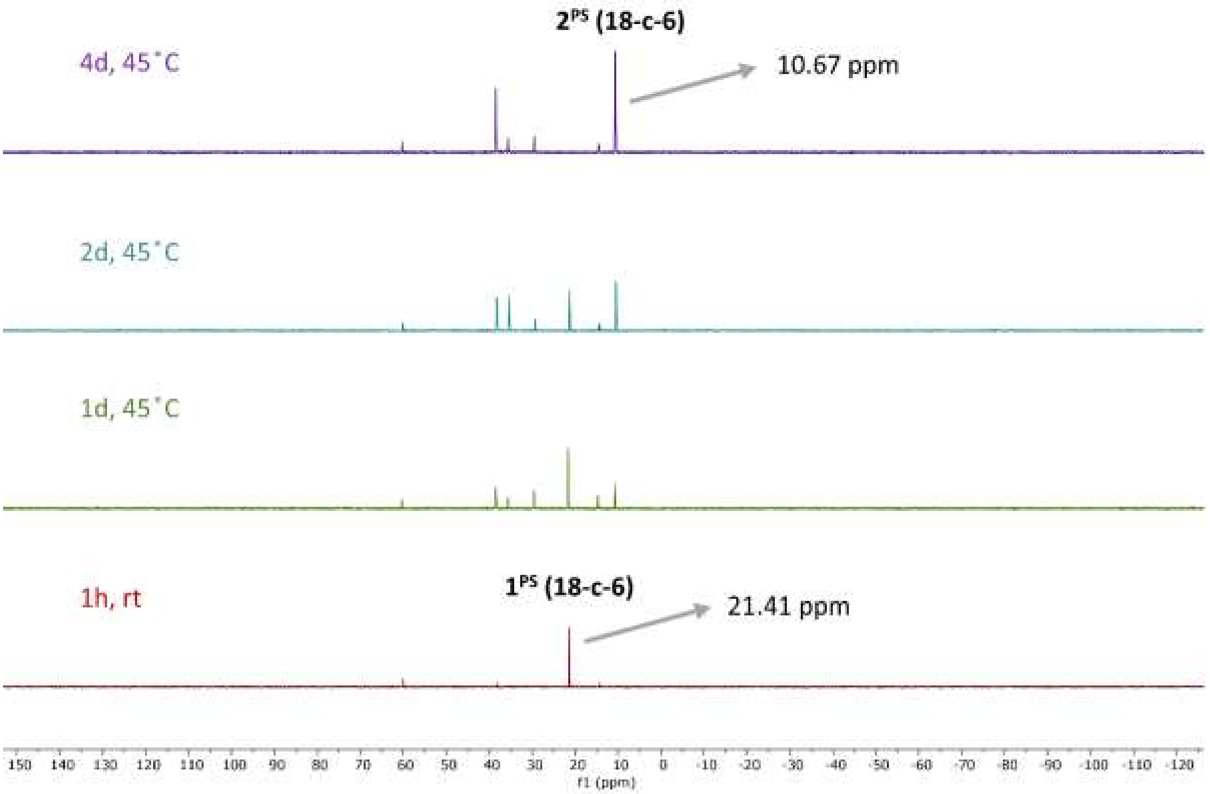

Figure S4 Monitoring of the conversion of $\mathbf{1^{PS}}$ into $\mathbf{2^{PS}}$ upon reaction with ^{t}BuNCS by ^{31}P{^{1}H} NMR spectroscopy in THF.

Without crown ether

^{31}P{^{1}H}-NMR (162 MHz, THF-d_8): δ = 12.48 (s, *P*Ph$_2$S) ppm.

^{1}H-NMR (400 MHz, THF-d_8): δ = 8.03 – 7.96 (m, 4H, C*H*$_{\text{Ph},ortho}$), 7.32 – 7.26 (m, 6H, C*H*$_{\text{Ph},meta,para}$) ppm.

^{13}C{^{1}H}-NMR (101 MHz, THF-d_8): δ = 140.39 (d, $^1J_{\text{CP}}$ = 97.5 Hz, *C*$_{\text{Ph},ipso}$), 140.18 (d, $^2J_{\text{CP}}$ = 36.0 Hz, PC*C*S), 131.49 (d, $^2J_{\text{CP}}$ = 12.1 Hz, *C*H$_{\text{Ph},ortho}$), 130.65 (d, $^4J_{\text{CP}}$ = 2.7 Hz, *C*H$_{\text{Ph},para}$),128.45 (d, $^3J_{\text{CP}}$ = 13.1 Hz, *C*H$_{\text{Ph},meta}$), 51.65 (d, $^1J_{\text{CP}}$ = 189.8 Hz, P*C*CS) ppm.

With crown ether

^{31}P{^{1}H}-NMR (162 MHz, THF-d_8): δ = 10.46 (s, *P*Ph$_2$S) ppm.

^{1}H-NMR (400 MHz, THF-d_8): δ = 8.07 – 8.01 (m, 4H, C*H*$_{\text{Ph},ortho}$), 7.26 – 7.23 (m, 6H, C*H*$_{\text{Ph},meta,para}$), 3.59 (s, 32H, C*H*$_{2,\text{crown}}$+ residual THF) ppm.

^{13}C{^{1}H}-NMR (101 MHz, THF-d_8): δ = 142.51 (d, $^1J_{\text{CP}}$ = 95.6 Hz, *C*$_{\text{Ph},ipso}$), 139.91 (d, $^2J_{\text{CP}}$ = 36.7 Hz, PC*C*S), 131.77 (d, $^2J_{\text{CP}}$ = 11.8 Hz, *C*H$_{\text{Ph},ortho}$), 129.79 (d, $^4J_{\text{CP}}$ = 2.9 Hz, *C*H$_{\text{Ph},para}$),127.99 (d, $^3J_{\text{CP}}$ = 12.9 Hz, *C*H$_{\text{Ph},meta}$), 71.22 (s, *C*$_{\text{crown}}$), 51.62 (d, $^1J_{\text{CP}}$ = 198.0 Hz, P*C*CS) ppm.

FT-IR (ATR, cm^{-1}): 2888.0 (bw), 2023.5 (s, CCS asym. stretching), 1956.7 (s, CCS sym. stretching), 1653.7 (bw), 1617.1 (w), 1435.4 (m), 1349.9 (s), 1096.5 (s), 998.1 (w), 959.3 (s), 835.1 (m), 639.1 (s), 530.6 (s), 500.5 (s).

Anal. Calcd for $C_{26}H_{34}KO_6PS_2$: C,54.15; H, 5.94; S, 11.12. Found: C, 53.91; H, 5.81; S, 10.25.

1.6. Synthesis of compounds 2^{Ts}

<u>Pathway 1</u>

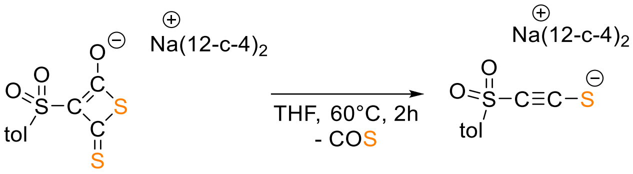

3^{Ts} 2^{Ts}

3^{Ts} (30 mg, 0.0464 mmol) was dissolved in 3 mL of THF. The Argon atmosphere was removed by freeze-pump-thaw and the solution was kept under static vacuum. Then, the solution was heated at 60°C during 2h. Volatiles were removed under vacuum and red solid was dissolved in 3 mL of THF and crystallized by pentane diffusion to obtain $\mathbf{2^{Ts}}$ as red crystals (22 mg, 0.0375 mmol, 81%).

^{1}H-NMR (400 MHz, CD_2Cl_2): δ = 7.76 (d, $^3J_{HH}$ = 8.4 Hz, 2H, C$H_{tol,ortho}$), 7.23 (d, $^3J_{HH}$ = 8.3 Hz, 2H, C$H_{tol,meta}$), 3.66 (s, 32H, C$H_{2,crown}$+ residual THF), 2.38 (s, 3H, CH_3) ppm.

^{13}C{^{1}H}-NMR (101 MHz, CD_2Cl_2): δ = 145.8 (s, C*C*S), 141.7 (s, $C_{tol,para}$), 136.1 (s, $C_{tol,ipso}$), 129.3 (s, $CH_{tol,meta}$), 125.8 (s, $CH_{tol,ortho}$), 66.4 (s, C_{crown}), 62.7 (s, *C*–SO_2tol), 21.5 (s, *C*H_3) ppm.

FT-IR (ATR, cm^{-1}): 2957.7 (bw), 2913.9 (w), 2869.4 (w), 2018.4 (s, CCS asym. stretching), 1956.7 (s, CCS sym. stretching), 1749.2 (bw), 1594.8 (bw), 1244.4 (s), 1131.6 (s), 1020.4 (s), 914.8 (s), 849.5 (s), 797.8 (w), 659.2 (s), 527.8 (s), 476.8 (w).

Our repeated attempts on obtaining satisfactory elemental analysis data were unsuccessful.

1.7. Synthesis of compounds 7

Synthesis of compounds 7a

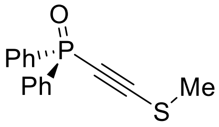

$\mathbf{2^{PO}}$ (35 mg, 0.118 mmol) was dissolved in 2mL THF. To the resulting solution, 7.4 µL (0.118 mmol) iodomethane was added and stirred overnight. The volatiles were removed *in vacuo* and the residue was extracted with 3 mL toluene. Filtration and subsequent removal of the volatiles in vacuo yielded **7a** as a brown oil. (29 mg, 0.106 mmol, 90 %).

^{31}P{^{1}H}-NMR (162 MHz, THF-d_8): δ = 2.49 (s, *P*Ph_2O) ppm.

^{1}H-NMR (400 MHz, THF-d_8): δ = 7.86 – 7.79 (m, 4H, C$H_{Ph,ortho}$), 7.53 – 7.43 (m, 6H, C$H_{Ph,meta,para}$), 2.52 (s, 3H, CH_3) ppm.

$^{13}C\{^1H\}$-NMR (101 MHz, THF-d_8): δ = 135.65 (d, $^1J_{CP}$ = 120.7 Hz, $C_{Ph,ipso}$), 132.62 (d, $^4J_{CP}$ = 3.0 Hz, $CH_{Ph,para}$), 131.67 (d, $^2J_{CP}$ = 10.7 Hz, $CH_{Ph,ortho}$), 129.34 (d, $^3J_{CP}$ = 13.1 Hz, $CH_{Ph,meta}$), 100.94 (d, $^2J_{CP}$ = 26.2 Hz, PC*C*S), 89.39 (d, $^1J_{CP}$ = 161.2 Hz, P*C*CS), 18.70 (d, $^4J_{CP}$ = 1.9 Hz, CH_3) ppm.

FT-IR (ATR, cm^{-1}): 3053.9 (bw), 2982.1 (bw), 2105.3 (s, C-C triple bond stretching), 1604.1(bw), 1436.1 (s), 1258.8 (s), 1118.0 (s), 1101.5 (s), 995.9 (w), 897.6 (s), 718.8 (s), 640.5 (w), 550.0 (s).

HRMS-LIFDI (m/z): $[M]^{\cdot+}$ calcd for $C_{15}H_{13}OPS$, 272.0425; found, 272.0426.

Synthesis of compounds 7b

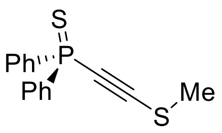

$\mathbf{2^{PS}}$ (40 mg, 0.128 mmol) was dissolved in 2mL THF. To the resulting solution, 8.1 µL (0.128 mmol) iodomethane was added and stirred overnight. The volatiles were removed *in vacuo* and the residue was extracted with 3 mL toluene. Filtration and subsequent removal of the volatiles in vacuo yielded **7b** as a brown oil. (30 mg, 0.104 mmol, 81 %).

$^{31}P\{^1H\}$-NMR (162 MHz, C_6D_6): δ = 19.36 (s, *P*Ph_2S) ppm.

^1H-NMR (400 MHz, C_6D_6): δ = 8.09 – 8.00 (m, 4H, $CH_{Ph,ortho}$), 6.99 – 6.97 (m, 6H, $CH_{Ph,meta,para}$), 1.48 (s, 3H, CH_3) ppm.

$^{13}C\{^1H\}$-NMR (101 MHz, C_6D_6): δ = 135.16 (d, $^1J_{CP}$ = 98.3 Hz, $C_{Ph,ipso}$), 131.60 (d, $^4J_{CP}$ = 3.2 Hz, $CH_{Ph,para}$), 131.27 (d, $^2J_{CP}$ = 12.1 Hz, $CH_{Ph,ortho}$), 128.73 (d, $^3J_{CP}$ = 13.6 Hz, $CH_{Ph,meta}$), 101.52 (d, $^2J_{CP}$ = 23.6 Hz, PC*C*S), 87.34 (d, $^1J_{CP}$ = 146.9 Hz, P*C*CS), 17.73 (d, $^4J_{CP}$ = 2.1 Hz, CH_3) ppm.

FT-IR (ATR, cm^{-1}): 3051.7 (bw), 2962.7 (bw), 2101.03 (s, C-C triple bond stretching), 1956.7 (bw), 1647.2 (bw),1479.2 (m), 1435.4 (s), 1098.6 (s), 1069.2 (w), 899.0 (s), 796.3 (w), 710.9 (s), 521.3 (s), 501.2 (s).

HRMS-LIFDI (m/z): $[M]^{\cdot+}$ calcd for $C_{15}H_{13}PS_2$, 288.0196; found, 288.0201.

1.8. Synthesis of compounds 8

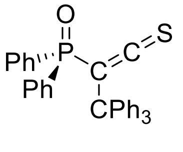

$\mathbf{2^{PO}}$ (50mg, 0.169 mmol) was dissolved in 4mL THF. To the resulting solution, 51.7mg (0.186 mmol) trityl chloride was added and stirred for overnight. All the volatiles were removed in *vacuo* and the brown oil obtained was dissolved in 4ml hexane. After filtration, the volatiles were removed from the filtrate to obtain the mixture of desired product **8** and trityl chloride as light brown colour powder. Slow evaporation of a benzene solution of this brown powder, yielded **8** as light brown colour crystals along with colourless crystals of trityl chloride. The single crystals of **8** were handpicked from mixture of crystals and used for X-ray diffraction analysis.

$^{31}P\{^1H\}$-NMR (162 MHz, C_6D_6): δ = 24.67 (s, *P*Ph_2O) ppm.

^1H-NMR (400 MHz, C_6D_6): δ = {peaks at 7.37 – 7.35 (m, 10H) and 7.03 – 6.96 (m, 15H) ppm corresponds to trityl chloride} 7.81 – 7.72 (m, 4H, $CH_{P\text{-}Ph,ortho}$), 7.70 – 7.68 (m, 6H, $CH_{C\text{-}Ph,ortho}$), 7.03 – 6.96 (m, 15H, $CH_{P\text{-}Ph,meta,para}$ + $CH_{C\text{-}Ph,meta,para}$) ppm.

$^{13}C\{^1H\}$-NMR (101 MHz, C_6D_6): δ = {peaks at 145.90, 130.19, 128.02, 127.96 and 81.96 ppm corresponds to trityl chloride} 145.03 (d, $^3J_{CP}$ = 3.1 Hz, $C_{C\text{-}Ph,ipso}$), 142.37 (s, PC*C*S), 135.20 (d, $^1J_{CP}$ = 109.1 Hz, $C_{P\text{-}Ph,ipso}$), 132.21 (d, $^2J_{CP}$ = 9.4 Hz, $CH_{P\text{-}Ph,ortho}$), 131.53 (d, $^4J_{CP}$ = 3.0 Hz, $CH_{P\text{-}Ph,para}$), 131.32 (s, $CH_{C\text{-}Ph,ortho}$), 128.07 (d, $^3J_{CP}$ = 12.2 Hz, $CH_{P\text{-}Ph,meta}$), 127.74 (s, $CH_{C\text{-}Ph,meta}$), 126.96 (s, $CH_{C\text{-}Ph,para}$), 86.30 (d, $^1J_{CP}$ = 87.0 Hz, P*C*CS), 63.08 (d, $^2J_{CP}$ = 2.9 Hz,*C*Ph_3) ppm.

FT-IR (ATR, cm^{-1}): 3055.32 (bw), 2963.41 (bw), 1711.12 (s, CCS stretching), 1596,2 (bw), 1490.0 (m), 1436.11 (s), 1260.2(s), 975.1 (m), 915.5 (m), 747,5 (s), 691.5 (s), 606.0 (w), 538.5 (s), 509.8 (s).

HRMS-LIFDI (m/z): $[M]^{\bullet +}$ calcd for $C_{33}H_{25}OPS$, 500.1364; found, 500.1366.

1.9. Synthesis of compounds 9

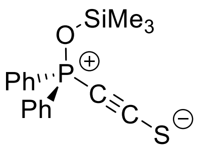

2^{PO} (40mg, 0.135 mmol) was dissolved in 2mL THF. To the resulting solution, 17.4 µL (0.135 mmol) chlorotrimethylsilane was added and stirred for 3 h. After filtration, filtrate was collected in a vial. Vapour diffusion of pentane into filtrate at − 30 °C yielded the product as yellow block shape crystals (37 mg, 0.112 mmol, 83%). The single crystals were used for X-ray diffraction analysis.

$^{31}P\{^1H\}$-NMR (162 MHz, C_6D_6): δ = 7.65 (s, *P*Ph_2O) ppm.

^1H-NMR (400 MHz, C_6D_6): δ = 7.73 – 7.66 (m, 4H, $CH_{Ph,ortho}$), 7.02 – 6.90 (m, 6H, $CH_{Ph,meta,para}$), 0.13 (s, 9H, CH_3) ppm.

$^{13}C\{^1H\}$-NMR (101 MHz, C_6D_6): δ = 158.26 (d, $^2J_{CP}$ = 48.2 Hz, PC*C*S), 132.68 (d, $^4J_{CP}$ = 3.1 Hz, $CH_{Ph,para}$), 131.56 (d, $^1J_{CP}$ = 132.68 Hz, $C_{Ph,ipso}$), 131.18 (d, $^2J_{CP}$ = 12.6 Hz, $CH_{Ph,ortho}$), 128.97 (d, $^3J_{CP}$ = 14.3 Hz, $CH_{Ph,meta}$), 43.46 (d, $^1J_{CP}$ = 235.2 Hz, P*C*CS), 0.82 (s, CH_3) ppm.

FT-IR (ATR, cm^{-1}): 3053.9 (bw), 2959.8 (bw), 1959.6 (s, CCS stretching), 1706.8 (bw), 1495.7 (m), 1436.11 (s), 1334.15 (bw), 1100.78 (s), 995.9 (w), 843.7 (s), 807.5 (m), 640.6 (w), 522.0 (s).

HRMS-LIFDI (m/z): $[M]^{\bullet +}$ calcd for $C_{37}H_{19}OPSSi$, 330.0664; found, 330.0668.

1.10. Synthesis of compounds 10

Synthesis of compounds 10a

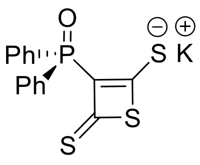

2^{PO} (40mg, 0.135 mmol) was dissolved in 2mL THF. To the resulting solution, 8.2 µL (0.135 mmol) CS_2 was added and stirred for overnight. After removal of solvent, residue obtained was washed with pentane (2 x 5 mL). After drying in vacuo, **10a** was obtained as a light brown colour solid (40 mg, 0.107 mmol, 80%).

$^{31}P\{^1H\}$-NMR (162 MHz, THF-d_8): δ = 13.11 (s, *P*Ph_2O) ppm.

^1H-NMR (400 MHz, THF-d_8): δ = 7.80 – 7.75 (m, 4H, C$H_{Ph,ortho}$), 7.42 – 7.27 (m, 6H, C$H_{Ph,meta,para}$) ppm.

$^{13}C\{^1H\}$-NMR (101 MHz, THF-d_8): δ = 206.78 (d, $^2J_{CP}$ = 10.0 Hz, PC*C*S), 136.16 (d, $^1J_{CP}$ = 107.0 Hz, $C_{Ph,ipso}$), 132.94 (d, $^2J_{CP}$ = 10.6 Hz, $CH_{Ph,ortho}$), 131.53 (d, $^4J_{CP}$ = 2.8 Hz, $CH_{Ph,para}$), 128.43 (d, $^3J_{CP}$ = 12.2 Hz, $CH_{Ph,meta}$), not observed (P*C*CS) ppm.

FT-IR (ATR, cm^{-1}): 2942.6 (bw), 2883.0 (bw), 1434.0 (m) 1349.2 (s), 1246.5 (s), 1100.8 (s), 959.3 (s), 855.2 (m), 784.1 (w), 748.9 (m), 723.1 (s), 687.9 (s), 533.5 (s), 519.9 (s), 488.2 (m).

Our repeated attempts on obtaining satisfactory elemental analysis data were unsuccessful.

Synthesis of compounds 10b

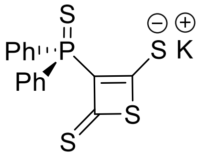

2^{PS} (40mg, 0.128 mmol) and 18-crown-6 (33.8 mg, 0.128mmol) were dissolved in 2mL THF. To the resulting solution, 8.5 µL (0.141 mmol) CS_2 was added and stirred for overnight. After removal of solvent, residue obtained was washed with pentane (2 x 5 mL). After drying in vacuo, **10b(18-c-6)** was obtained as a light brown colour solid (65 mg, 0.010 mmol, 78%).

$^{31}P\{^1H\}$-NMR (162 MHz, CD_2Cl_2): δ = 20.56 (s, *P*Ph_2S) ppm.

^1H-NMR (400 MHz, CD_2Cl_2): δ = 7.95 – 7.89 (m, 4H, C$H_{Ph,ortho}$), 7.42 – 7.34 (m, 6H, C$H_{Ph,meta,para}$), 3.61 (s, 32H, C$H_{2,crown}$)ppm.

$^{13}C\{^1H\}$-NMR (101 MHz, CD_2Cl_2): δ = 203.92(d, $^2J_{CP}$ = 9.3 Hz, PC*C*S), 134.54 (d, $^1J_{CP}$ = 87.2 Hz, $C_{Ph,ipso}$), 132.64 (d, $^2J_{CP}$ = 11.4 Hz, $CH_{Ph,ortho}$), 130.82 (d, $^4J_{CP}$ = 3.1 Hz, $CH_{Ph,para}$), 128.04 (d, $^3J_{CP}$ = 12.6 Hz, $CH_{Ph,meta}$), 123.45 (d, $^1J_{CP}$ = 91.5 Hz, P*C*CS) ppm.

FT-IR (ATR, cm^{-1}): 2895.2 (bw), 2824.1 (bw), 1470.6 (w), 1436.1 (m), 1349.9 (m), 1240.1 (s), 1096.5 (s), 960.0 (s), 835.1 (m), 690.8 (m), 639.8 (s), 545.0 (w), 516.3 (s), 485.4 (w).

Our repeated attempts on obtaining satisfactory elemental analysis data were unsuccessful.

1.11. Synthesis of compounds 11

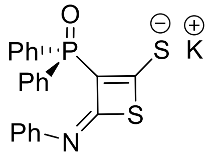

40 mg (0.135 mmol) compound **2^{PO}**and 18.6 mg (0.135 mmol) phenyl isothiocyanate were dissolved in 2 ml THF and stirred overnight. The solution obtained was concentrated to half it's volume and 5 mL pentane was added, which resulted in the precipitation of a solid. The solid was filtered and washed with pentane (2 x 5 mL). After drying *in vacuo*, **11** was obtained as a light brown colour solid (45 mg, 0.104 mmol, 77%).

^{31}P{^{1}H}-NMR (162 MHz, THF-d_8): δ = 13.55 (s, *P*Ph$_2$O) ppm.

^{1}H-NMR (400 MHz, THF-d_8): δ = 7.98 – 7.92 (m, 4H, PC$H_{\text{Ph},ortho}$), 7.42 – 7.31 (m, 6H, PC$H_{\text{Ph},meta,para}$), 7.16 (t, $^{3}J_{HH}$ = 7.8 Hz, 2H, NC$H_{\text{Ph},ortho}$), 6.97 – 6.91 (m, 3H, NC$H_{\text{Ph},meta,para}$), ppm.

^{13}C{^{1}H}-NMR (101 MHz, THF-d_8): δ = 198.06 (d, $^{2}J_{CP}$ = 8.6 Hz, PC*C*S), 156.01 (d, $^{2}J_{CP}$ = 10.3 Hz, PC*C*N), 150.87 (s, N$C_{\text{Ph},ipso}$), 137.44 (d, $^{1}J_{CP}$ = 105.9 Hz, P$C_{\text{Ph},ipso}$), 132.77 (d, $^{2}J_{CP}$ = 10.8 Hz, P$CH_{\text{Ph},ortho}$), 131.31 (d, $^{4}J_{CP}$ = 2.9 Hz, P$CH_{\text{Ph},para}$),129.21 (s, N$CH_{\text{Ph,ortho}}$), 128.35 (d, $^{3}J_{CP}$ = 12.1 Hz, P$CH_{\text{Ph},meta}$), 124.10 (s, N$CH_{\text{Ph,para}}$), 122.63 (s, N$CH_{\text{Ph,meta}}$), 111.35 (d, $^{1}J_{CP}$ = 113.8 Hz, P*C*CS) ppm.

FT-IR (ATR, cm^{-1}): 3050.3 (bw), 2884.4 (bw), 1665.2 (m), 1587.6 (s), 1485.7 (w), 1349.9 (s), 1100.8 (s), 1027.5 (w), 960.0 (s), 836.5 (m), 693.6 (s), 619.7 (w), 534.9 (s), 515.4 (s)

Anal. Calcd for $C_{21}H_{15}KNOPS_2$: C, 58.45; H, 3.50; N, 3.25; S, 14.86. Found: C, 58.15; H, 3.45; N, 3.13; S, 13.53.

1.12. Synthesis of compound 12

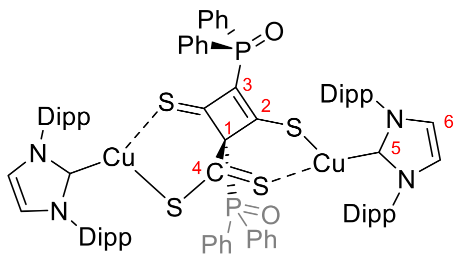

2^{PO} (25mg, 0.084 mmol) and IPrCuCl (41.1mg, 0.084 mmol) were dissolved in 4mL THF. To the resulting solution, 2.6 µL (0.042 mmol) CS_2 was added and stirred for overnight. All the volatiles were removed in *vacuo* and the brown oil obtained was dissolved in 4ml benzene. After filtration, the volatiles were removed from the filtrate. The resulting solid was washed with hexane (2 x 3 mL) to obtain the desired product **12** (50 mg, 0.033 mmol, 79%).as brown colour powder. Single crystals suitable for X-ray diffraction analysis were grown by slow vapor diffusion of pentane into a saturated solution of **12** in benzene at room temperature.

^{31}P{^{1}H}-NMR (162 MHz, C_6D_6): δ = 30.56 (s, *P*Ph$_2$O), 7.75 (s, *P*Ph$_2$O) ppm.

^{1}H-NMR (400 MHz, C_6D_6): δ = 7.93 – 7.88 (m, 4H, C$H_{\text{Ph},ortho}$), 7.54 – 7.49 (m, 4H, C$H_{\text{Ph},ortho}$), 7.22 – 7.18 (m, 4H, C$H_{\text{Dipp},para}$ + m, 4H, C$H_{\text{Ph},para}$), 7.11 – 7.02 (m, 8H, C$H_{\text{Ph},meta}$ + m, 8H,

C$H_{Dipp,meta}$), 6.43 (s, 4H, C6H), 2.77 (septet, 8H, $^3J_{HH}$ = 6.9 Hz, CH(CH_3)$_2$), 1.30 – 1.27 (m, 24H, CH_3), 1.07 – 1.03 (m, 24H, CH_3)ppm.

^{13}C{^{1}H}-NMR (101 MHz, C_6D_6): δ = 239.84 (s, *C*4), 206.03 (s, *C*2), 189.03 (s, *C*5), 146.34 (d, $^1J_{CP}$ = 56.9 Hz, $C_{Ph,ipso}$), 145.86 (s, $CH_{Dipp,ortho}$), 145.79 (s, $CH_{Dipp,ortho}$), 136.47(s, $C_{Dipp,ipso}$), 135.27 (d, $^1J_{CP}$ = 105.6 Hz, $C_{Ph,ipso}$), 133.55 (d, $^2J_{CP}$ = 9.9 Hz, $CH_{Ph,ortho}$), 132.39 (d, $^2J_{CP}$ = 10.1 Hz, $CH_{Ph,ortho}$), 131.32 (s, $CH_{Ph,para}$), 130.37 (s, $CH_{Ph,para}$), 129.95 (s, $CH_{Dipp,para}$), 127.53 (d, $^3J_{CP}$ = 12.3 Hz, $CH_{Ph,meta}$), 124.35 (d, $^3J_{CP}$ = 9.6 Hz, $CH_{Ph,meta}$), 124.30 (s, $CH_{Dipp,meta}$), 124.12 (s, $CH_{Dipp,meta}$), 122.29 (s, *C*6), 88.09 (dd, $^1J_{CP}$ = 55.9 Hz, $^3J_{CP}$ = 24.7 Hz, *C*3), 28.97 (s, *C*H(CH_3)$_2$), 24.93 (s, *C*H_3), 24.88 (s, *C*H_3), 24.12 (s, *C*H_3), 23.97 (s, *C*H_3), not observed (*C*1) ppm.

FT-IR (ATR, cm^{-1}): 3053.8 (bw), 2926.7 (bw), 1603.4 (w), 1436.8 (m), 1384.38 (w), 1262.3 (s), 1156.8 (m), 1027.5 (m), 800.6 (m), 690.0 (s), 631.2 (m), 539.2(s), 502.6 (s), 450.9 (w).

HRMS-LIFDI (m/z): [M] $^{\bullet+}$ calcd for $C_{83}H_{92}Cu_2N_4O_2P_2S_4$, 1492.4170; found, 1492.4159.

2. NMR and IR spectra

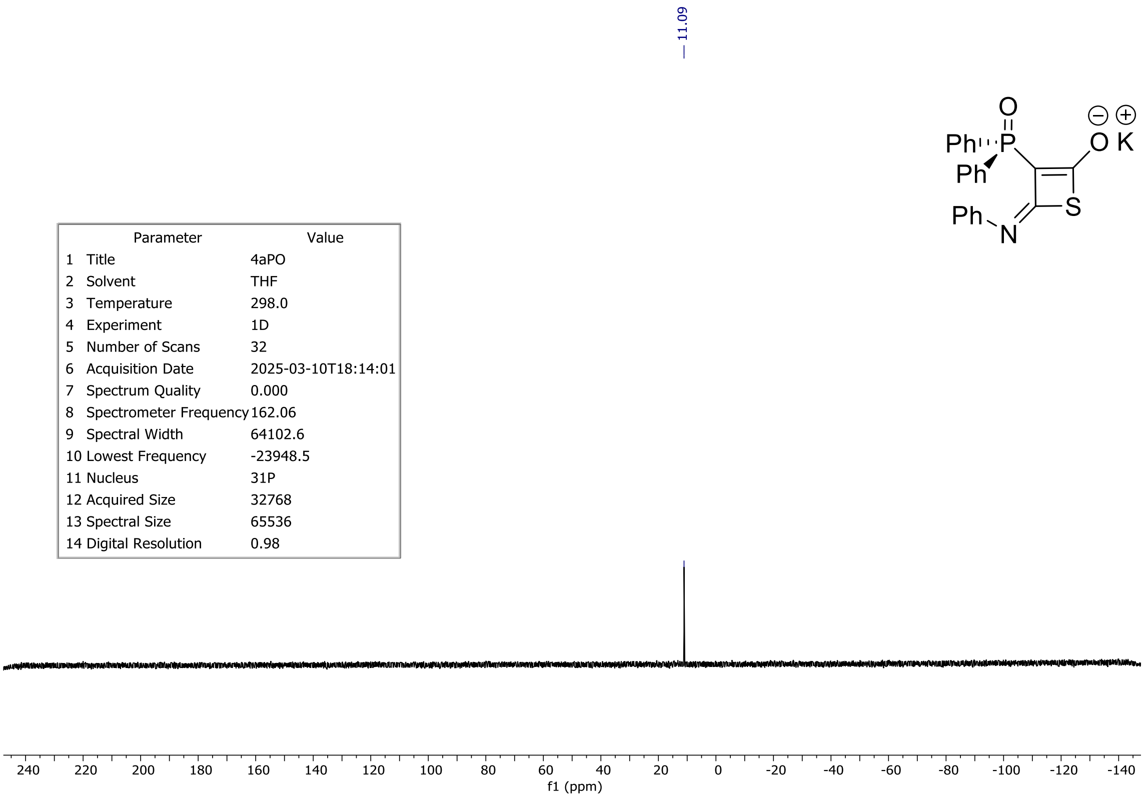

Figure S5 $^{31}P\{^1H\}$ NMR spectrum of compound **4a^{PO}** in THF-d$_8$. **$^{31}P\{^1H\}$-NMR** (162 MHz, THF-d8): δ = 11.09 (s, *P*Ph$_2$O) ppm.

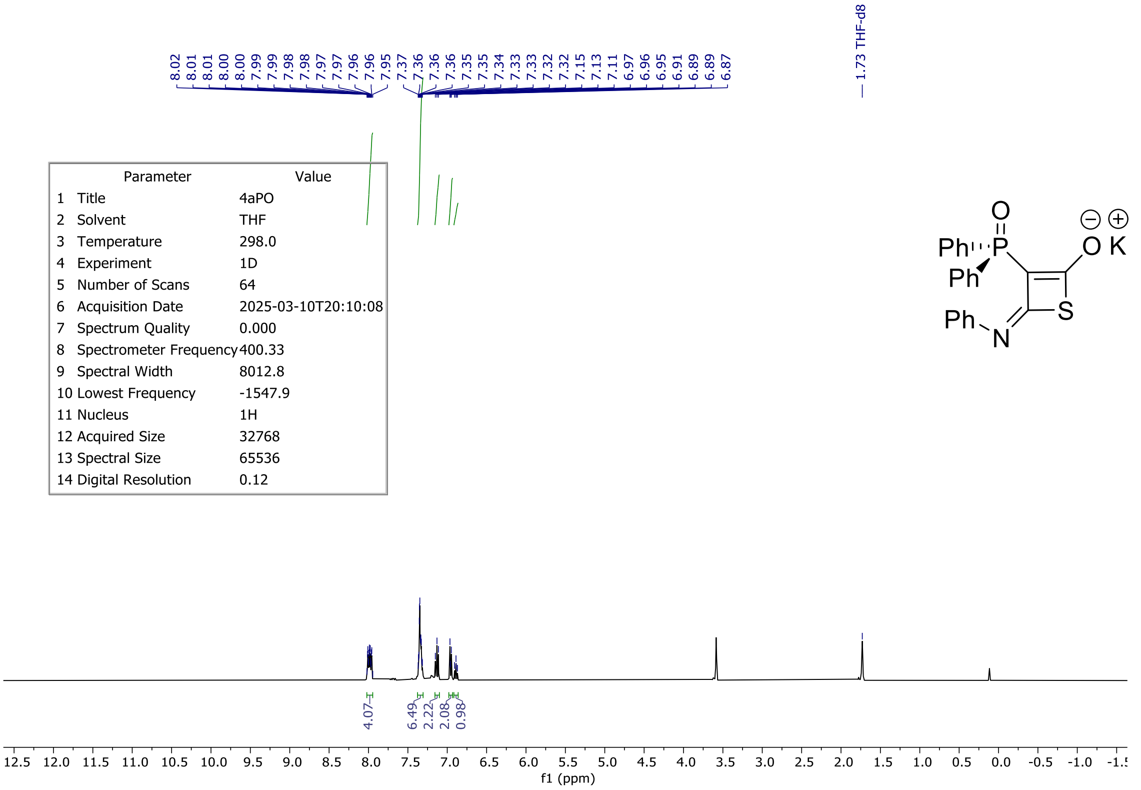

Figure S6 ^1H NMR spectrum of compound **4a^{PO}** in THF-d$_8$. **^1H-NMR** (400 MHz, THF-d_8): δ = 8.02 – 7.95 (m, 4H, PC*H*$_{Ph,ortho}$), 7.37 – 7.32 (m, 6H, PC*H*$_{Ph,meta,para}$), 7.13 (t, $^3J_{HH}$ = 7.3 Hz, 2H, NC*H*$_{Ph,ortho}$), 6.96 (d, $^3J_{HH}$ = 7.0 Hz, 2H, NC*H*$_{Ph,meta}$), 6.89 (t, $^3J_{HH}$ = 7.3 Hz, 1H, NC*H*$_{Ph,para}$) ppm.

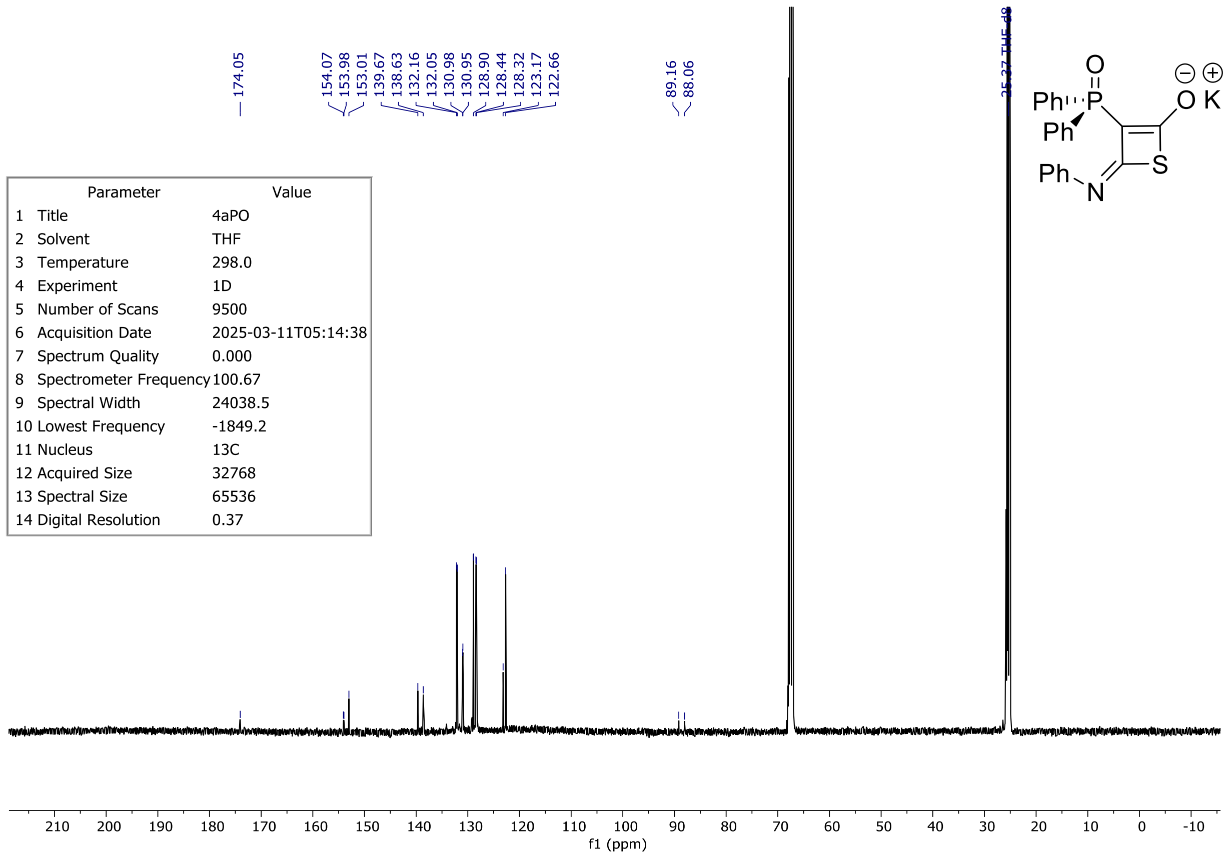

Parameter	Value
1 Title	4aPO
2 Solvent	THF
3 Temperature	298.0
4 Experiment	1D
5 Number of Scans	9500
6 Acquisition Date	2025-03-11T05:14:38
7 Spectrum Quality	0.000
8 Spectrometer Frequency	100.67
9 Spectral Width	24038.5
10 Lowest Frequency	-1849.2
11 Nucleus	13C
12 Acquired Size	32768
13 Spectral Size	65536
14 Digital Resolution	0.37

Figure S7 $^{13}C\{^{1}H\}$ NMR spectrum of compound **4a**$^{\mathbf{PO}}$ in THF-d_8. **^{13}C{^{1}H}-NMR** (101 MHz, THF-d_8): δ = 174.05(s, PC*C*O), 154.03 (d, $^{2}J_{CP}$ = 9.5 Hz, PC*C*N), 153.01 (s, N*C*$_{Ph,ipso}$), 139.15 (d, $^{1}J_{CP}$ = 104.6 Hz, P*C*$_{Ph,ipso}$), 132.10 (d, $^{2}J_{CP}$ = 11.0 Hz, P*C*H$_{Ph,ortho}$), 130.97 (d, $^{4}J_{CP}$ = 2.7 Hz, P*C*H$_{Ph,para}$),128.90 (s, N*C*H$_{Ph,ortho}$), 128.38 (d, $^{3}J_{CP}$ = 12.1 Hz, P*C*H$_{Ph,meta}$), 123.17 (s, N*C*H$_{Ph,para}$), 122.66 (s, N*C*H$_{Ph,meta}$), 88.61 (d, $^{1}J_{CP}$ = 110.4 Hz, P*C*CO) ppm.

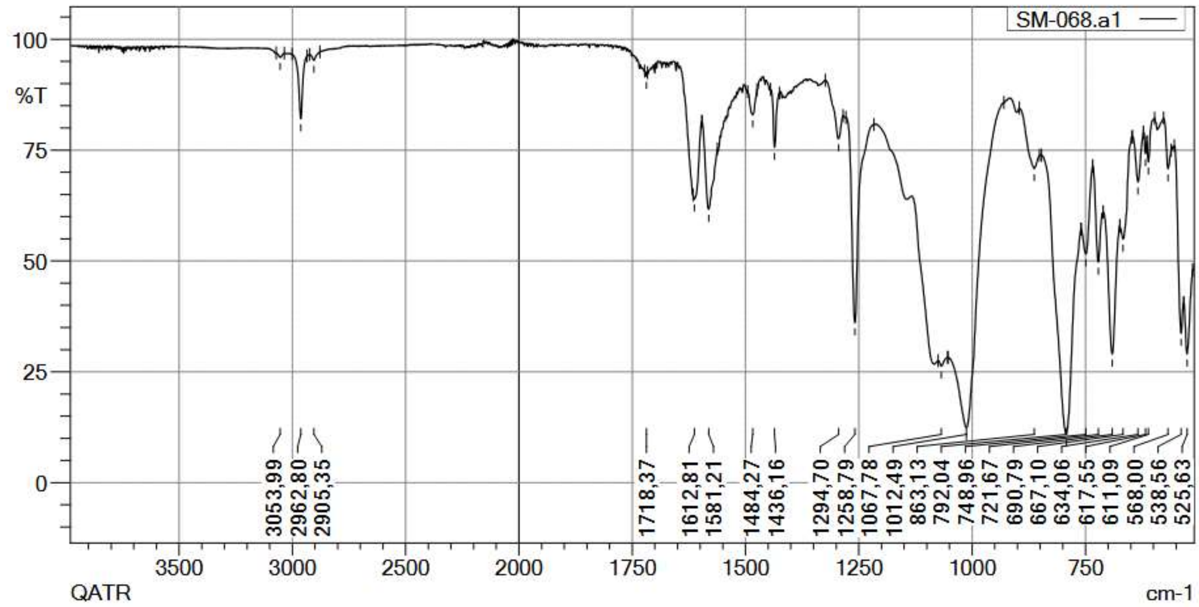

Figure S8 IR spectrum of compound **4a**$^{\mathbf{PO}}$ (solid state).

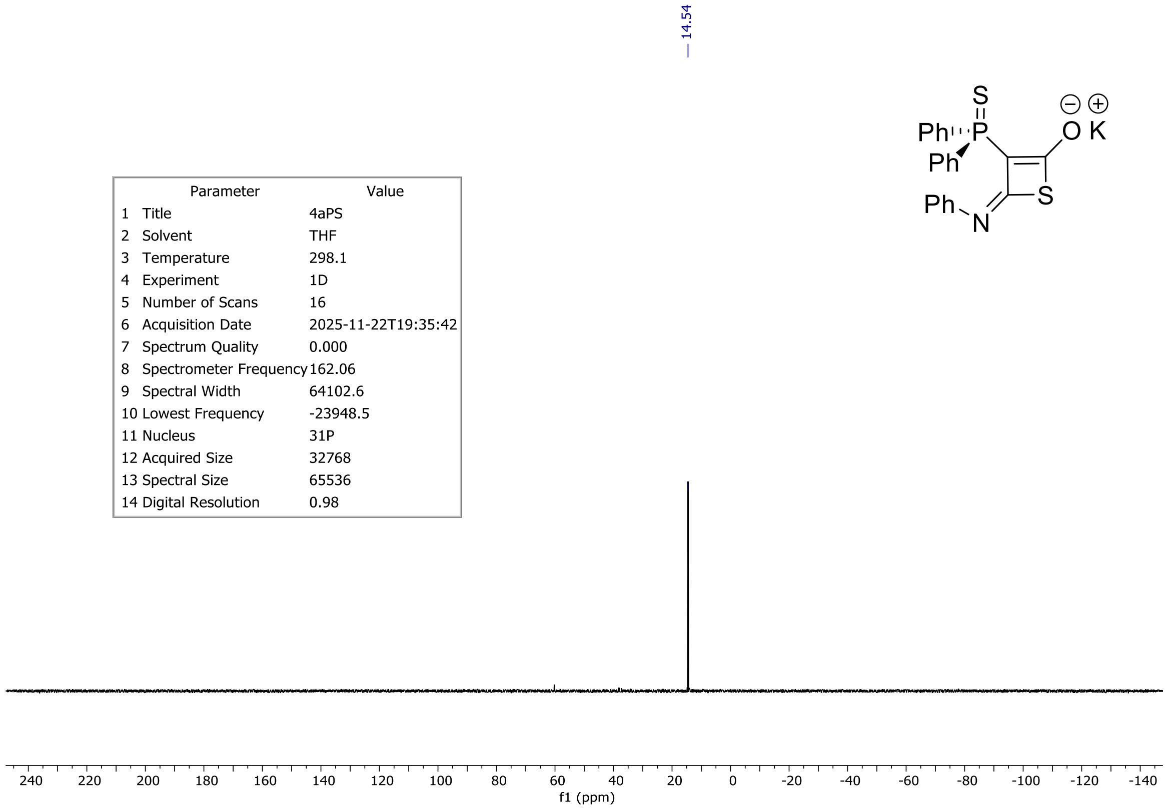

Figure S9 ^{31}P{^{1}H} NMR spectrum of compound **4a^{PS}** in THF-d$_8$. **^{31}P{^{1}H}-NMR** (162 MHz, THF-*d*$_8$): δ = 14.54 (s, *P*Ph$_2$S) ppm.

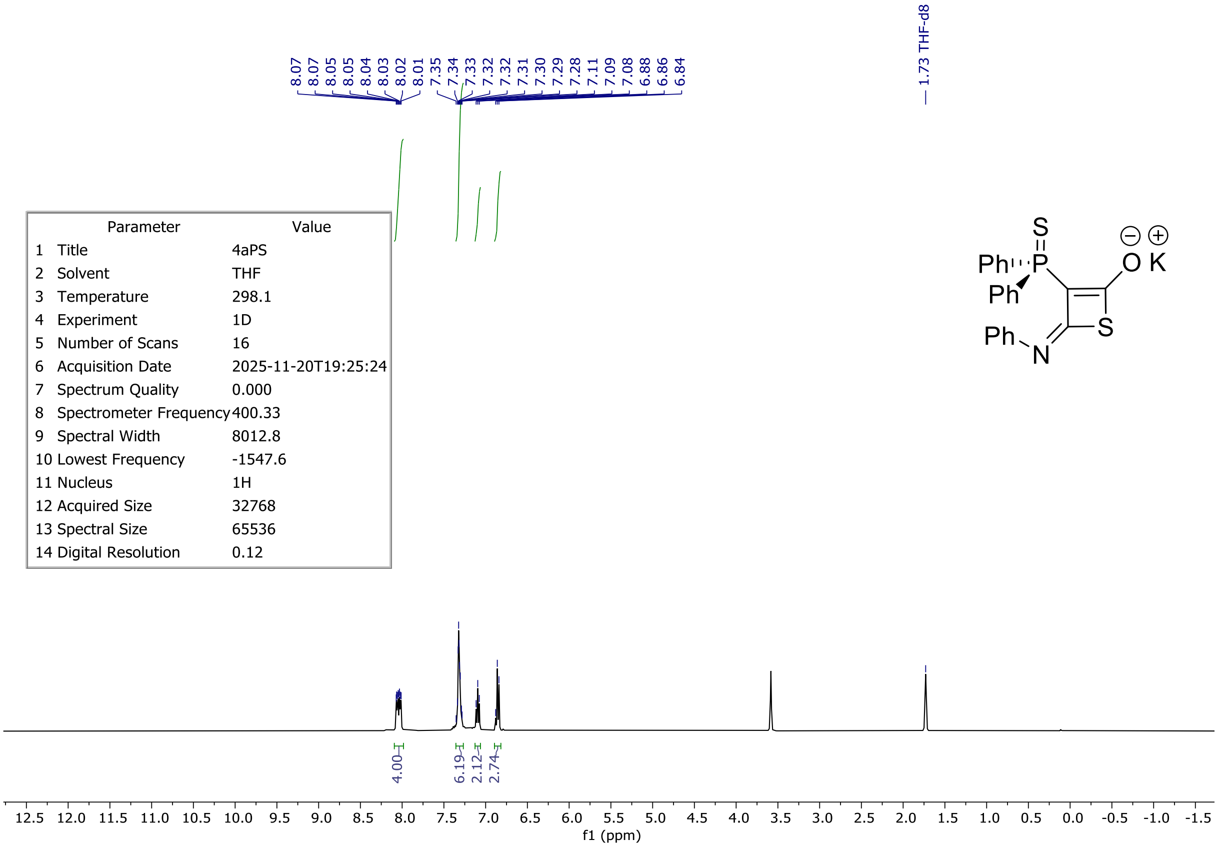

Figure S10 ^{1}H NMR spectrum of compound **4a^{PS}** in THF-d$_8$. **^{1}H-NMR** (400 MHz, THF-*d*$_8$): δ = 8.07 – 8.01 (m, 4H, PC*H*$_{Ph,ortho}$), 7.35 – 7.28 (m, 6H, PC*H*$_{Ph,meta,para}$), 7.10(t, $^{3}J_{HH}$ = 7.8 Hz, 2H, NC*H*$_{Ph,ortho}$), 6.88 – 6.84(m, 3H, NC*H*$_{Ph,meta,para}$) ppm.

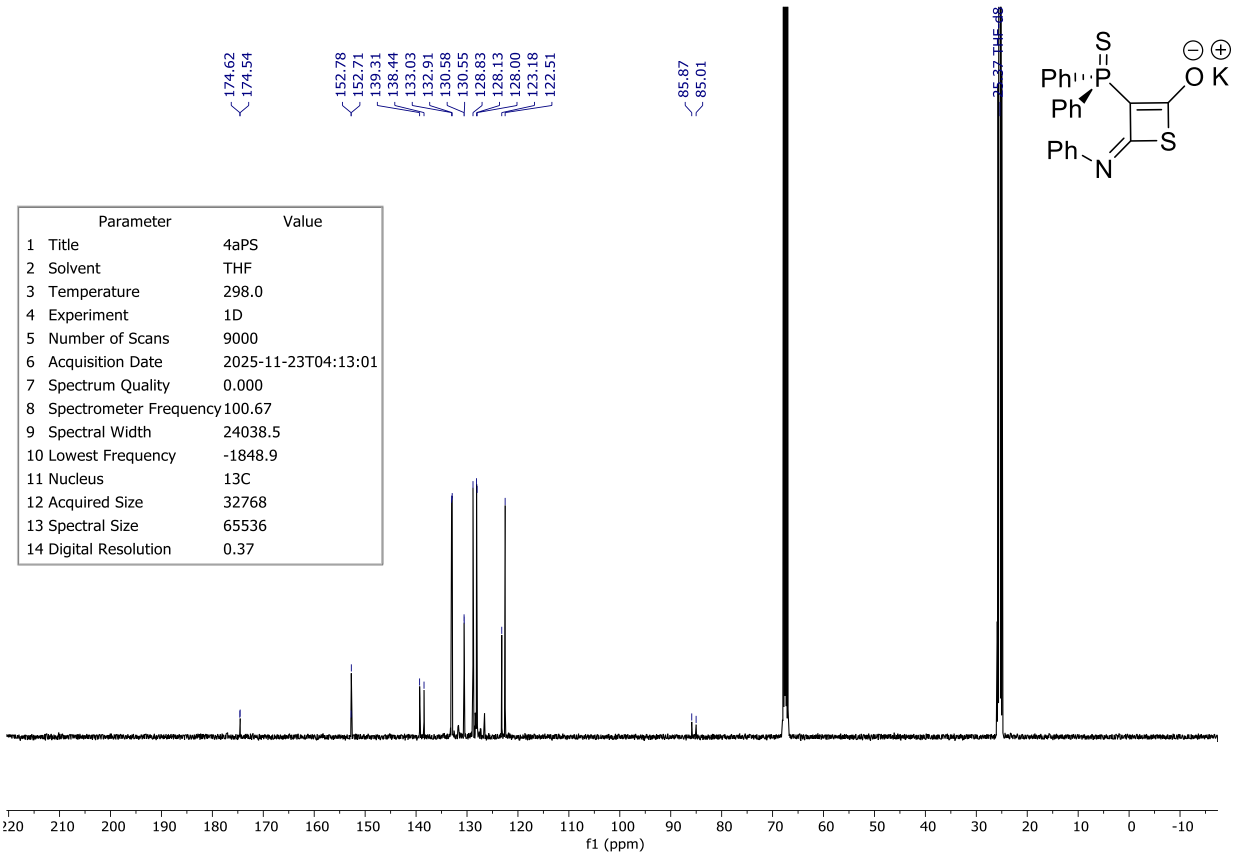

Figure S11 $^{13}C\{^1H\}$ NMR spectrum of compound **4a^PS^** in THF-d8. **$^{13}C\{^1H\}$-NMR** (101 MHz, THF-d_8): δ = 174.58(d, $^2J_{CP}$ = 8.0 Hz, PC*C*O), 152.75 (d, $^2J_{CP}$ = 6.5 Hz, PC*C*N), 152.71 (s, N*C*$_{Ph,ipso}$), 138.87 (d, $^1J_{CP}$ = 87.1 Hz, P*C*$_{Ph,ipso}$), 132.97 (d, $^2J_{CP}$ = 11.9 Hz, P*C*H$_{Ph,ortho}$), 130.56 (d, $^4J_{CP}$ = 3.1 Hz, P*C*H$_{Ph,para}$),128.83 (s, N*C*H$_{Ph,ortho}$), 128.06 (d, $^3J_{CP}$ = 12.7 Hz, P*C*H$_{Ph,meta}$), 123.18 (s, N*C*H$_{Ph,para}$), 122.51 (s, N*C*H$_{Ph,meta}$), 85.44 (d, $^1J_{CP}$ = 86.5 Hz, P*C*CO) ppm.

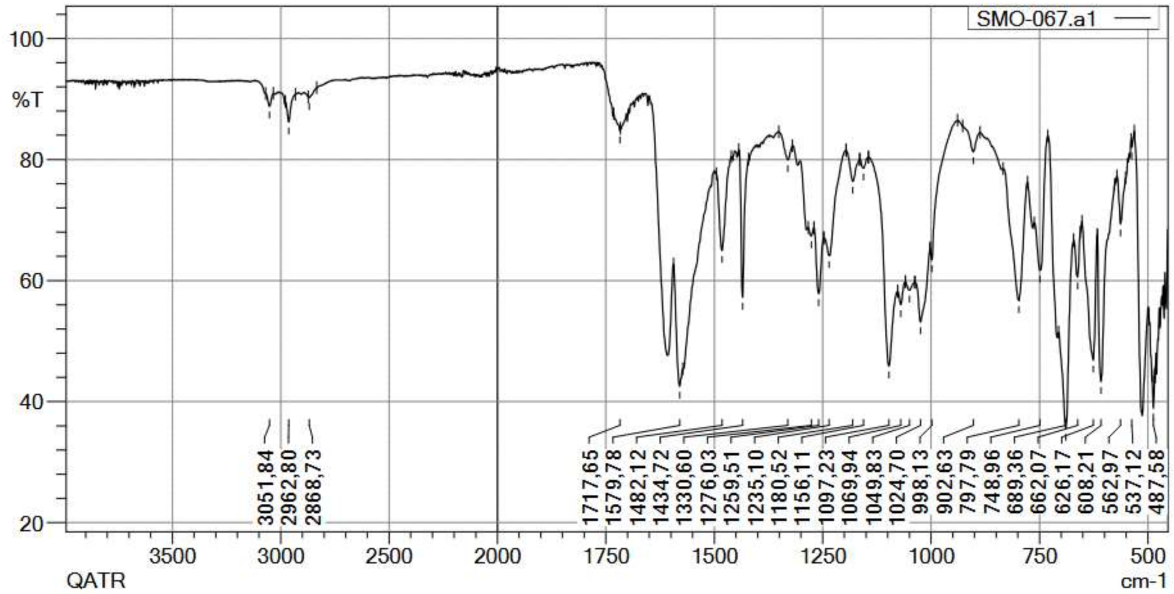

Figure S12 IR spectrum of compound **4a^PS^** (solid state).

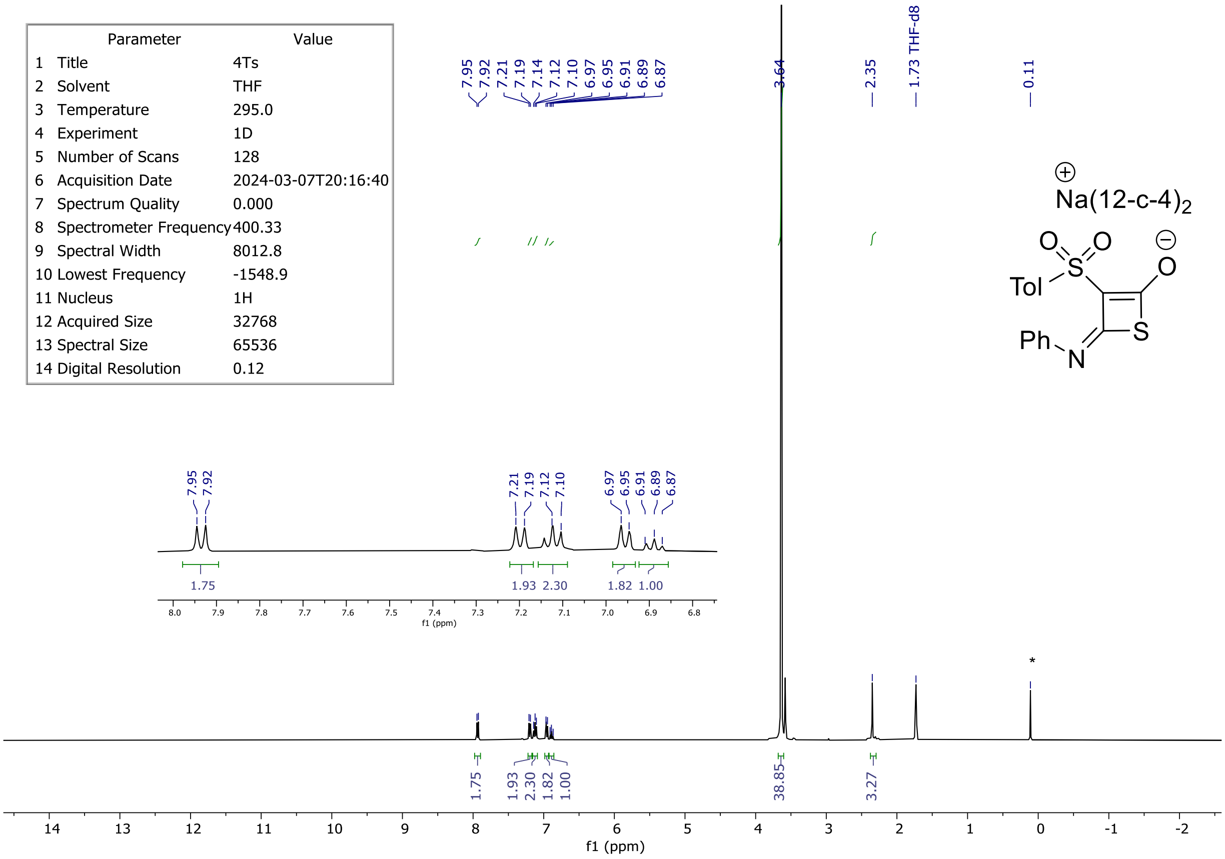

Figure S13 ^{1}H NMR spectrum of compound $\mathbf{4^{Ts}}$ in THF-d_8. **^{1}H-NMR** (400 MHz, THF-d_8): δ = 7.94 (d, $^{3}J_{HH}$ = 8.1 Hz, 2H, C$H_{tol,ortho}$), 7.20 (d, $^{3}J_{HH}$ = 8.0 Hz, 2H, C$H_{tol,meta}$), 7.15 – 7.10 (m, 2H, NC$H_{Ph,ortho}$), 6.96 (d, $^{3}J_{HH}$ = 7.4 Hz, 2H, NC$H_{Ph,meta}$), 6.89 (t, $^{3}J_{HH}$ = 7.3 Hz, 1H, NC$H_{Ph,para}$) 3.64 (s, 32H, C$H_{2,crown}$ + residual THF), 2.35 (s, 3H, CH_3) ppm. The peak at 0.11 ppm corresponds to silicone grease.

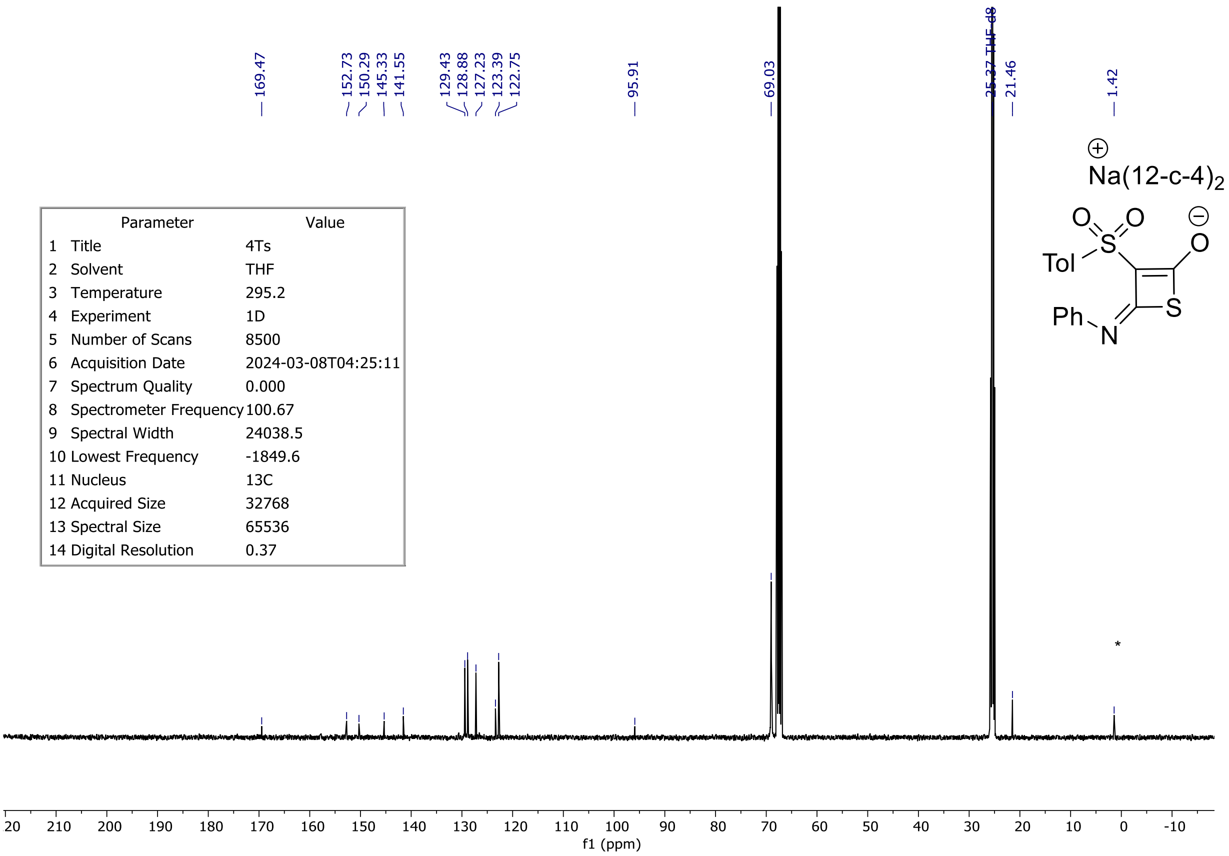

Figure S14 ^{13}C{^{1}H} NMR spectrum of compound **4^{Ts}** in THF-d$_8$. **^{13}C{^{1}H}-NMR** (101 MHz, THF-*d*$_8$): δ = 169.5 (s, *C*=O), 152.7 (s, N*C*$_{Ph,ipso}$), 150.3 (s, *C*=N), 145.3 (s, *C*$_{tol,para}$), 141.5 (s, *C*$_{tol,ipso}$), 129.4 (s, *C*H$_{tol,meta}$), 128.9 (s, N*C*H$_{Ph,ortho}$), 127.2 (s, *C*H$_{tol,ortho}$), 123.4 (s, N*C*$_{Ph,para}$), 122.8 (s, N*C*H$_{Ph,meta}$), 95.9 (s, *C*–SO$_2$tol), 69.0 (s, C$_{crown}$), 21.5 (s, *C*H$_3$) ppm. The peak at 1.42 ppm corresponds to silicone grease.

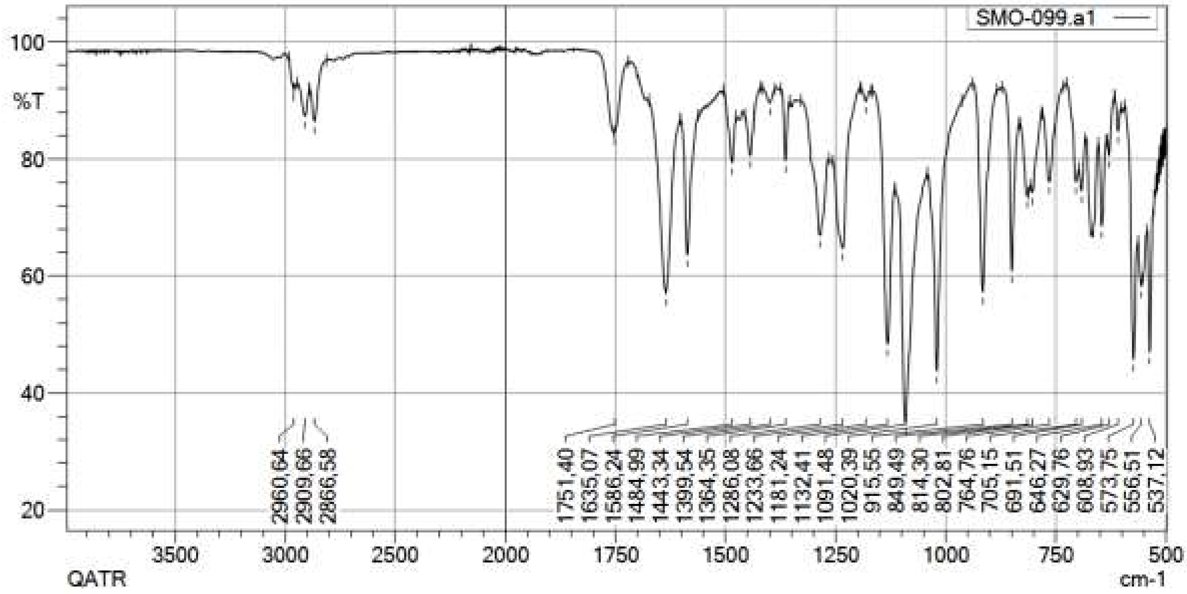

Figure S15 IR spectrum of compound **4^{Ts}** (solid state).

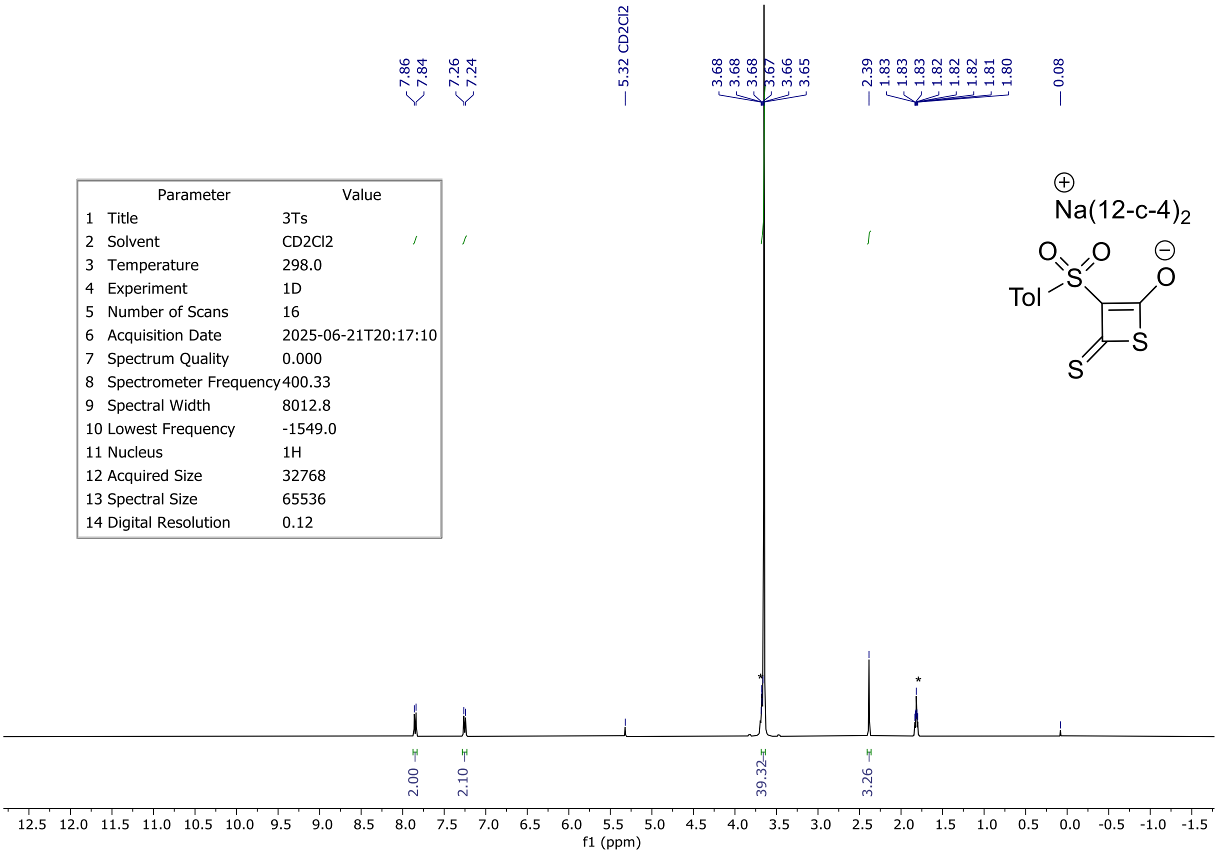

	Parameter	Value
1	Title	3Ts
2	Solvent	CD2Cl2
3	Temperature	298.0
4	Experiment	1D
5	Number of Scans	16
6	Acquisition Date	2025-06-21T20:17:10
7	Spectrum Quality	0.000
8	Spectrometer Frequency	400.33
9	Spectral Width	8012.8
10	Lowest Frequency	-1549.0
11	Nucleus	1H
12	Acquired Size	32768
13	Spectral Size	65536
14	Digital Resolution	0.12

Figure S16 ^{1}H NMR spectrum of compound $\mathbf{3^{Ts}}$ in CD_2Cl_2.. **^{1}H-NMR** (400 MHz, CD_2Cl_2): δ = 7.85 (d, $^3J_{HH}$ = 8.3 Hz, 2H, $CH_{tol,\mathit{ortho}}$), 7.25 (d, $^3J_{HH}$ = 7.8 Hz, 2H, $CH_{tol,\mathit{meta}}$), 3.65 (s, 32H, $CH_{2,crown}$+ residual THF), 2.38 (s, 3H, CH_3) ppm. The peaks at 3.67 ppm and 1.82 ppm correspond to residual THF.

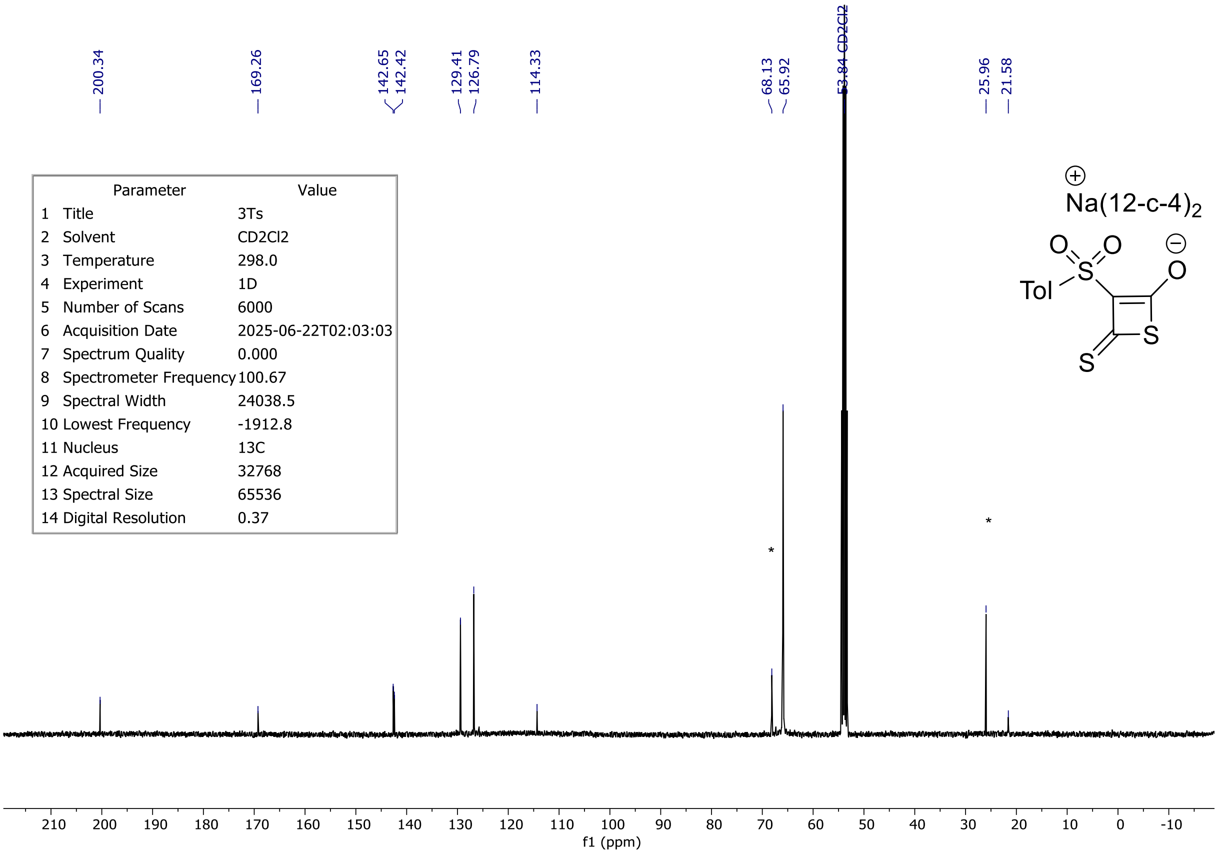

Figure S17 $^{13}C\{^1H\}$ NMR spectrum of compound $\mathbf{3^{Ts}}$ in CD_2Cl_2. $\mathbf{^{13}C\{^1H\}}$**-NMR** (101 MHz, CD_2Cl_2): δ = 200.3 (s, *C*=S), 169.3 (s, *C*=O), 142.7 (s, $C_{tol,para}$), 142.4 (s, $C_{tol,ipso}$), 129.4 (s, $CH_{tol,meta}$), 126.8 (s, $CH_{tol,ortho}$), 114.3 (s, *C*–SO_2tol), 65.9 (s, C_{crown}), 21.6 (s, CH_3) ppm.The peaks at 25.96 ppm and 68.13 ppm correspond to residual THF.

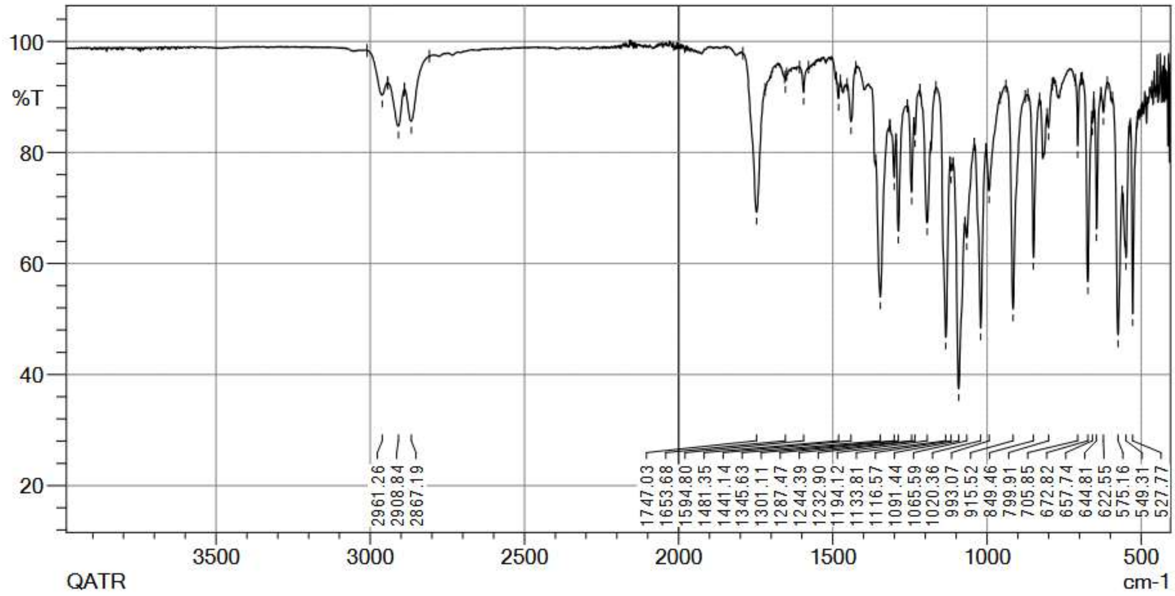

Figure S18 IR spectrum of compound $\mathbf{3^{Ts}}$ (solid state).

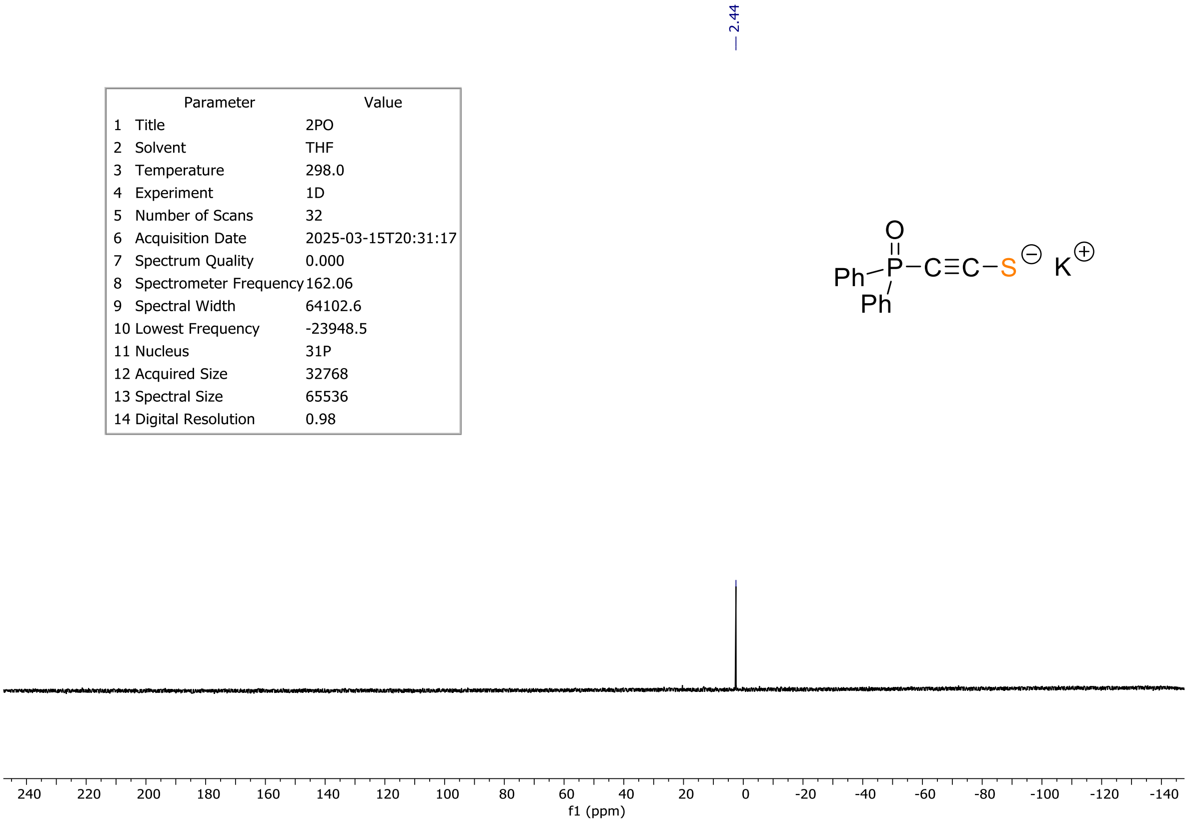

Figure S19 $^{31}P\{^1H\}$ NMR spectrum of compound **2**$^{\mathbf{PO}}$ in THF-d8. $\mathbf{^{31}P\{^1H\}}$**-NMR** (162 MHz, THF-d_8): δ = 2.44 (s, *P*Ph_2O) ppm.

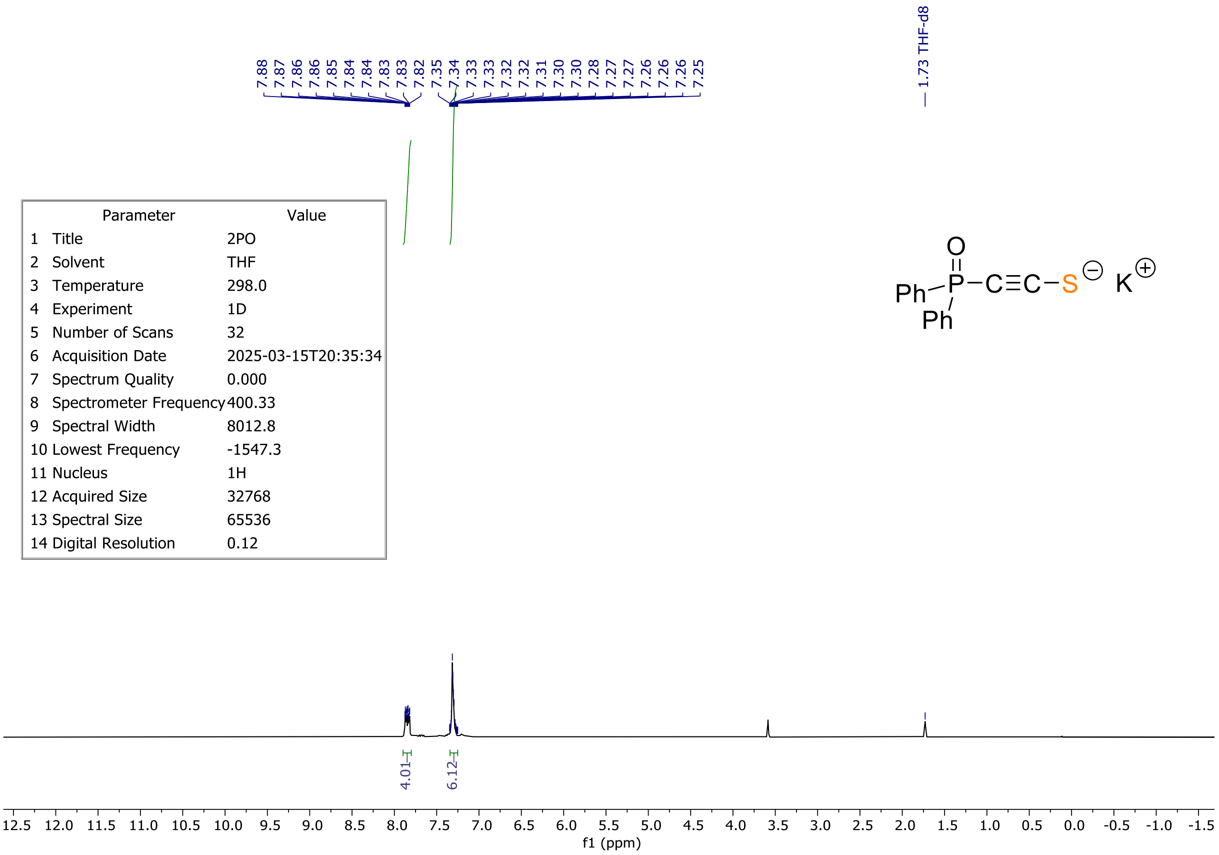

Figure S20 ^1H NMR spectrum of compound **2**$^{\mathbf{PO}}$ in THF-d8. $\mathbf{^1H}$**-NMR** (400 MHz, THF-d_8): δ = 7.88 – 7.82 (m, 4H, C*H*$_{Ph,\mathit{ortho}}$), 7.35 – 7.25 (m, 6H, C*H*$_{Ph,\mathit{meta,para}}$) ppm.

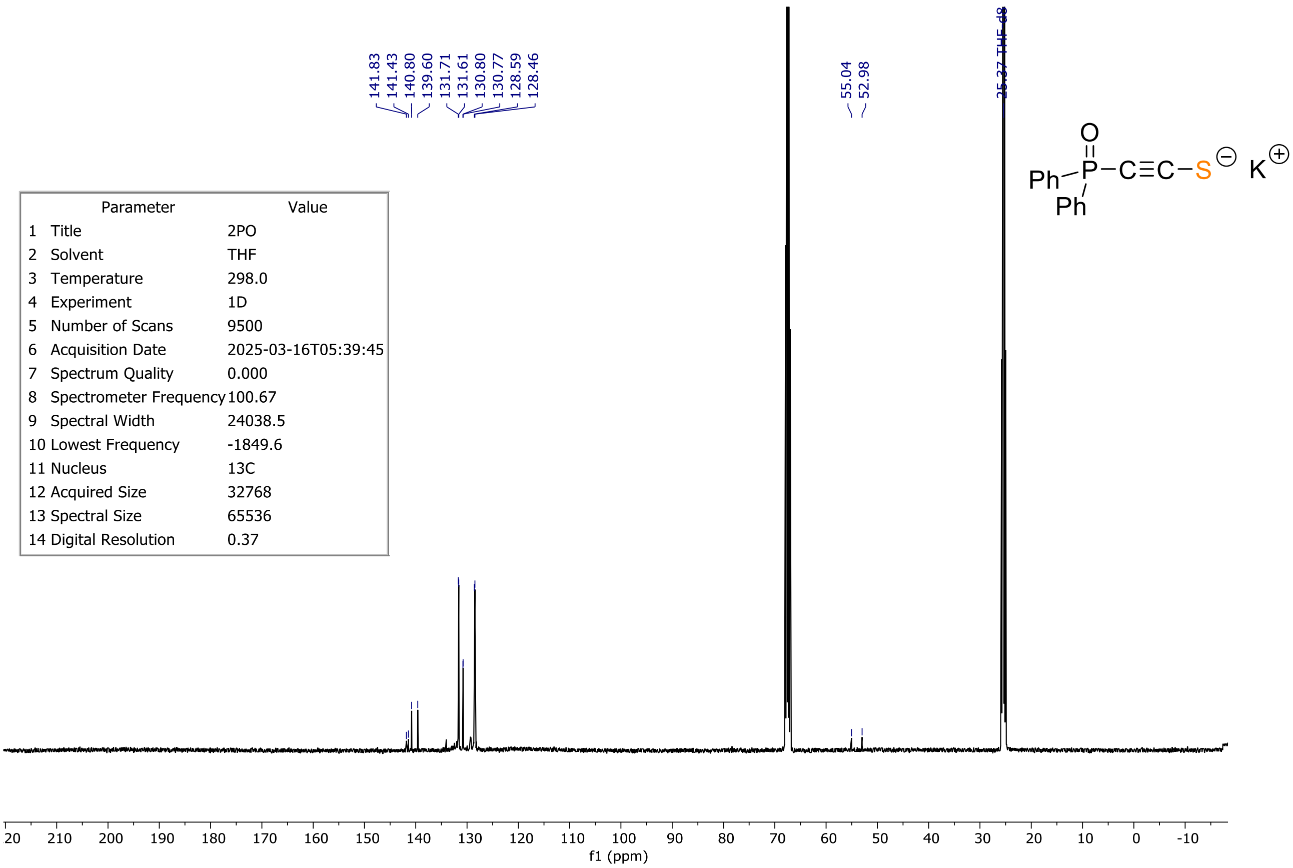

Figure S21 $^{13}C\{^{1}H\}$ NMR spectrum of compound $\mathbf{2^{PO}}$ in THF-d8. $\mathbf{^{13}C\{^{1}H\}}$**-NMR** (101 MHz, THF-d_8): δ = 141.63(d, $^{2}J_{CP}$ = 39.9 Hz, PCCS), 140.20 (d, $^{1}J_{CP}$ = 119.9 Hz, $C_{Ph,ipso}$), 131.66 (d, $^{2}J_{CP}$ = 10.9 Hz, $CH_{Ph,ortho}$), 130.78 (d, $^{4}J_{CP}$ = 2.7 Hz, $CH_{Ph,para}$),128.53 (d, $^{3}J_{CP}$ = 12.8 Hz, $CH_{Ph,meta}$), 54.01 (d, $^{1}J_{CP}$ = 207.6 Hz, PCCS) ppm.

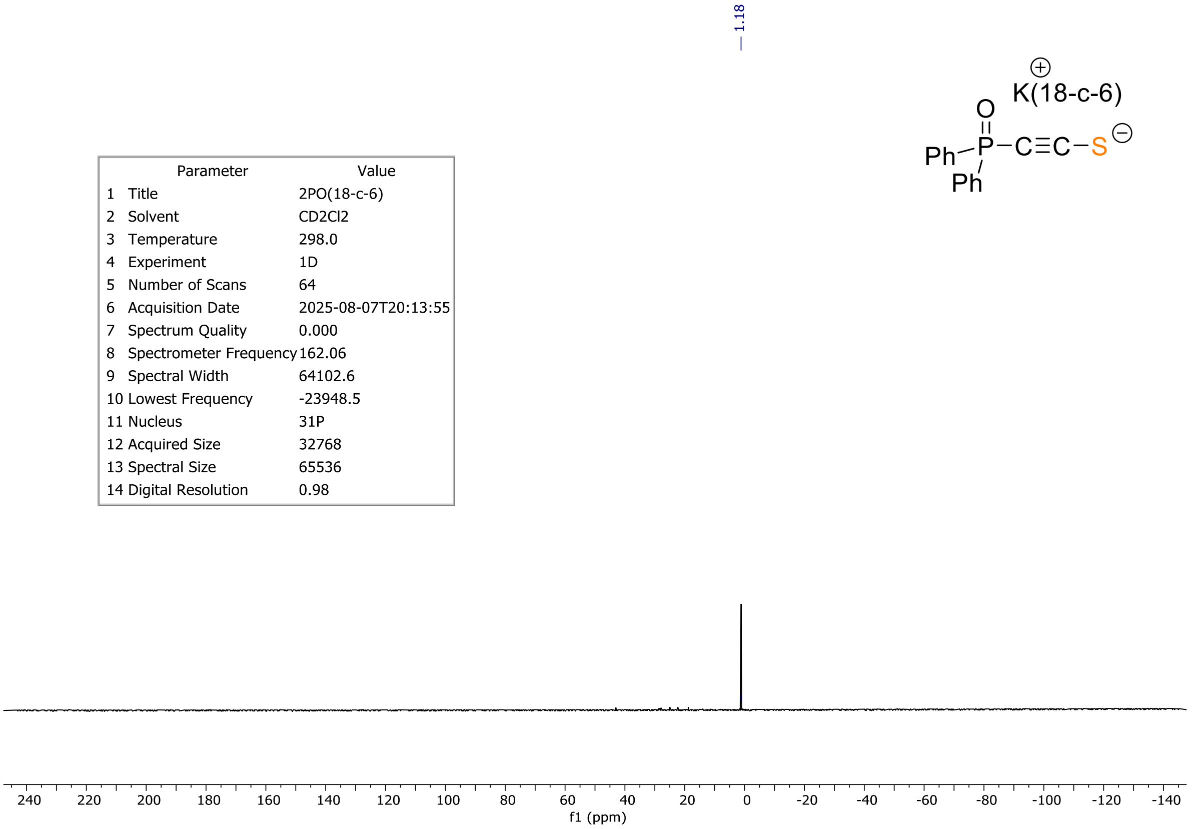

Figure S22 $^{31}P\{^1H\}$ NMR spectrum of compound $\mathbf{2^{PO}(18\text{-}c\text{-}6)}$ in CD_2Cl_2. $\mathbf{^{31}P\{^1H\}}$**-NMR** (162 MHz, CD_2Cl_2): δ = 1.18 (s, *P*Ph_2O) ppm.

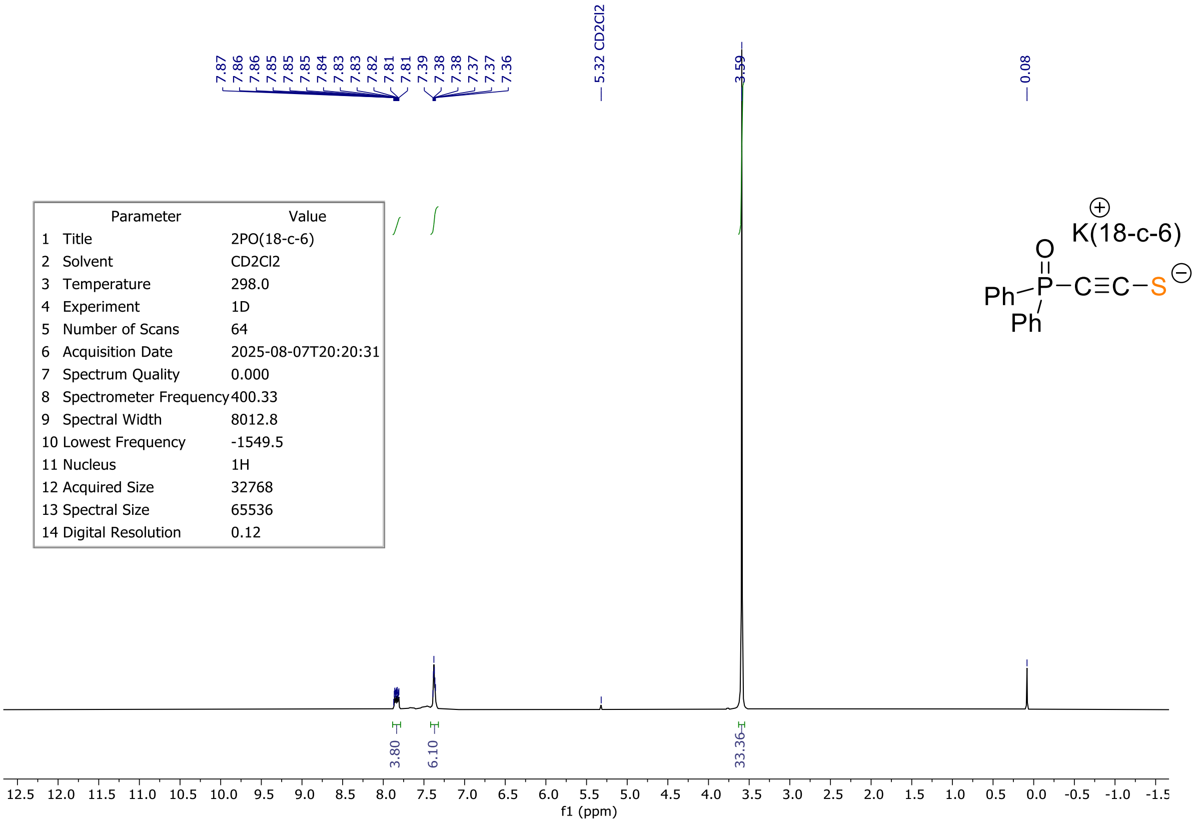

Figure S23 ^1H NMR spectrum of compound $\mathbf{2^{PO}(18\text{-}c\text{-}6)}$ in CD_2Cl_2. $\mathbf{^1H}$**-NMR** (400 MHz, THF-d_8): δ = 8.07 – 8.01 (m, 4H, $CH_{Ph,\mathit{ortho}}$), 7.26 – 7.23 (m, 6H, $CH_{Ph,\mathit{meta,para}}$), 3.59 (s, 32H, $CH_{2,crown}$+ residual THF) ppm.

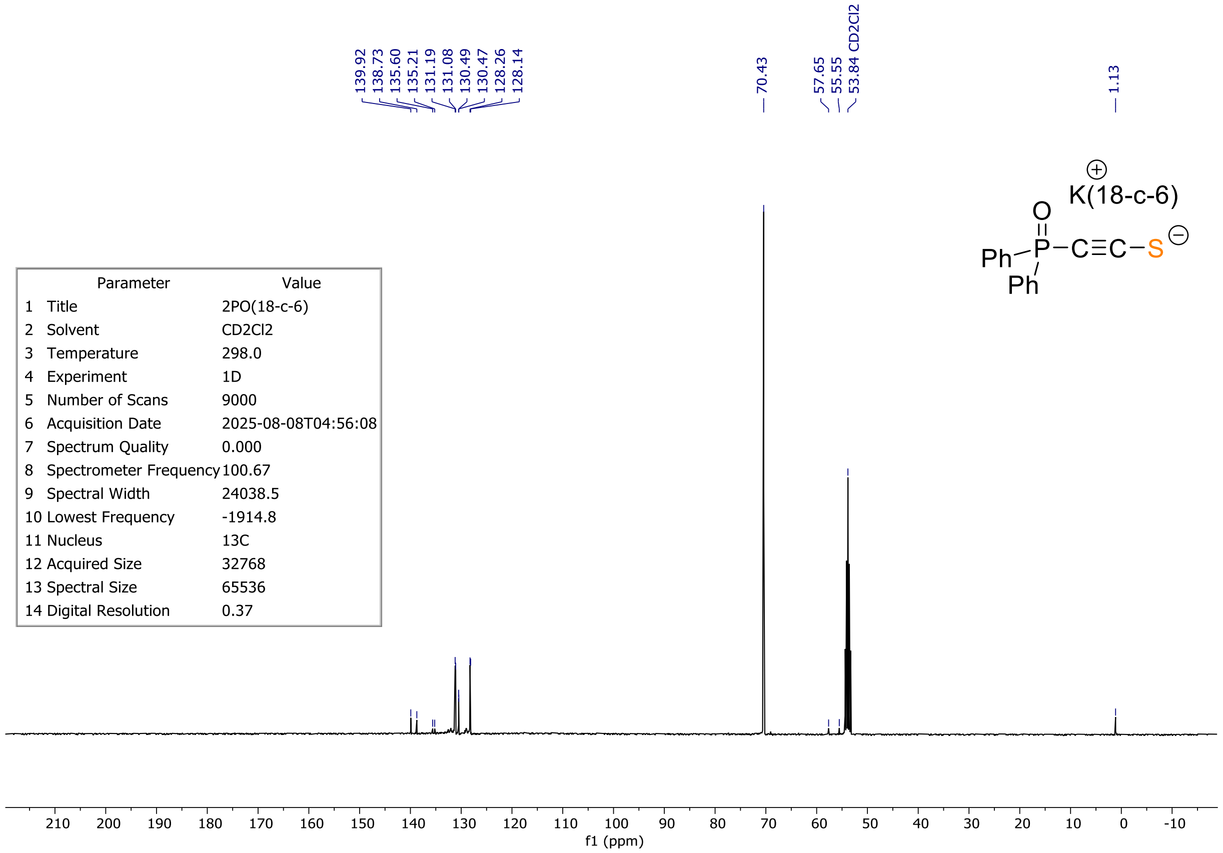

Figure S24 $^{13}C\{^{1}H\}$ NMR spectrum of compound **2^{PO}(18-c-6)** in CD_2Cl_2. **$^{13}C\{^{1}H\}$-NMR** (101 MHz, CD_2Cl_2): δ = 139.33 (d, $^{1}J_{CP}$ = 119.3 Hz, $C_{Ph,ipso}$), 135.41(d, $^{2}J_{CP}$ = 39.6 Hz, PC*C*S), 131.14 (d, $^{2}J_{CP}$ = 10.8 Hz, $CH_{Ph,ortho}$), 130.48 (d, $^{4}J_{CP}$ = 2.8 Hz, $CH_{Ph,para}$),128.20 (d, $^{3}J_{CP}$ = 12.6 Hz, $CH_{Ph,meta}$), 70.43 (s, C_{crown}), 56.60 (d, $^{1}J_{CP}$ = 211.7 Hz, P*C*CS) ppm.

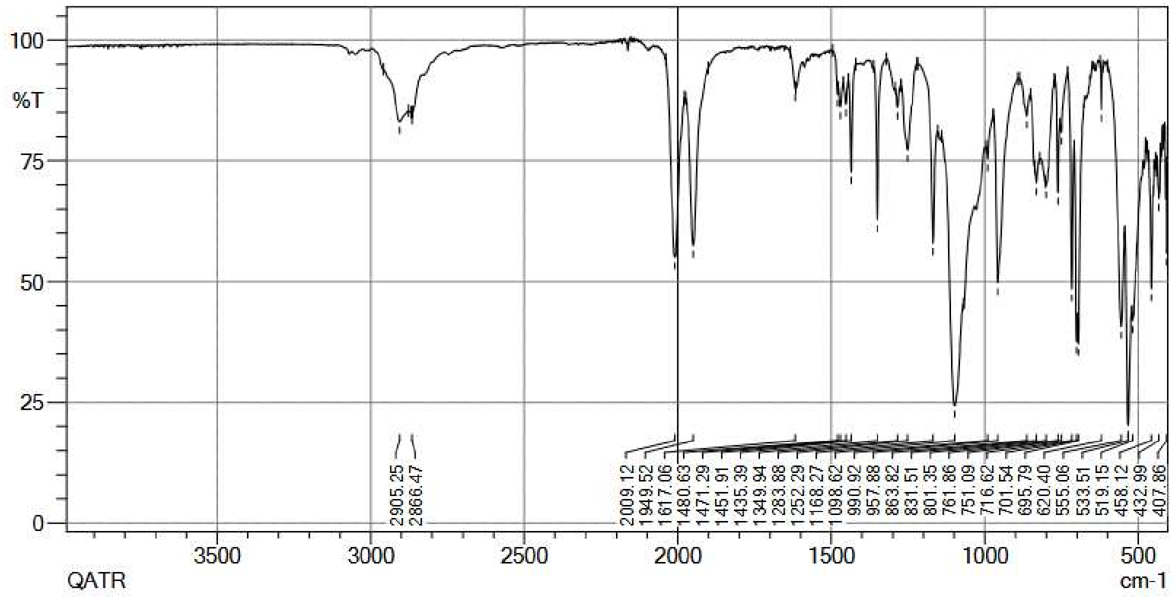

Figure S25 IR spectrum of compound **2^{PO}(18-c-6)** (solid state).

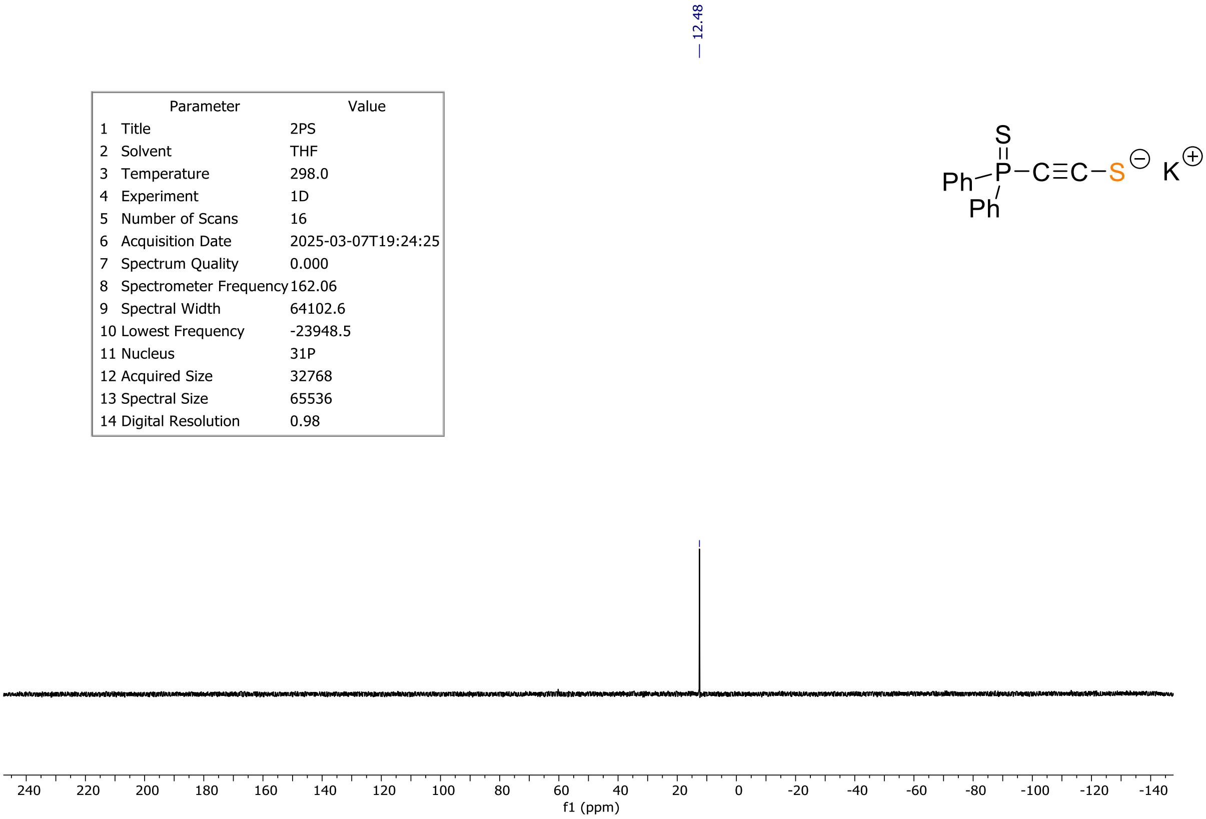

Figure S26 ^{31}P{^{1}H} NMR spectrum of compound **2^{PS}** in THF-d$_8$. **^{31}P{^{1}H}-NMR** (162 MHz, THF-*d*$_8$): δ = 12.48 (s, *P*Ph$_2$S) ppm.

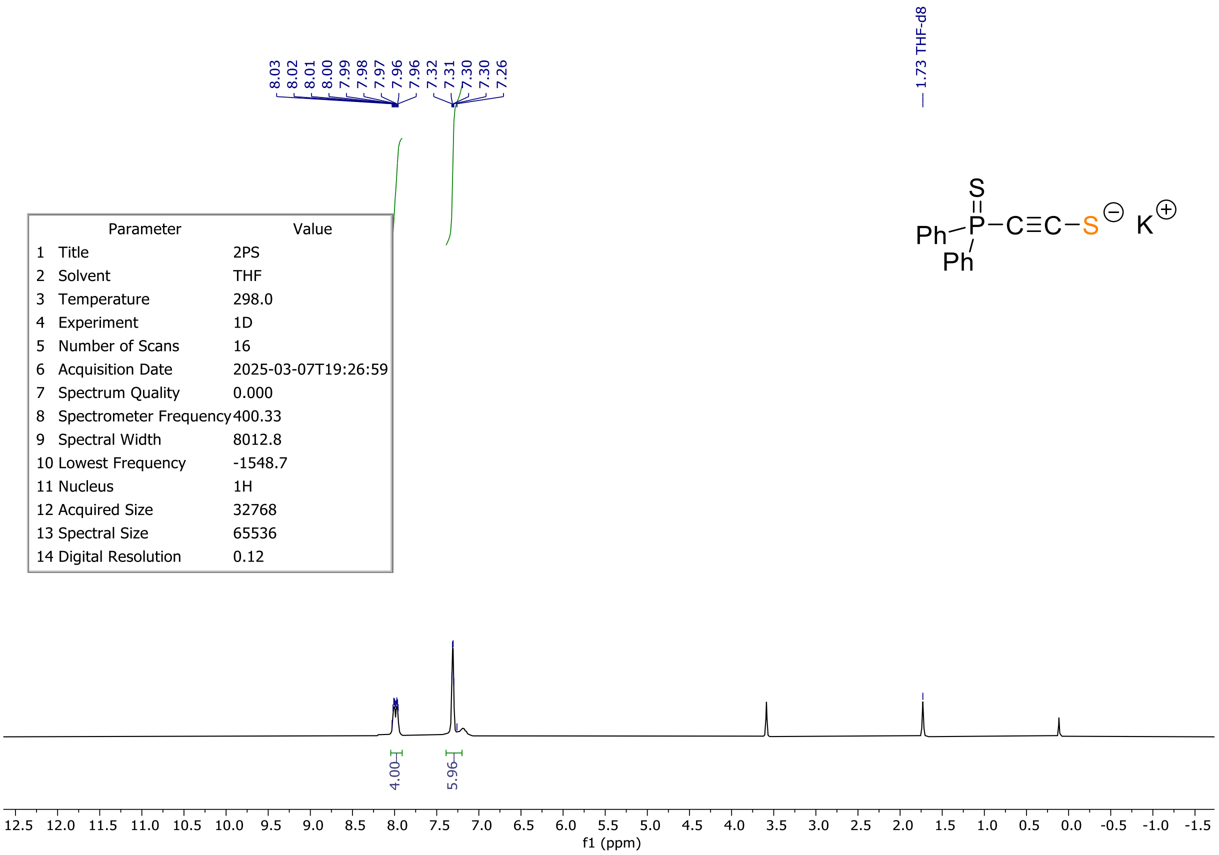

Figure S27 ^{1}H NMR spectrum of compound **2^{PS}** in THF-d$_8$. **^{1}H-NMR** (400 MHz, THF-d$_8$): δ = 8.03 – 7.96 (m, 4H, C*H*$_{Ph,\mathit{ortho}}$), 7.32 – 7.26 (m, 6H, C*H*$_{Ph,\mathit{meta,para}}$) ppm

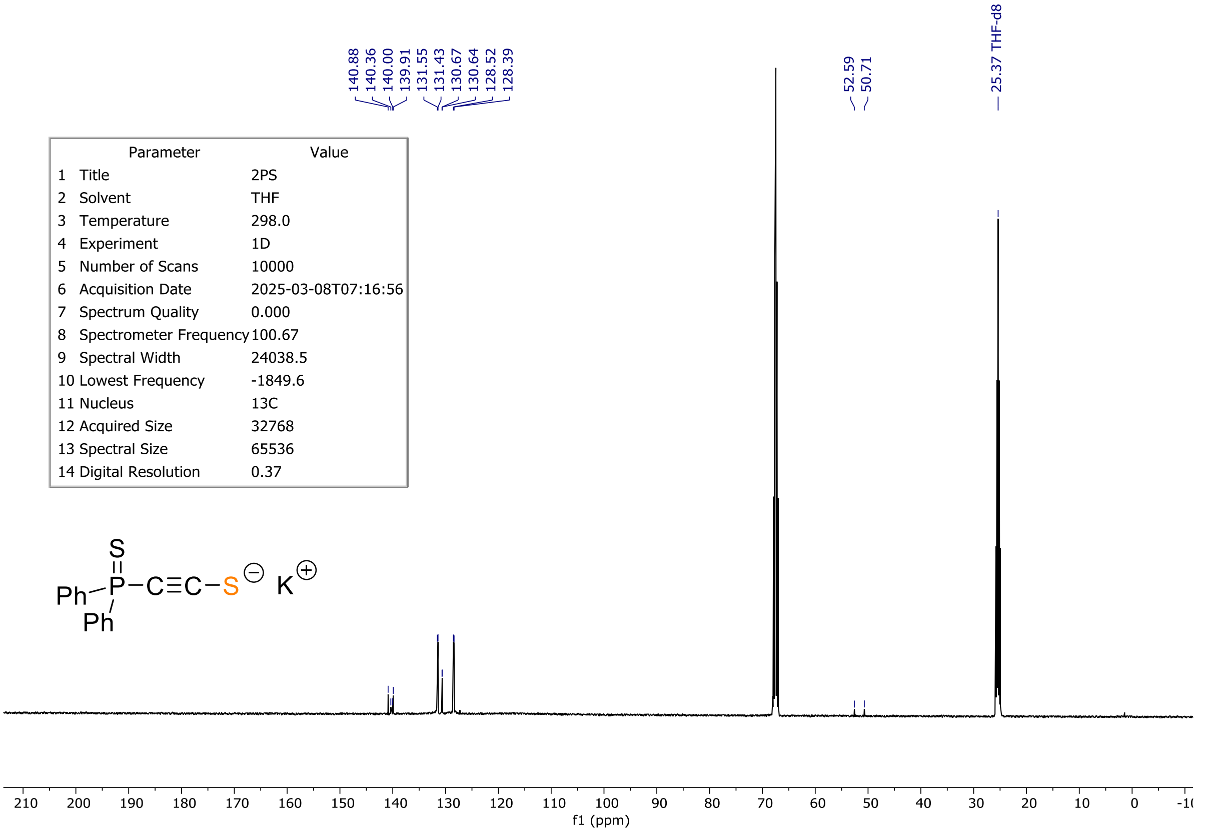

Figure S28 $^{13}C\{^1H\}$ NMR spectrum of compound $\mathbf{2^{PS}}$ in THF-d_8. $\mathbf{^{13}C\{^1H\}}$**-NMR** (101 MHz, THF-d_8): δ = 140.39 (d, $^1J_{CP}$ = 97.5 Hz, $C_{Ph,ipso}$), 140.18 (d, $^2J_{CP}$ = 36.0 Hz, PC*C*S), 131.49 (d, $^2J_{CP}$ = 12.1 Hz, $CH_{Ph,ortho}$), 130.65 (d, $^4J_{CP}$ = 2.7 Hz, $CH_{Ph,para}$),128.45 (d, $^3J_{CP}$ = 13.1 Hz, $CH_{Ph,meta}$), 51.65 (d, $^1J_{CP}$ = 189.8 Hz, P*C*CS) ppm.

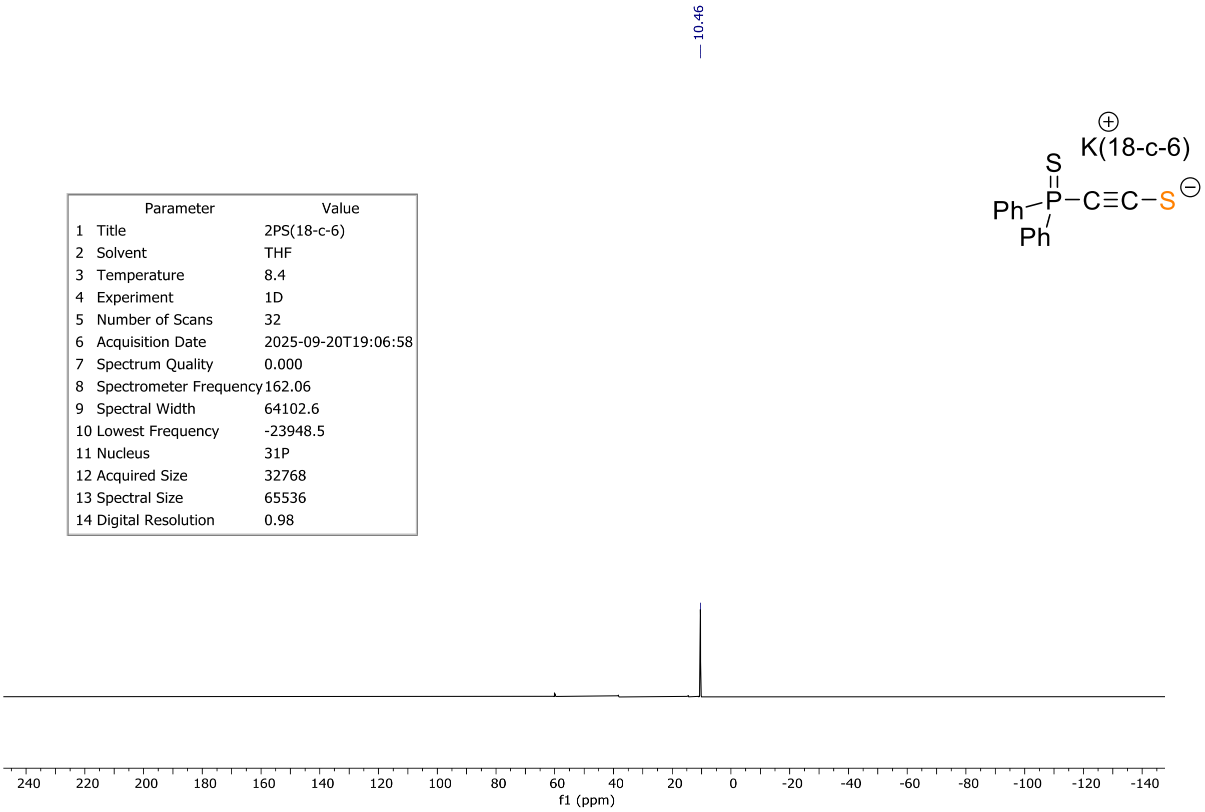

Figure S29 $^{31}P\{^1H\}$ NMR spectrum of compound $\mathbf{2^{PS}(18\text{-}c\text{-}6)}$ in THF-d_8. $\mathbf{^{31}P\{^1H\}}$**-NMR** (162 MHz, THF-d_8): δ = 10.46 (s, *P*Ph_2S) ppm.

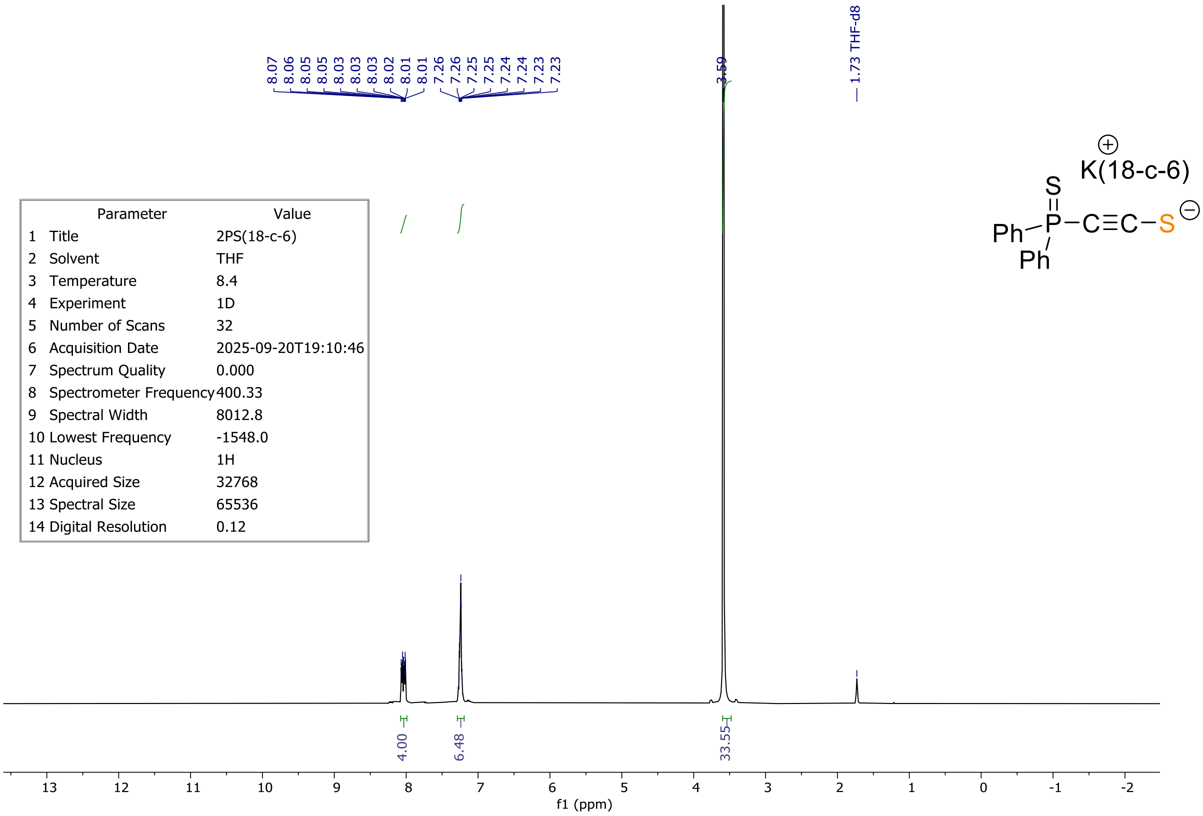

Figure S30 ^{1}H NMR spectrum of compound **2^{PS}(18-c-6)** in THF-d$_8$. **^{1}H-NMR** (400 MHz, THF-*d*$_8$): δ = 8.07 – 8.01 (m, 4H, C*H*$_{Ph,ortho}$), 7.26 – 7.23 (m, 6H, C*H*$_{Ph,meta,para}$), 3.59 (s, 32H, C*H*$_{2,crown}$+ residual THF) ppm.

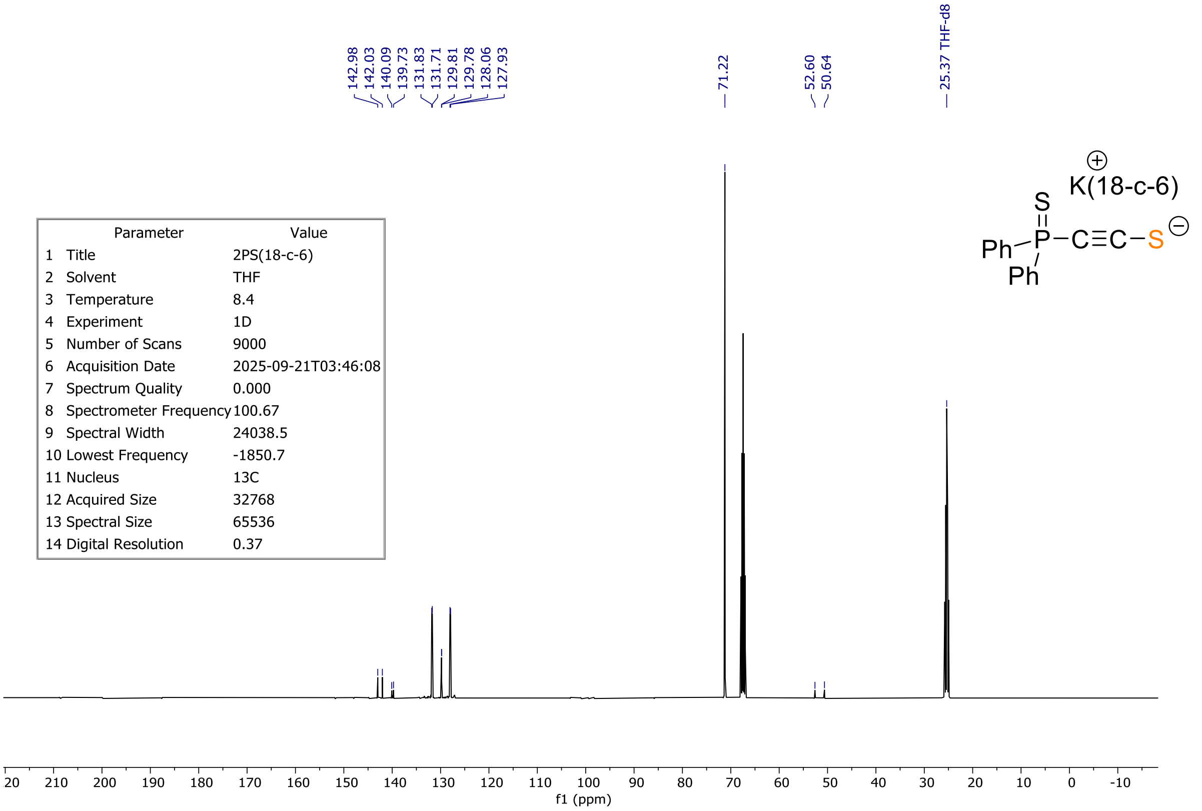

Figure S31 ^{13}C{^{1}H} NMR spectrum of compound **2^{PS}(18-c-6)** in THF-d$_8$. **^{13}C{^{1}H}-NMR** (101 MHz, THF-d$_8$): δ = 142.51 (d, $^{1}J_{CP}$ = 95.6 Hz, *C*$_{Ph,ipso}$), 139.91 (d, $^{2}J_{CP}$ = 36.7 Hz, PC*C*S), 131.77 (d, $^{2}J_{CP}$ = 11.8 Hz, *C*H$_{Ph,ortho}$), 129.79 (d, $^{4}J_{CP}$ = 2.9 Hz, *C*H$_{Ph,para}$),127.99 (d, $^{3}J_{CP}$ = 12.9 Hz, *C*H$_{Ph,meta}$), 71.22 (s, *C*$_{crown}$), 51.62 (d, $^{1}J_{CP}$ = 198.0 Hz, P*C*CS) ppm

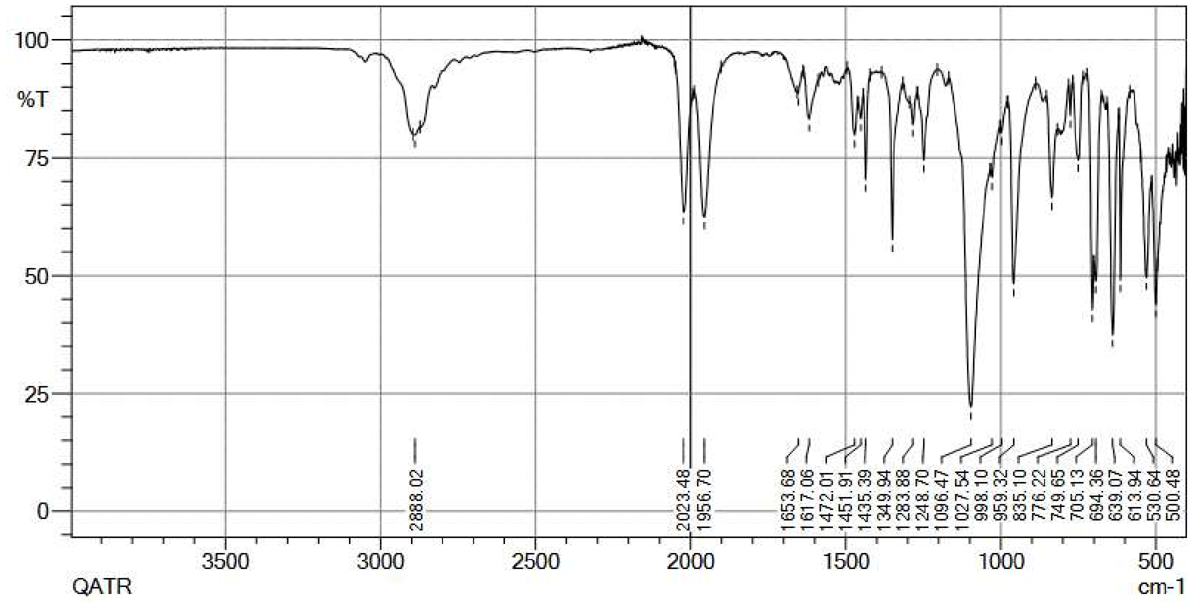

Figure S32 IR spectrum of compound **2^{PS}(18-c-6)** (solid state).

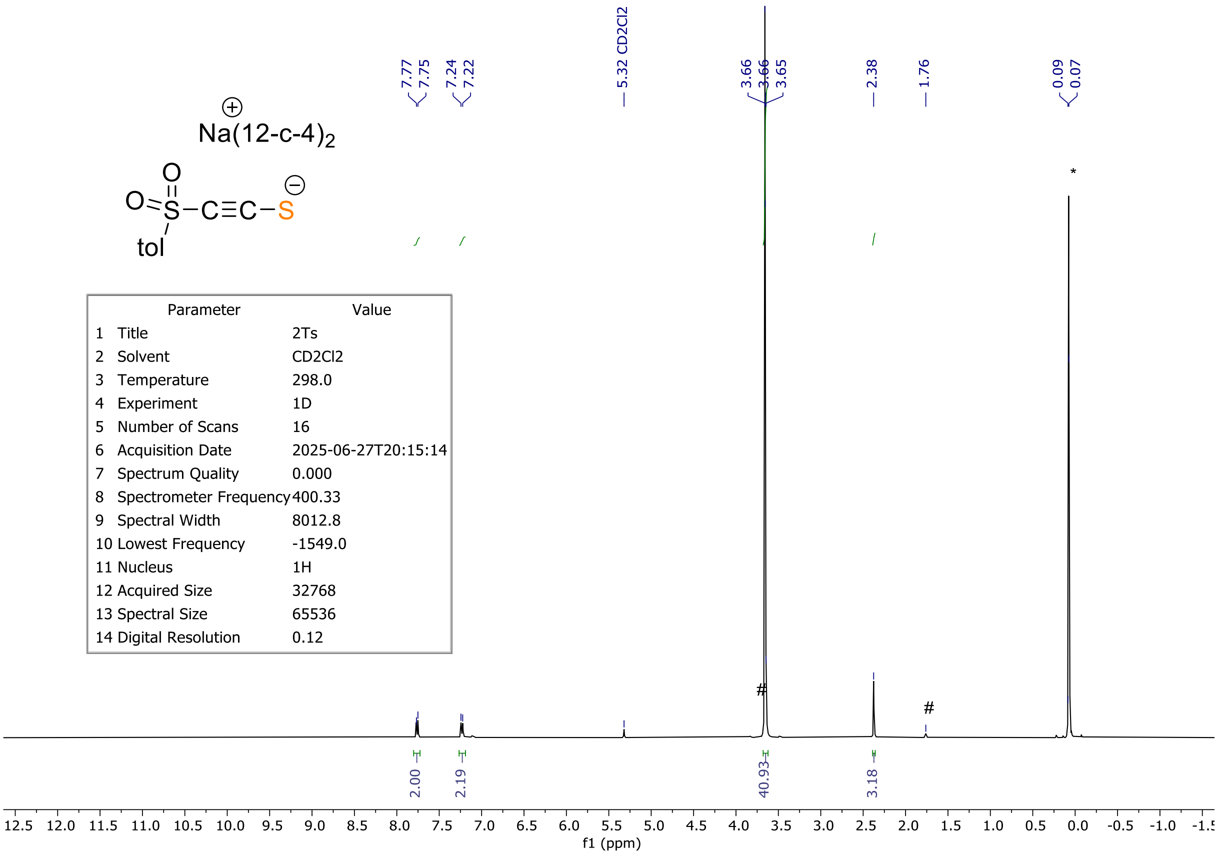

	Parameter	Value
1	Title	2Ts
2	Solvent	CD2Cl2
3	Temperature	298.0
4	Experiment	1D
5	Number of Scans	16
6	Acquisition Date	2025-06-27T20:15:14
7	Spectrum Quality	0.000
8	Spectrometer Frequency	400.33
9	Spectral Width	8012.8
10	Lowest Frequency	-1549.0
11	Nucleus	1H
12	Acquired Size	32768
13	Spectral Size	65536
14	Digital Resolution	0.12

Figure S33 ^{1}H NMR spectrum of compound **2^{Ts}** in CD_2Cl_2. **^{1}H-NMR** (400 MHz, CD_2Cl_2): δ = 7.76 (d, $^3J_{HH}$ = 8.4 Hz, 2H, C$H_{tol,ortho}$), 7.23 (d, $^3J_{HH}$ = 8.3 Hz, 2H, C$H_{tol,meta}$), 3.66 (s, 32H, C$H_{2,crown}$+ residual THF), 2.38 (s, 3H, CH_3) ppm.The peak at 0.08 ppm corresponds to silicone grease.(# = THF-d_8).

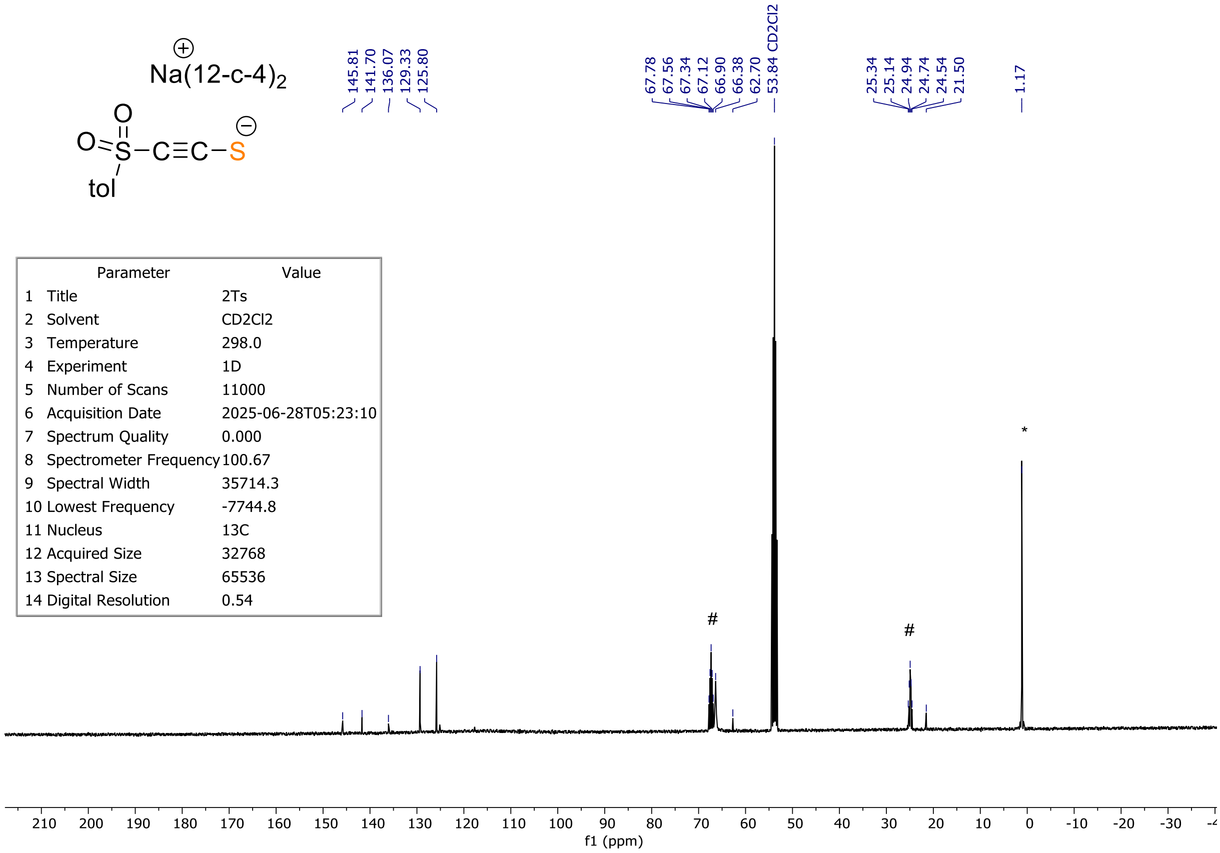

Figure S34 $^{13}C\{^{1}H\}$ NMR spectrum of compound **2^{Ts}** in CD_2Cl_2. **$^{13}C\{^{1}H\}$-NMR** (101 MHz, CD_2Cl_2): δ = 145.8 (s, C*C*S), 141.7 (s, $C_{tol,para}$), 136.1 (s, $C_{tol,ipso}$), 129.3 (s, $CH_{tol,meta}$), 125.8 (s, $CH_{tol,ortho}$), 66.4 (s, C_{crown}), 62.7 (s, *C*–SO_2tol), 21.5 (s, CH_3) ppm. The peak at 1.17 ppm corresponds to silicone grease. (# = THF-d_8).

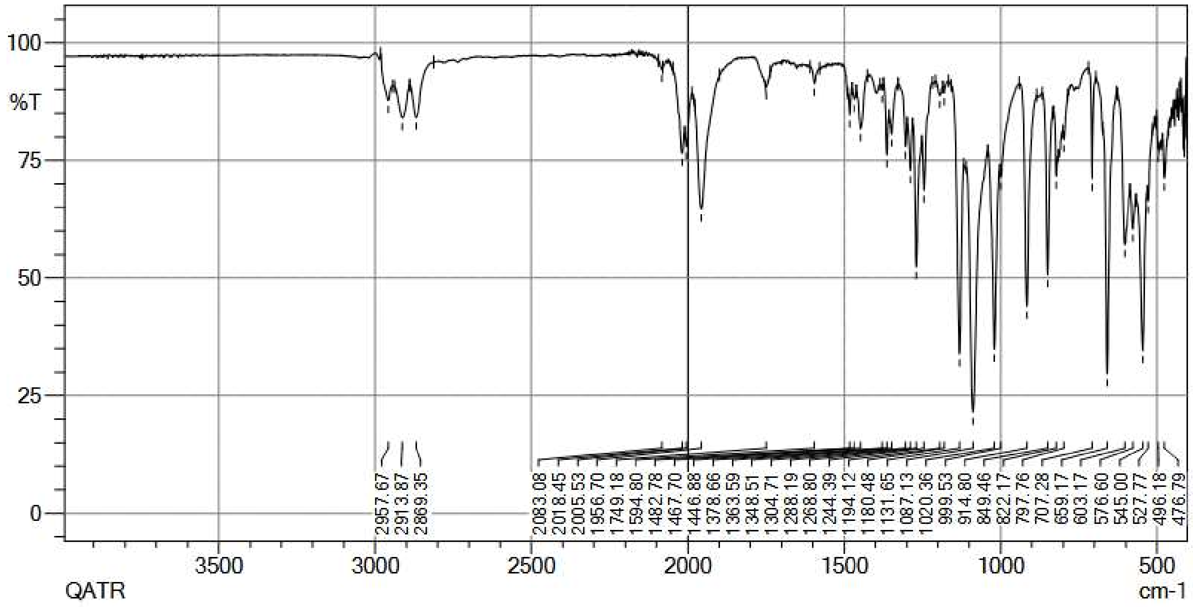

Figure S35 IR spectrum of compound **2^{Ts}** (solid state).

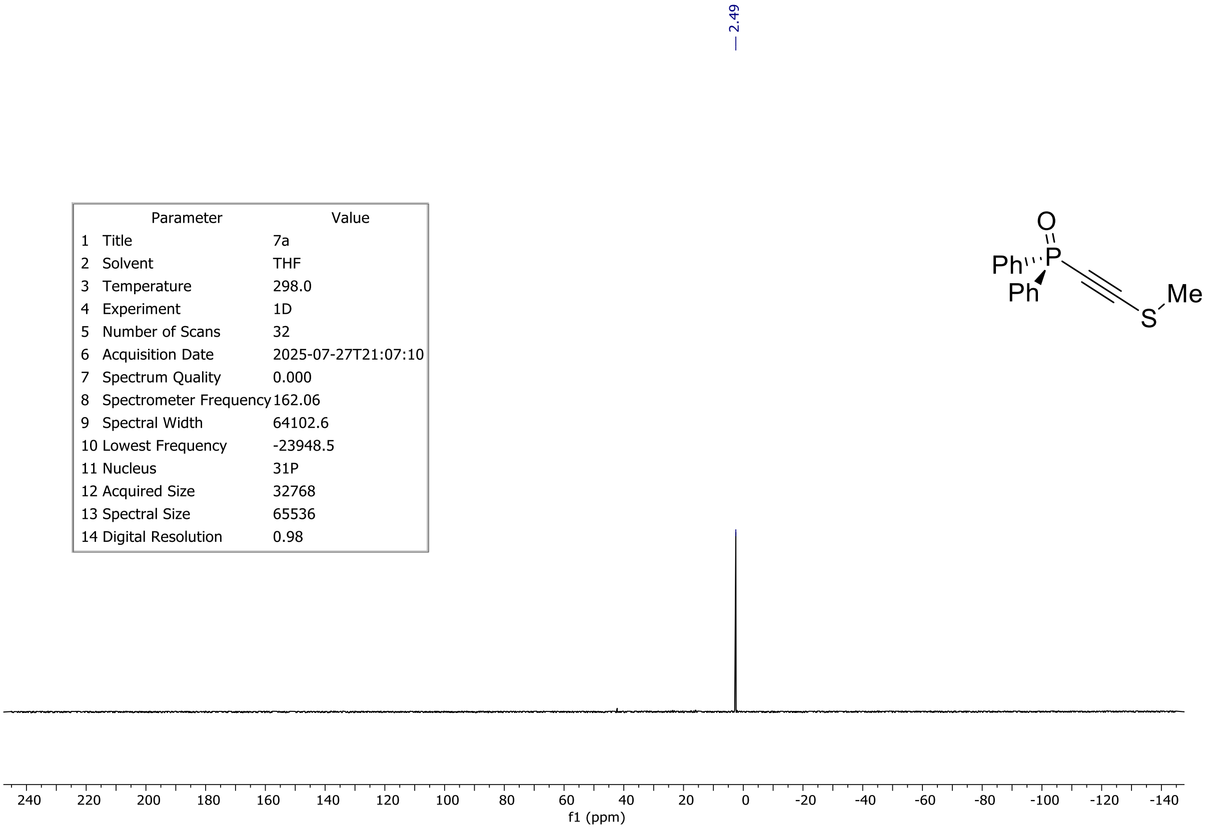

	Parameter	Value
1	Title	7a
2	Solvent	THF
3	Temperature	298.0
4	Experiment	1D
5	Number of Scans	32
6	Acquisition Date	2025-07-27T21:07:10
7	Spectrum Quality	0.000
8	Spectrometer Frequency	162.06
9	Spectral Width	64102.6
10	Lowest Frequency	-23948.5
11	Nucleus	31P
12	Acquired Size	32768
13	Spectral Size	65536
14	Digital Resolution	0.98

Figure S36 $^{31}P\{^1H\}$ NMR spectrum of compound **7a** in THF-d_8. **$^{31}P\{^1H\}$-NMR** (162 MHz, THF-d_8): δ = 2.49 (s, *P*Ph_2O) ppm.

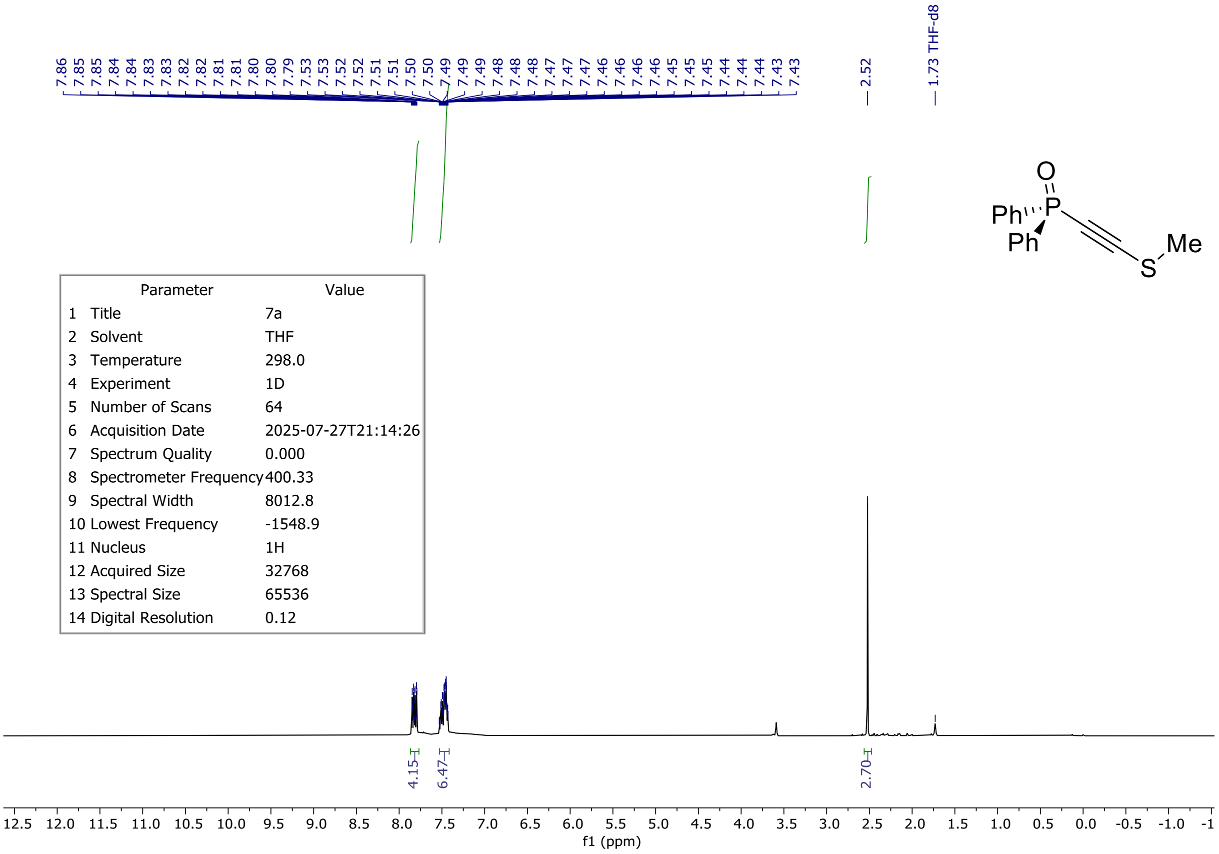

	Parameter	Value
1	Title	7a
2	Solvent	THF
3	Temperature	298.0
4	Experiment	1D
5	Number of Scans	64
6	Acquisition Date	2025-07-27T21:14:26
7	Spectrum Quality	0.000
8	Spectrometer Frequency	400.33
9	Spectral Width	8012.8
10	Lowest Frequency	-1548.9
11	Nucleus	1H
12	Acquired Size	32768
13	Spectral Size	65536
14	Digital Resolution	0.12

Figure S37 ^1H NMR spectrum of compound **7a** in THF-d_8. **^1H-NMR** (400 MHz, THF-d_8): δ = 7.86 – 7.79 (m, 4H, C*H*$_{Ph,\mathit{ortho}}$), 7.53 – 7.43 (m, 6H, C*H*$_{Ph,\mathit{meta,para}}$), 2.52 (s, 3H, C*H*$_3$) ppm.

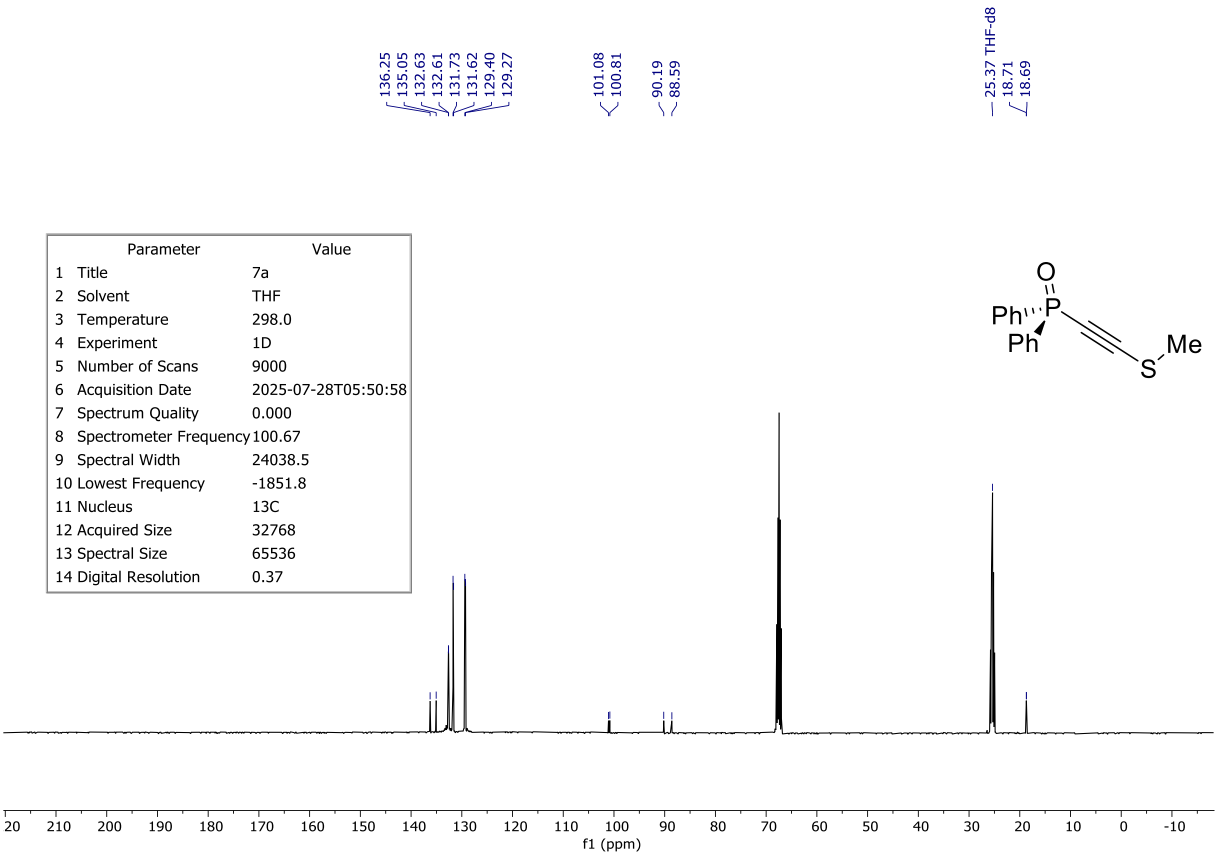

Figure S38 $^{13}C\{^{1}H\}$ NMR spectrum of compound **7a** in THF-d_8. **^{13}C{^{1}H}-NMR** (101 MHz, THF-d_8): δ = 135.65 (d, $^{1}J_{CP}$ = 120.7 Hz, $C_{Ph,ipso}$), 132.62 (d, $^{4}J_{CP}$ = 3.0 Hz, $CH_{Ph,para}$), 131.67 (d, $^{2}J_{CP}$ = 10.7 Hz, $CH_{Ph,ortho}$), 129.34 (d, $^{3}J_{CP}$ = 13.1 Hz, $CH_{Ph,meta}$), 100.94 (d, $^{2}J_{CP}$ = 26.2 Hz, PC*C*S), 89.39 (d, $^{1}J_{CP}$ = 161.2 Hz, P*C*CS), 18.70 (d, $^{4}J_{CP}$ = 1.9 Hz, CH_3) ppm.

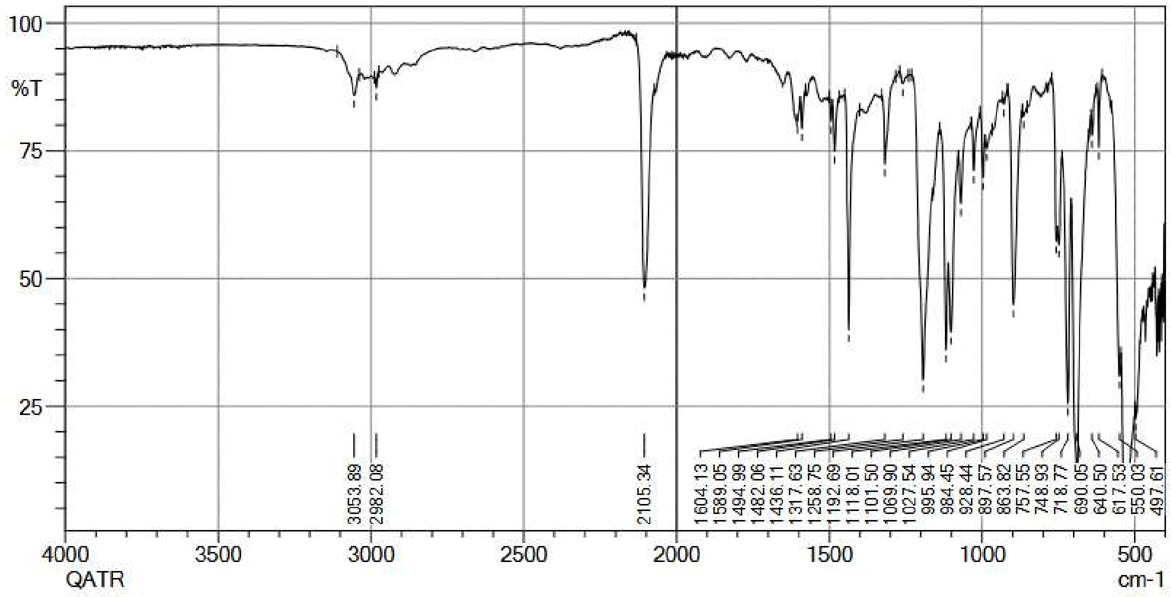

Figure S39 IR spectrum of compound **7a** (solid state).

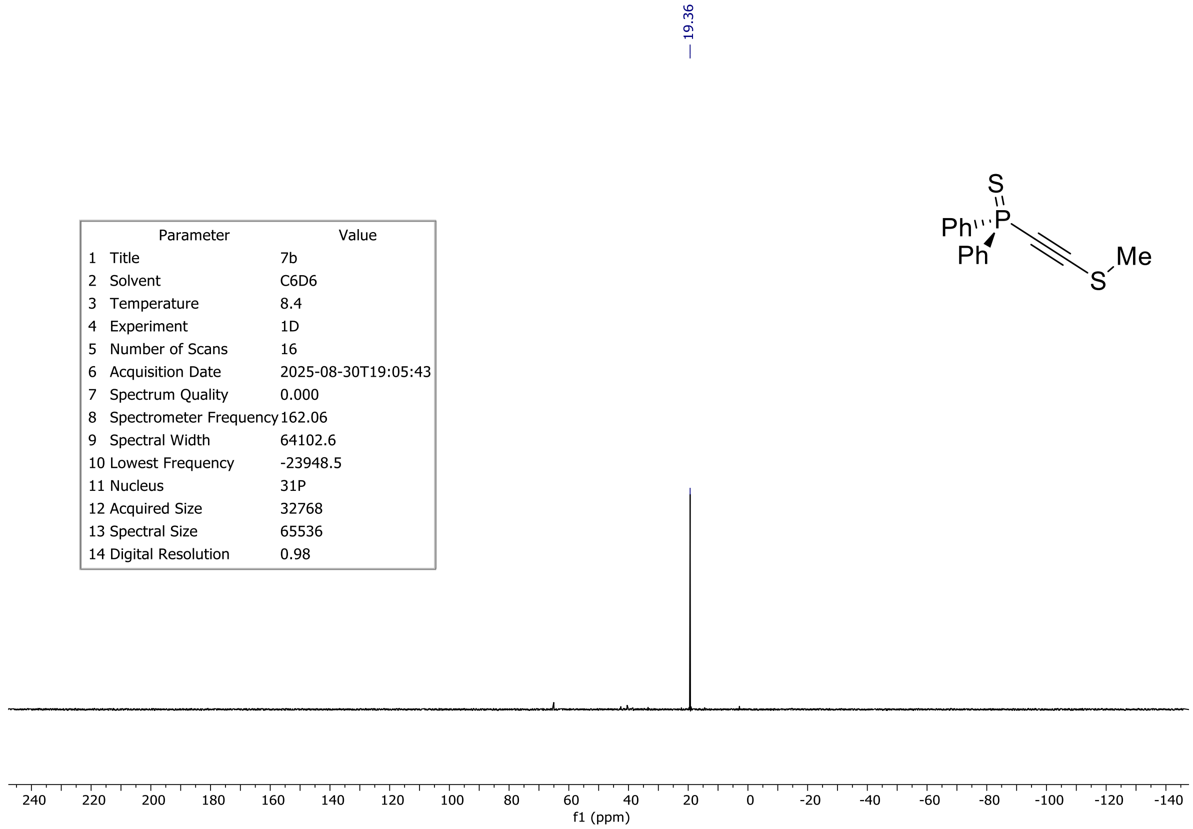

Figure S40 $^{31}P\{^{1}H\}$ NMR spectrum of compound **7b** in C_6D_6. **^{31}P{^{1}H}-NMR** (162 MHz, C_6D_6): δ = 19.36 (s, *P*Ph_2S) ppm.

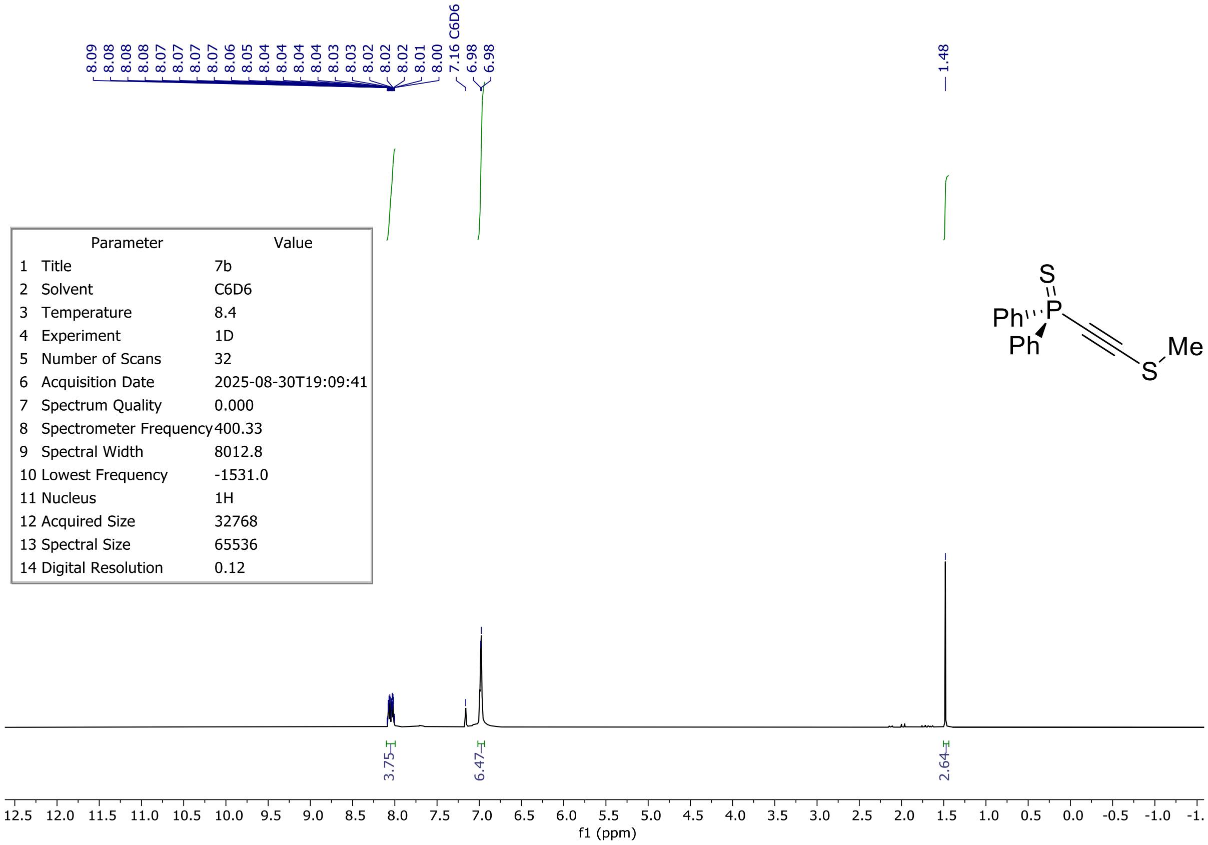

Figure S41 ^{1}H NMR spectrum of compound **7b** in C_6D_6. **^{1}H-NMR** (400 MHz, C_6D_6): δ = 8.09 – 8.00 (m, 4H, C*H*$_{Ph,\textit{ortho}}$), 6.99 – 6.98 (m, 6H, C*H*$_{Ph,\textit{meta,para}}$), 1.48 (s, 3H, CH_3) ppm.

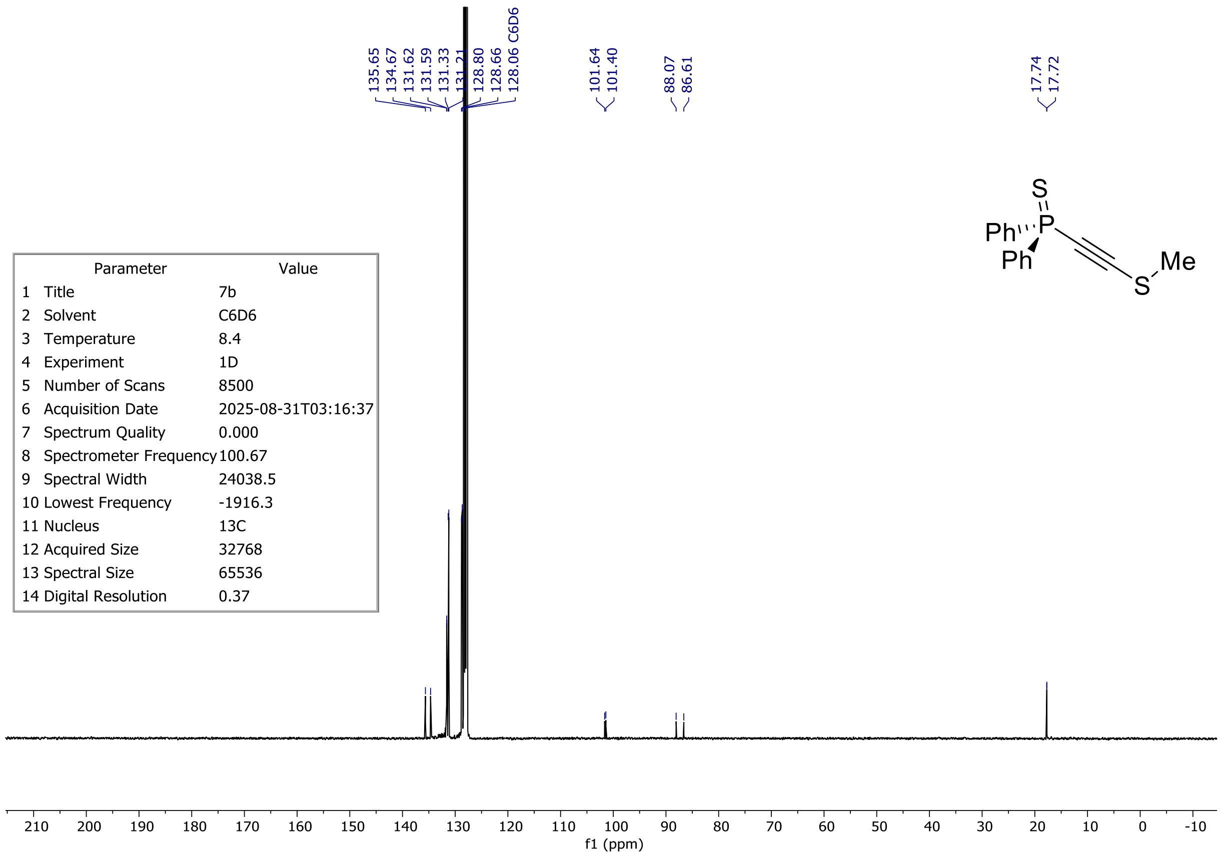

Figure S42 $^{13}C\{^{1}H\}$ NMR spectrum of compound **7b** in C_6D_6. $^{\mathbf{13}}\mathbf{C\{^{1}H\}}$**-NMR** (101 MHz, C_6D_6): δ = 135.16 (d, $^{1}J_{CP}$ = 98.3 Hz, $C_{Ph,ipso}$), 131.60 (d, $^{4}J_{CP}$ = 3.2 Hz, $CH_{Ph,para}$), 131.27 (d, $^{2}J_{CP}$ = 12.1 Hz, $CH_{Ph,ortho}$), 128.73 (d, $^{3}J_{CP}$ = 13.6 Hz, $CH_{Ph,meta}$), 101.52 (d, $^{2}J_{CP}$ = 23.6 Hz, PC*C*S), 87.34 (d, $^{1}J_{CP}$ = 146.9 Hz, P*C*CS), 17.73 (d, $^{4}J_{CP}$ = 2.1 Hz, CH_3) ppm.

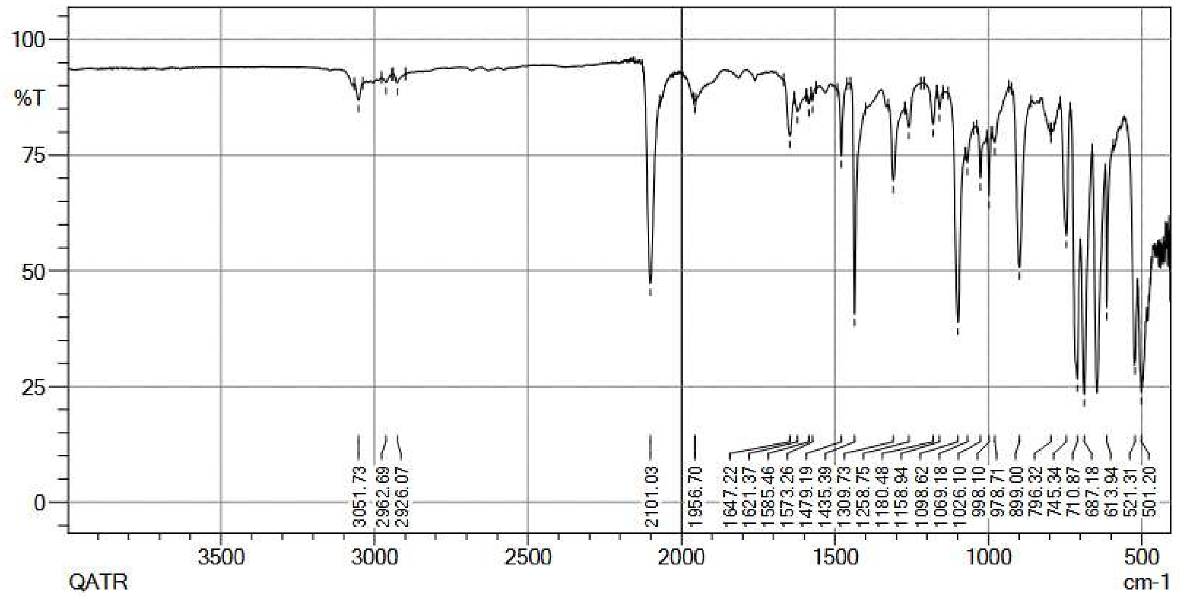

Figure S43 IR spectrum of compound **7b** (solid state).

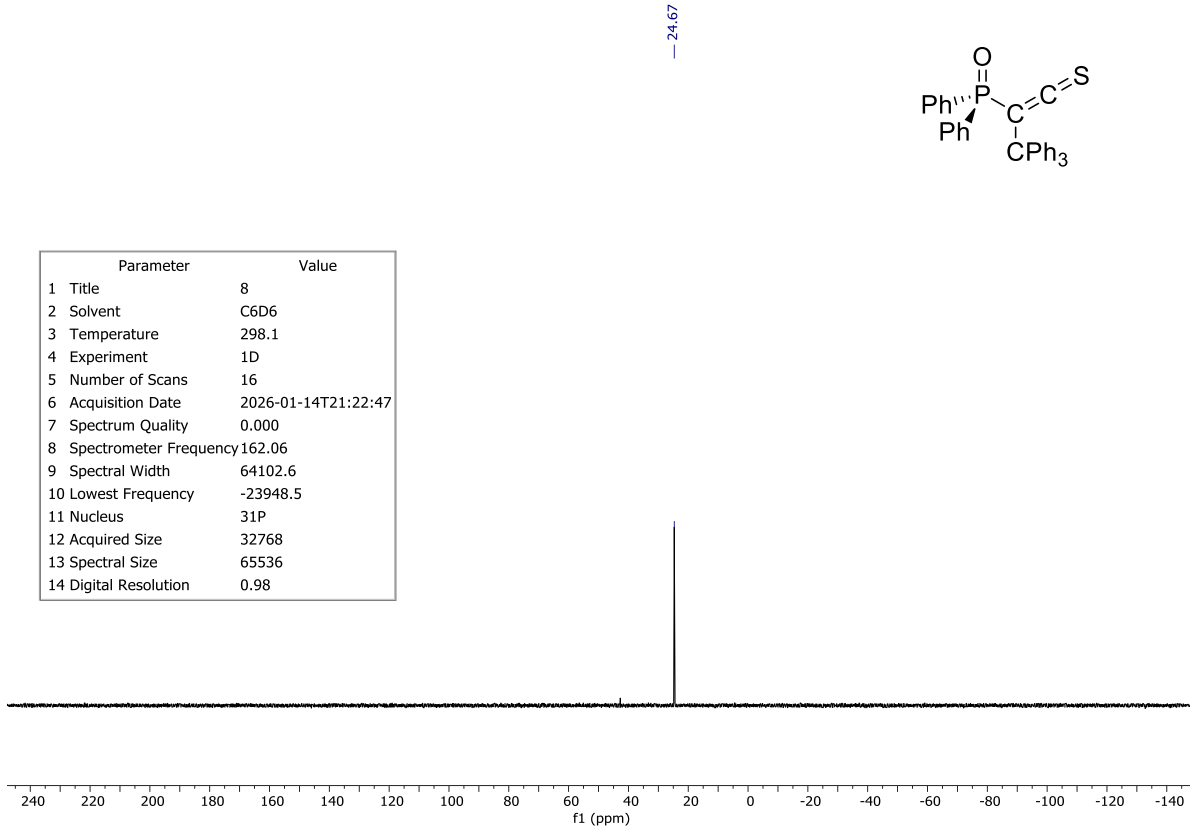

Figure S 44 $^{31}P\{^1H\}$ NMR spectrum of compound **8** + CPh_3Cl in C_6D_6. $\mathbf{^{31}P\{^1H\}}$**-NMR** (162 MHz, C_6D_6): δ = 24.67 (s, *P*Ph_2O) ppm.

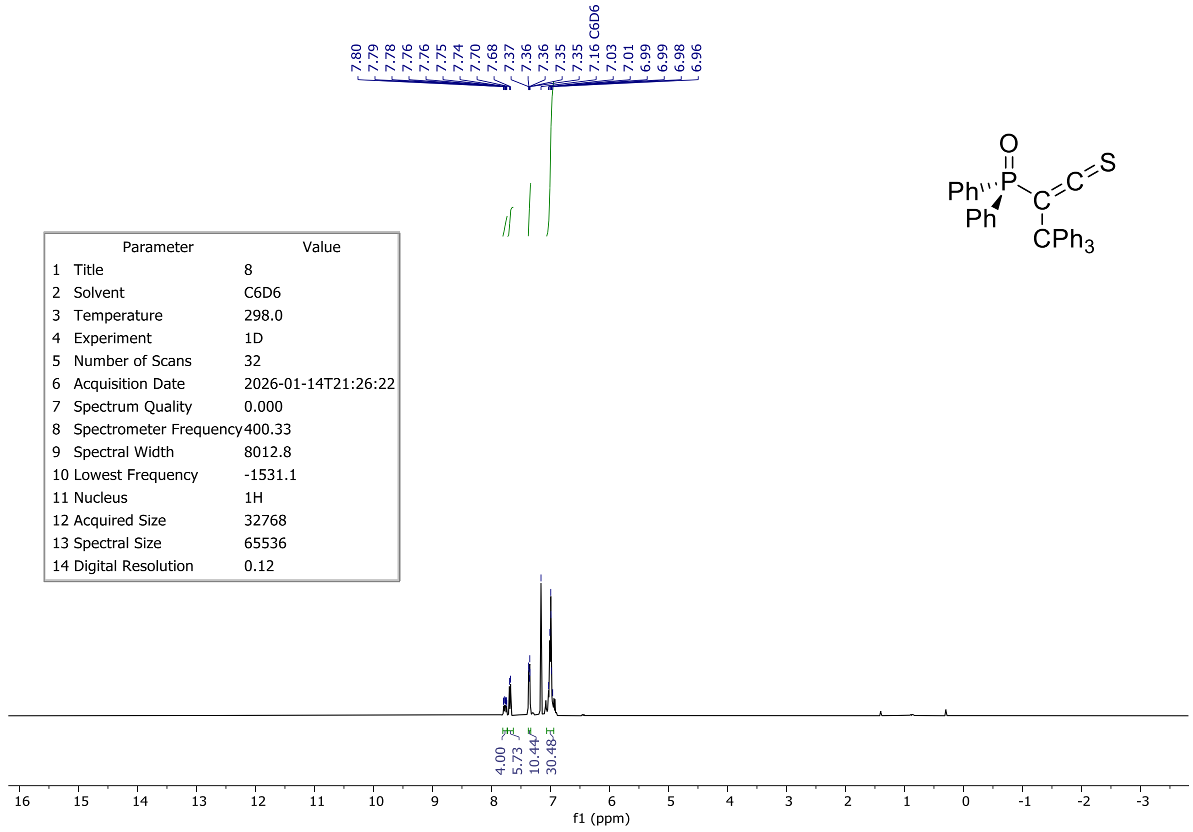

Figure S45 ^1H NMR spectrum of compound **8** + CPh3Cl in C_6D_6. $\mathbf{^1H}$**-NMR** (400 MHz, C_6D_6): δ = {peaks at 7.37 – 7.35 (m, 10H) and 7.03 – 6.96 (m, 15H) ppm corresponds to trityl chloride} 7.81 – 7.72 (m, 4H, C*H*$_{P\text{-}Ph,\mathit{ortho}}$), 7.70 – 7.68 (m, 6H, C*H*$_{C\text{-}Ph,\mathit{ortho}}$), 7.03 – 6.96 (m, 15H, C*H*$_{P\text{-}Ph,\mathit{meta,para}}$ + C*H*$_{C\text{-}Ph,\mathit{meta,para}}$) ppm.

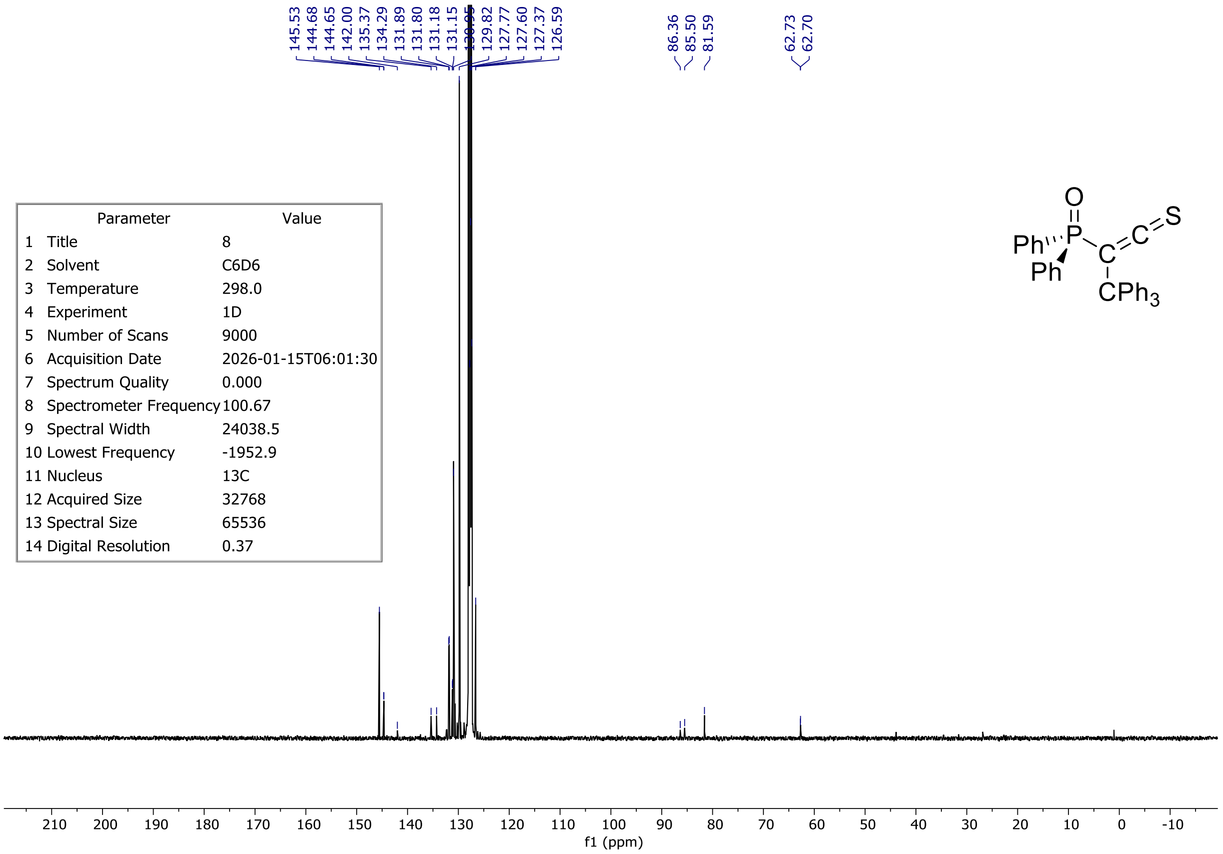

Figure S46 $^{13}C\{^1H\}$ NMR spectrum of compound **8** + CPh_3Cl in C_6D_6. **$^{13}C\{^1H\}$-NMR** (101 MHz, C_6D_6): δ = {peaks at 145.90, 130.19, 128.02, 127.96 and 81.96 ppm corresponds to trityl chloride} 145.03 (d, $^3J_{CP}$ = 3.1 Hz, $C_{C\text{-Ph},ipso}$), 142.37 (s, PC*C*S), 135.20 (d, $^1J_{CP}$ = 109.1 Hz, $C_{P\text{-Ph},ipso}$), 132.21 (d, $^2J_{CP}$ = 9.4 Hz, $CH_{P\text{-Ph},ortho}$), 131.53 (d, $^4J_{CP}$ = 3.0 Hz, $CH_{P\text{-Ph},para}$), 131.32 (s, $CH_{C\text{-Ph},ortho}$), 128.07 (d, $^3J_{CP}$ = 12.2 Hz, $CH_{P\text{-Ph},meta}$), 127.74 (s, $CH_{C\text{-Ph},meta}$), 126.96 (s, $CH_{C\text{-Ph},para}$), 86.30 (d, $^1J_{CP}$ = 87.0 Hz, P*C*CS), 63.08 (d, $^2J_{CP}$ = 2.9 Hz, CPh_3) ppm.

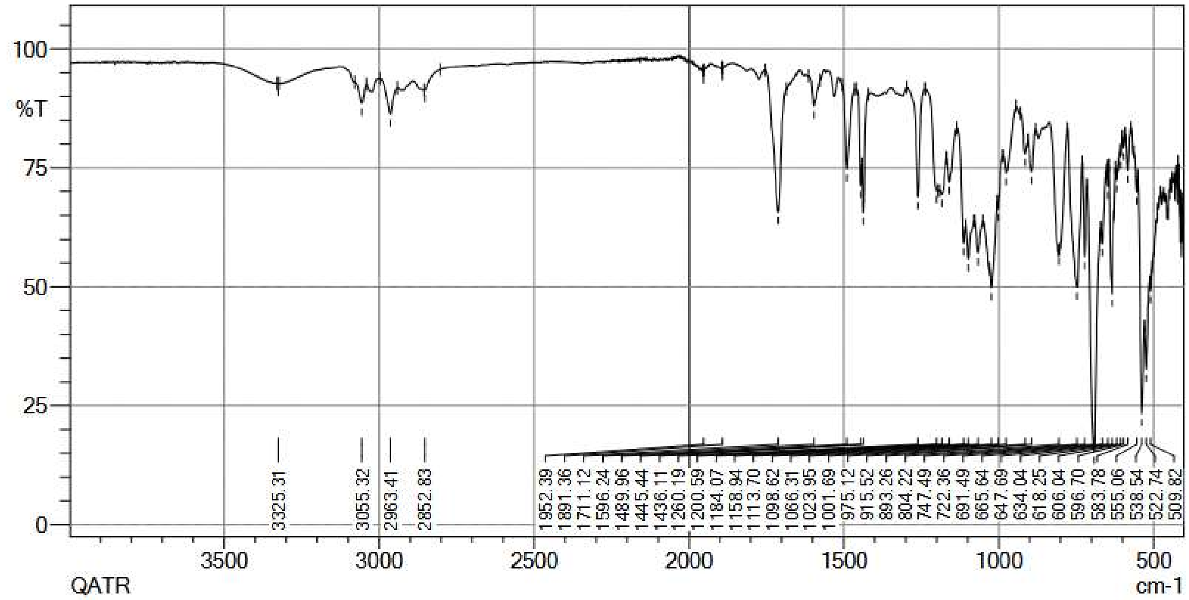

Figure S47 IR spectrum of compound **8** (solid state).

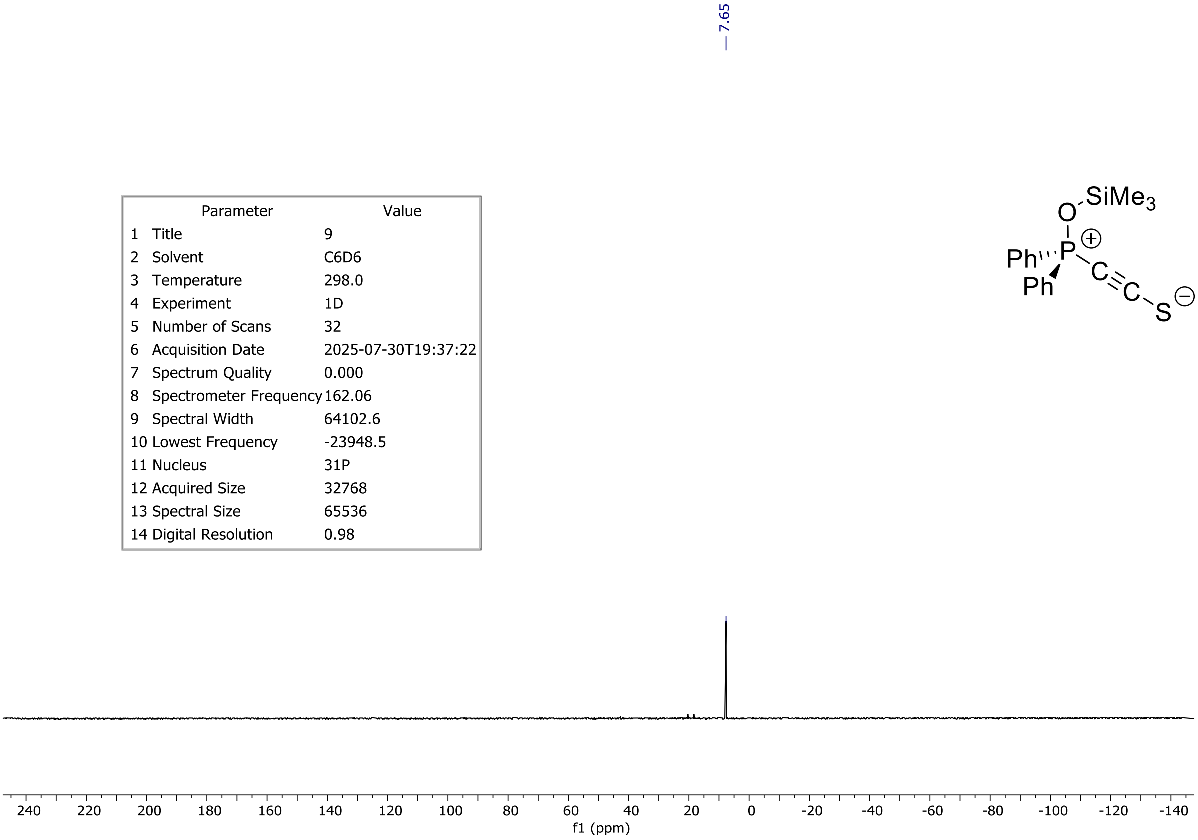

	Parameter	Value
1	Title	9
2	Solvent	C6D6
3	Temperature	298.0
4	Experiment	1D
5	Number of Scans	32
6	Acquisition Date	2025-07-30T19:37:22
7	Spectrum Quality	0.000
8	Spectrometer Frequency	162.06
9	Spectral Width	64102.6
10	Lowest Frequency	-23948.5
11	Nucleus	31P
12	Acquired Size	32768
13	Spectral Size	65536
14	Digital Resolution	0.98

Figure S48 ^{31}P{^{1}H} NMR spectrum of compound **9** in C_6D_6. **^{31}P{^{1}H}-NMR** (162 MHz, C_6D_6): δ = 7.65 (s, *P*Ph_2O) ppm.

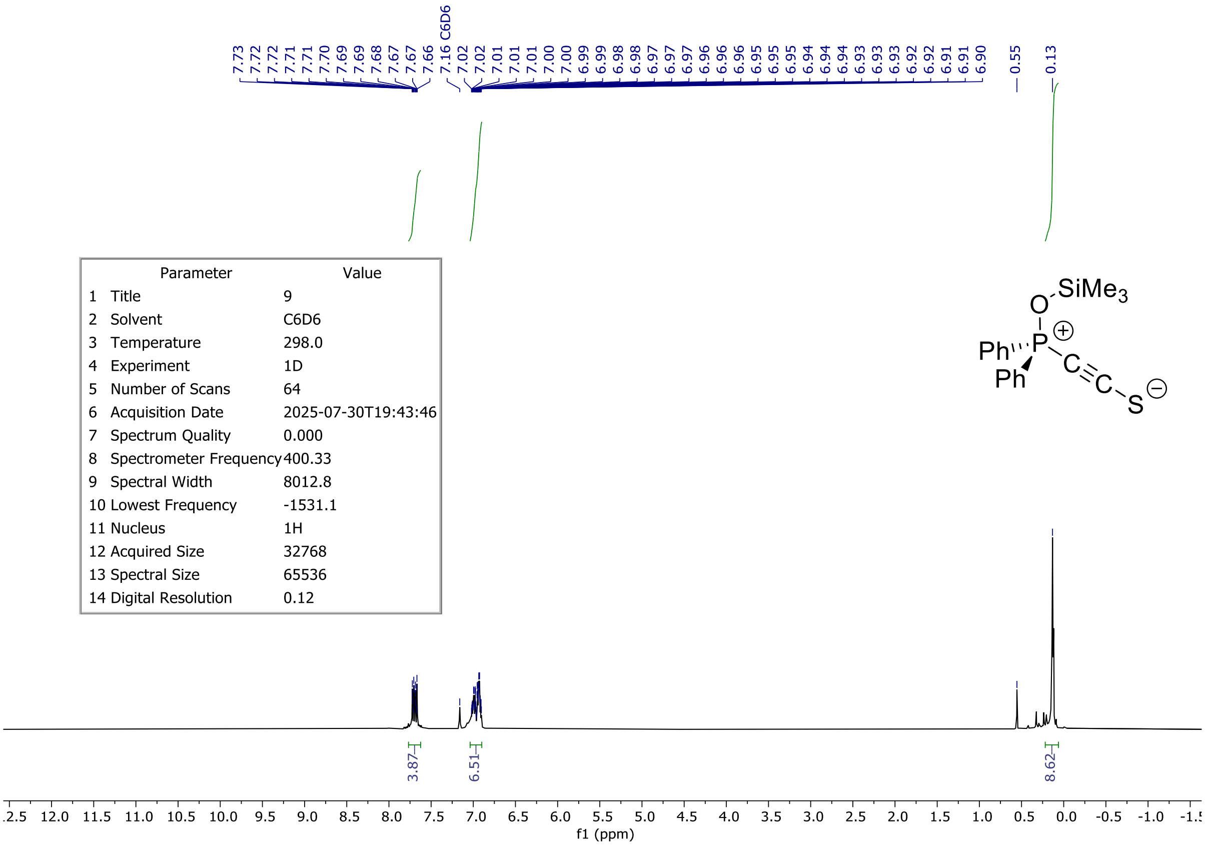

	Parameter	Value
1	Title	9
2	Solvent	C6D6
3	Temperature	298.0
4	Experiment	1D
5	Number of Scans	64
6	Acquisition Date	2025-07-30T19:43:46
7	Spectrum Quality	0.000
8	Spectrometer Frequency	400.33
9	Spectral Width	8012.8
10	Lowest Frequency	-1531.1
11	Nucleus	1H
12	Acquired Size	32768
13	Spectral Size	65536
14	Digital Resolution	0.12

Figure S49 ^{1}H NMR spectrum of compound **9** in C_6D_6. **^{1}H-NMR** (400 MHz, C_6D_6): δ = 7.73 – 7.66 (m, 4H, C*H*$_{Ph,\textit{ortho}}$), 7.02 – 6.90 (m, 6H, C*H*$_{Ph,\textit{meta,para}}$), 0.13 (s, 9H, C*H*$_3$) ppm.

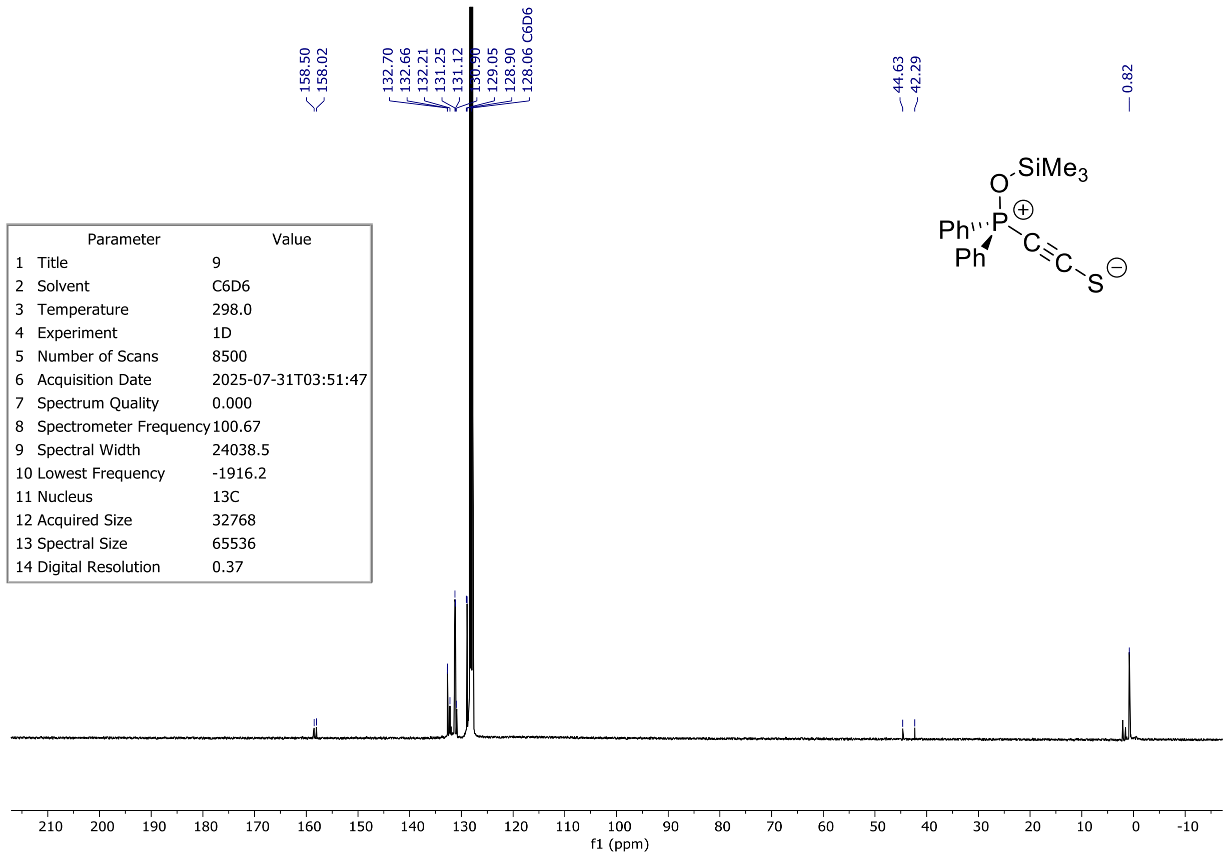

	Parameter	Value
1	Title	9
2	Solvent	C6D6
3	Temperature	298.0
4	Experiment	1D
5	Number of Scans	8500
6	Acquisition Date	2025-07-31T03:51:47
7	Spectrum Quality	0.000
8	Spectrometer Frequency	100.67
9	Spectral Width	24038.5
10	Lowest Frequency	-1916.2
11	Nucleus	13C
12	Acquired Size	32768
13	Spectral Size	65536
14	Digital Resolution	0.37

Figure S50 $^{13}C\{^1H\}$ NMR spectrum of compound **9** in C_6D_6. **^{13}C{^{1}H}-NMR** (101 MHz, C_6D_6): δ = 158.26 (d, $^2J_{CP}$ = 48.2 Hz, PC*C*S), 132.68 (d, $^4J_{CP}$ = 3.1 Hz, *C*H$_{Ph,para}$), 131.56 (d, $^1J_{CP}$ = 132.68 Hz, *C*$_{Ph,ipso}$), 131.18 (d, $^2J_{CP}$ = 12.6 Hz, *C*H$_{Ph,ortho}$), 128.97 (d, $^3J_{CP}$ = 14.3 Hz, *C*H$_{Ph,meta}$), 43.46 (d, $^1J_{CP}$ = 235.2 Hz, PCCS), 0.82 (s, *C*H$_3$) ppm.

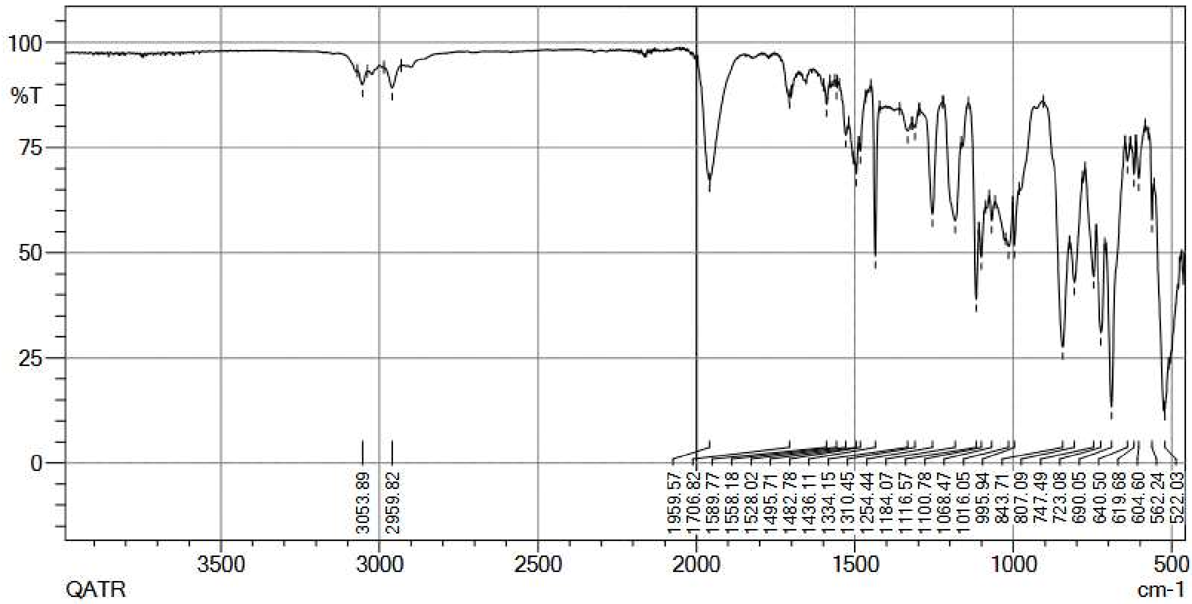

Figure S51 IR spectrum of compound **9** (solid state).

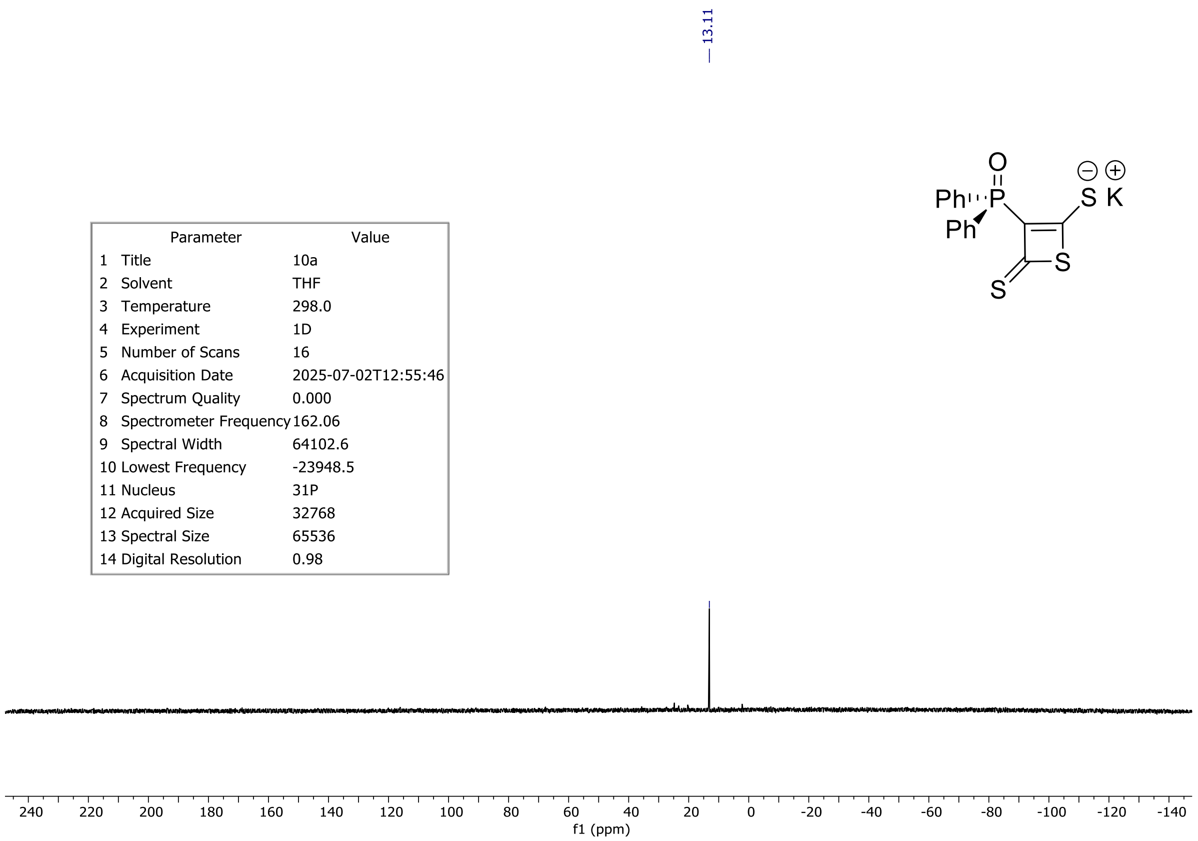

	Parameter	Value
1	Title	10a
2	Solvent	THF
3	Temperature	298.0
4	Experiment	1D
5	Number of Scans	16
6	Acquisition Date	2025-07-02T12:55:46
7	Spectrum Quality	0.000
8	Spectrometer Frequency	162.06
9	Spectral Width	64102.6
10	Lowest Frequency	-23948.5
11	Nucleus	31P
12	Acquired Size	32768
13	Spectral Size	65536
14	Digital Resolution	0.98

Figure S52 $^{31}P\{^{1}H\}$ NMR spectrum of compound **10a** in THF-d8. **$^{31}P\{^{1}H\}$-NMR** (162 MHz, THF-d_8): δ = 13.11 (s, *P*Ph_2O) ppm.

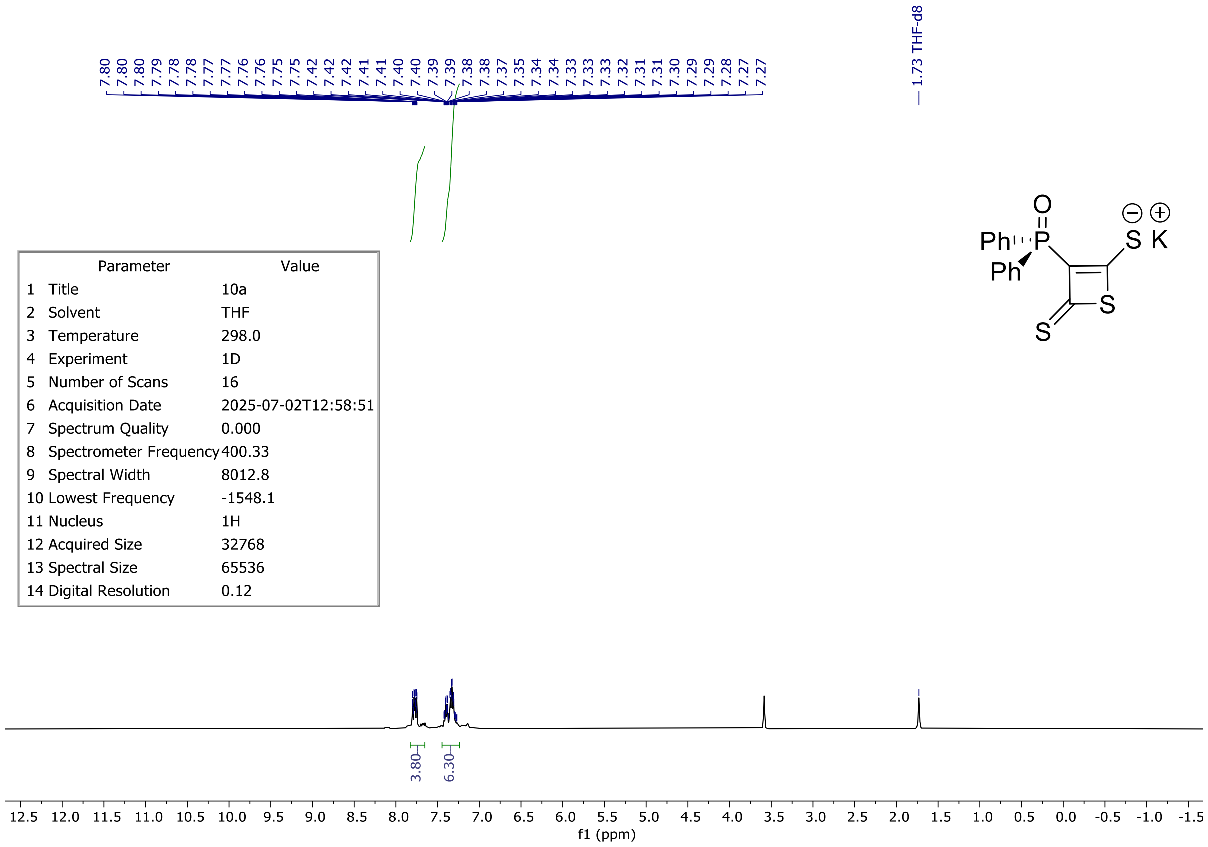

	Parameter	Value
1	Title	10a
2	Solvent	THF
3	Temperature	298.0
4	Experiment	1D
5	Number of Scans	16
6	Acquisition Date	2025-07-02T12:58:51
7	Spectrum Quality	0.000
8	Spectrometer Frequency	400.33
9	Spectral Width	8012.8
10	Lowest Frequency	-1548.1
11	Nucleus	1H
12	Acquired Size	32768
13	Spectral Size	65536
14	Digital Resolution	0.12

Figure S53 ^{1}H NMR spectrum of compound **10a** in THF-d8. **^{1}H-NMR** (400 MHz, THF-d_8): δ = 7.80 – 7.75 (m, 4H, C$H_{Ph,ortho}$), 7.42 – 7.27 (m, 6H, C$H_{Ph,meta,para}$) ppm.

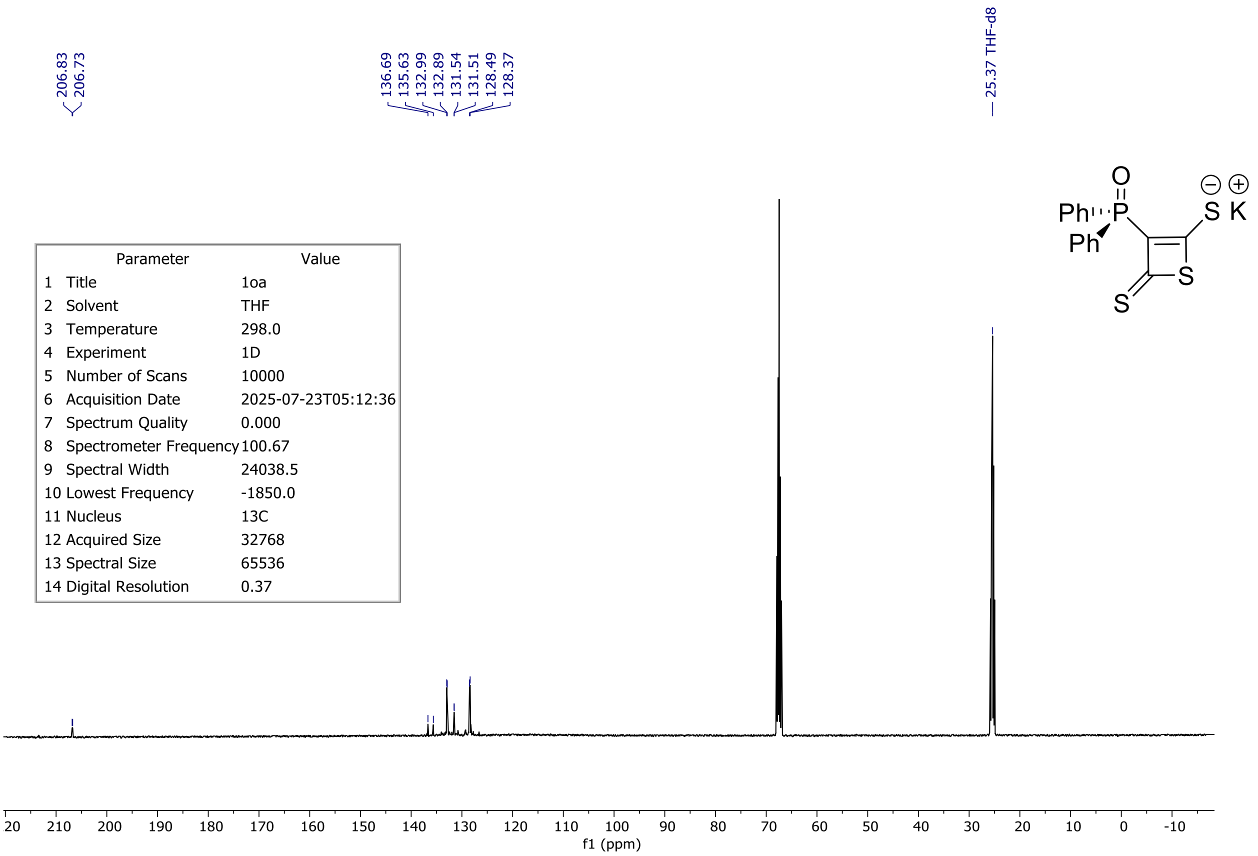

	Parameter	Value
1	Title	1oa
2	Solvent	THF
3	Temperature	298.0
4	Experiment	1D
5	Number of Scans	10000
6	Acquisition Date	2025-07-23T05:12:36
7	Spectrum Quality	0.000
8	Spectrometer Frequency	100.67
9	Spectral Width	24038.5
10	Lowest Frequency	-1850.0
11	Nucleus	13C
12	Acquired Size	32768
13	Spectral Size	65536
14	Digital Resolution	0.37

Figure S54 $^{13}C\{^1H\}$ NMR spectrum of compound **10a** in THF-d8. **^{13}C{^{1}H}-NMR** (101 MHz, THF-d_8): δ = 206.78 (d, $^2J_{CP}$ = 10.0 Hz, PC*C*S), 136.16 (d, $^1J_{CP}$ = 107.0 Hz, *C*$_{Ph,ipso}$), 132.94 (d, $^2J_{CP}$ = 10.6 Hz, *C*H$_{Ph,ortho}$), 131.53 (d, $^4J_{CP}$ = 2.8 Hz, *C*H$_{Ph,para}$), 128.43 (d, $^3J_{CP}$ = 12.2 Hz, *C*H$_{Ph,meta}$), not observed (P*C*CS) ppm.

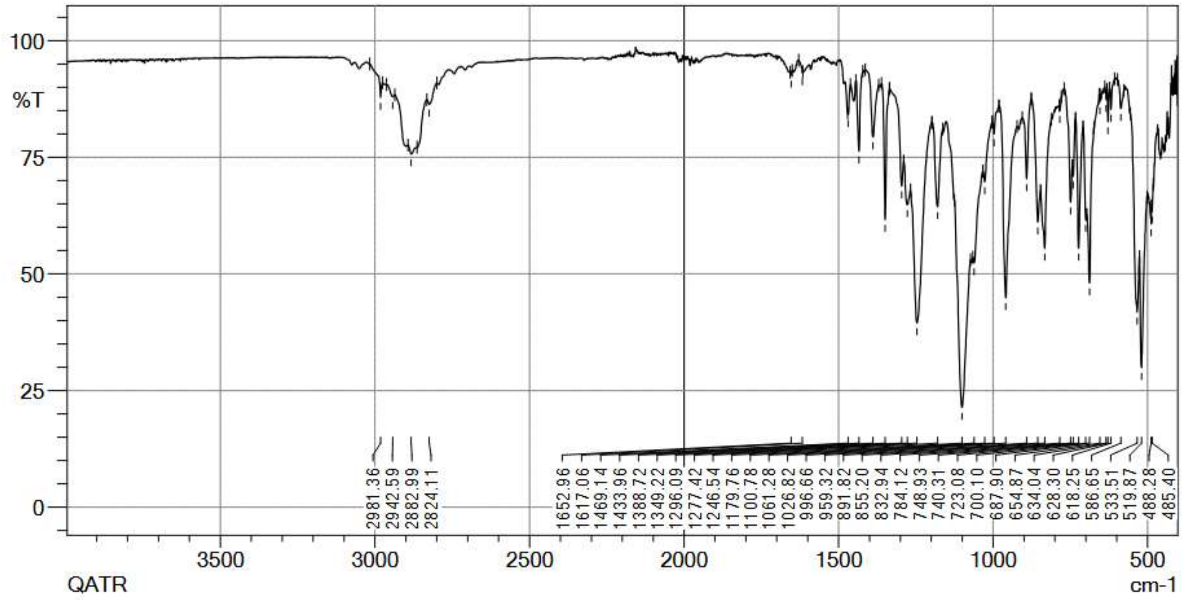

Figure S55 IR spectrum of compound **10a** (solid state).

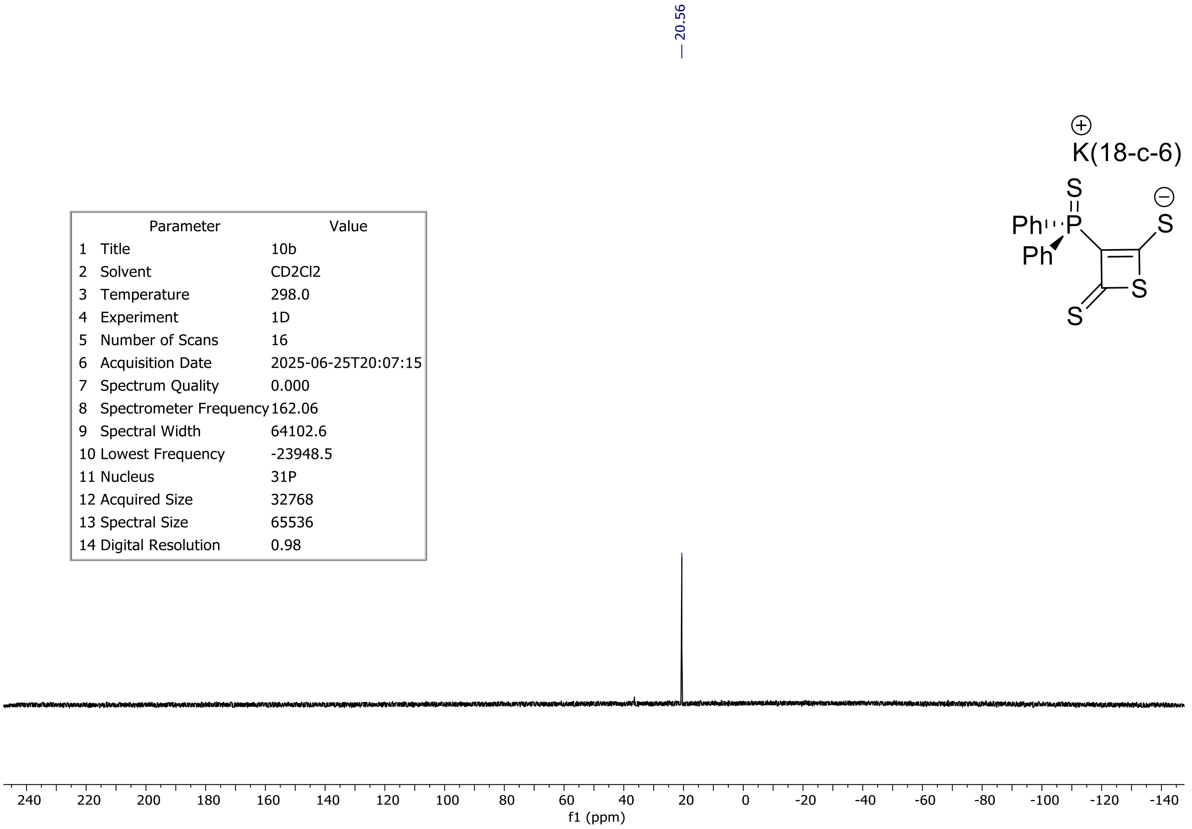

Figure S56 $^{31}P\{^1H\}$ NMR spectrum of compound **10b** in CD_2Cl_2. $\mathbf{^{31}P\{^1H\}}$**-NMR** (162 MHz, CD_2Cl_2): δ = 20.56 (s, *P*Ph_2S) ppm.

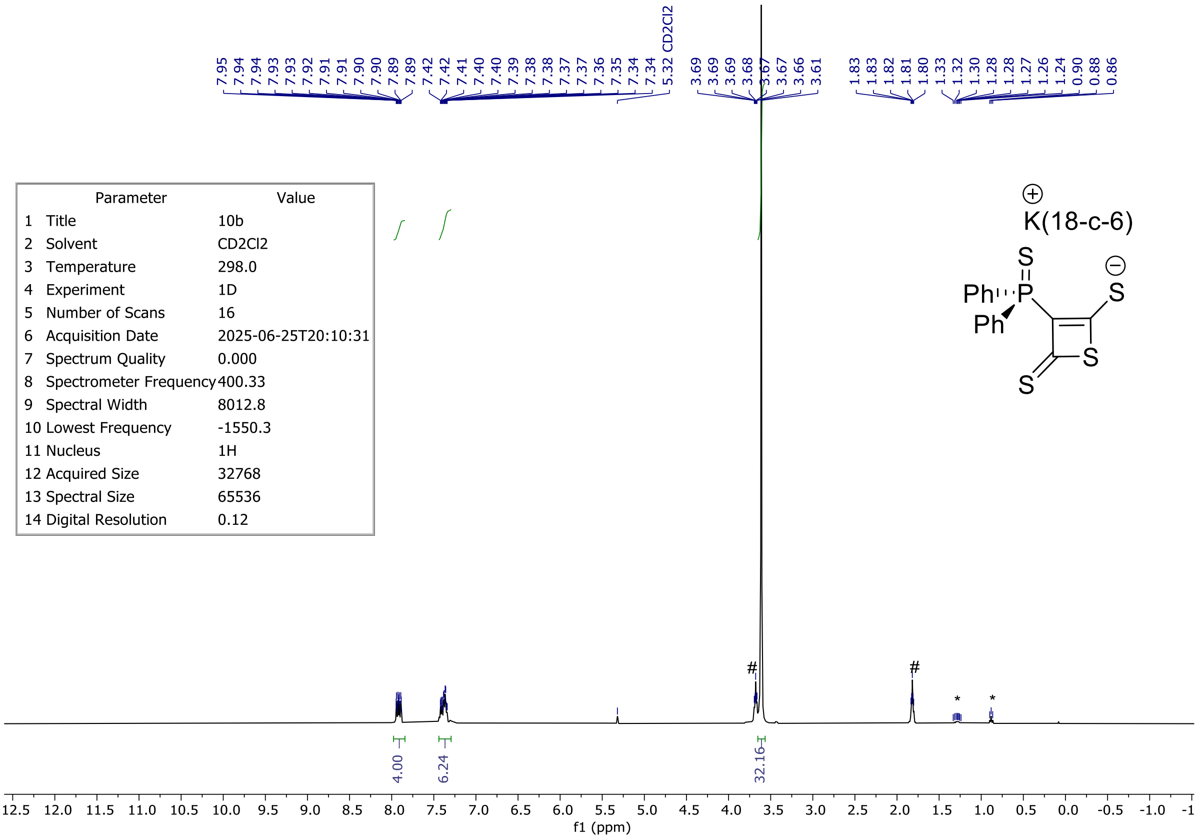

Figure S57 ^{1}H NMR spectrum of compound **10b** in CD_2Cl_2. **^{1}H-NMR** (400 MHz, CD_2Cl_2): δ = 7.95 – 7.89 (m, 4H, $CH_{Ph,ortho}$), 7.42 – 7.34 (m, 6H, $CH_{Ph,meta,para}$), 3.61 (s, 32H, $CH_{2,crown}$)ppm. The peaks at 1.31 ppm and 0.89 ppm correspond to residual pentane and the peaks at 3.69 ppm and 1.82 ppm correspond to residual THF.

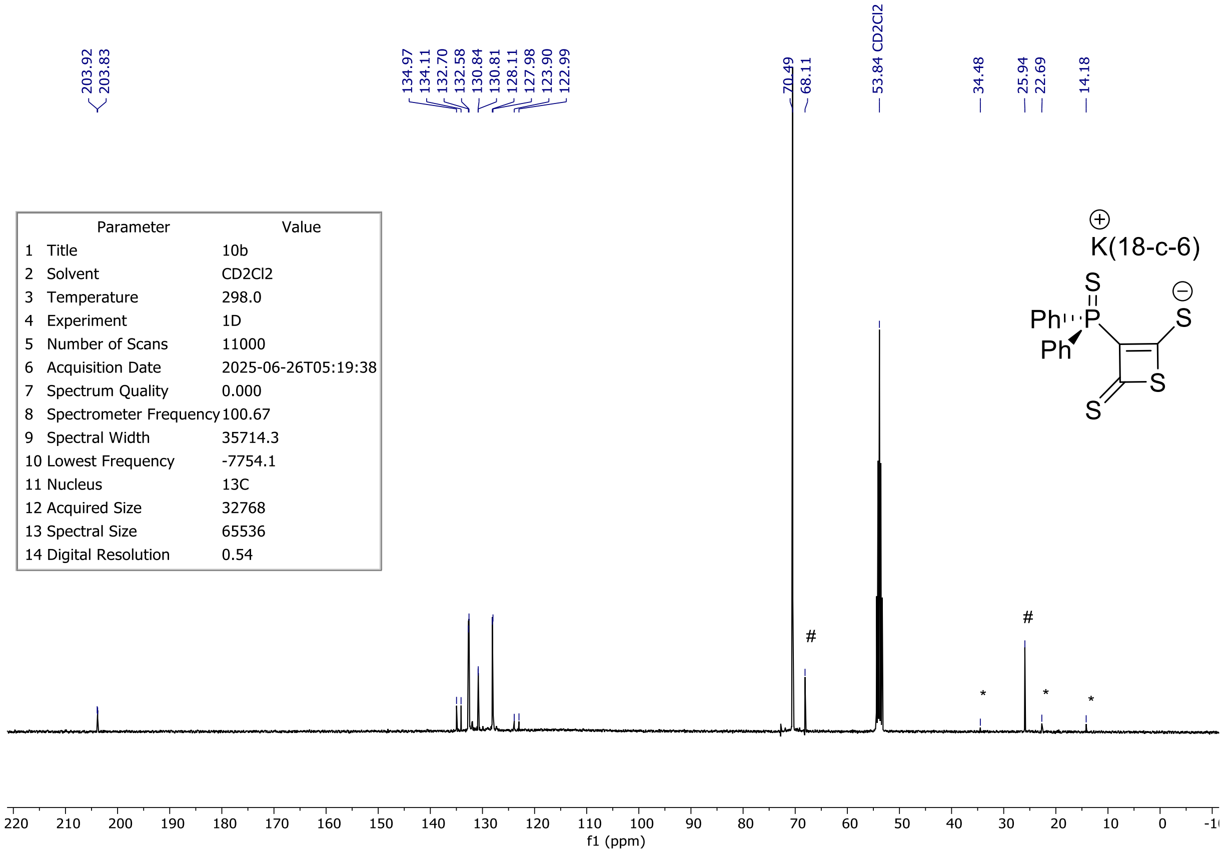

Figure S58 $^{13}C\{^{1}H\}$ NMR spectrum of compound **10b** in CD_2Cl_2. **$^{13}C\{^{1}H\}$-NMR** (101 MHz, CD_2Cl_2): δ = 203.92(d, $^{2}J_{CP}$ = 9.3 Hz, PC*C*S), 134.54 (d, $^{1}J_{CP}$ = 87.2 Hz, $C_{Ph,ipso}$), 132.64 (d, $^{2}J_{CP}$ = 11.4 Hz, $CH_{Ph,ortho}$), 130.82 (d, $^{4}J_{CP}$ = 3.1 Hz, $CH_{Ph,para}$), 128.04 (d, $^{3}J_{CP}$ = 12.6 Hz, $CH_{Ph,meta}$), 123.45 (d, $^{1}J_{CP}$ = 91.5 Hz, P*C*CS) ppm. The peaks at 34.48 ppm, 22.69 ppm and 14.18 ppm correspond to residual pentane and the peaks at 68.11 ppm and 25.94 ppm correspond to residual THF.

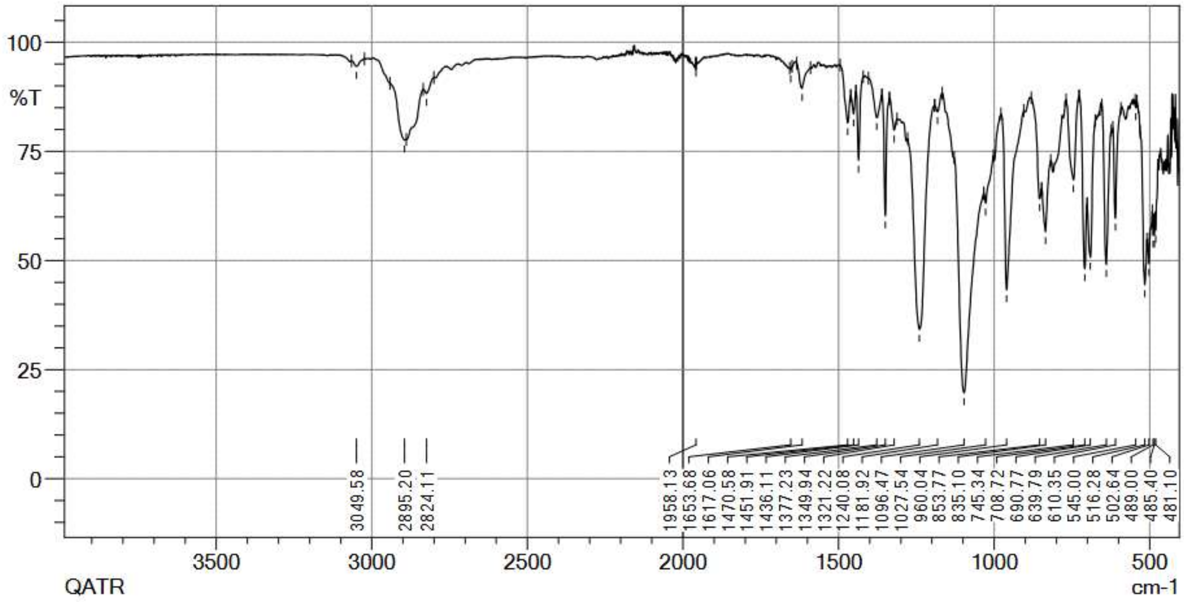

Figure S59 IR spectrum of compound **10b** (solid state).

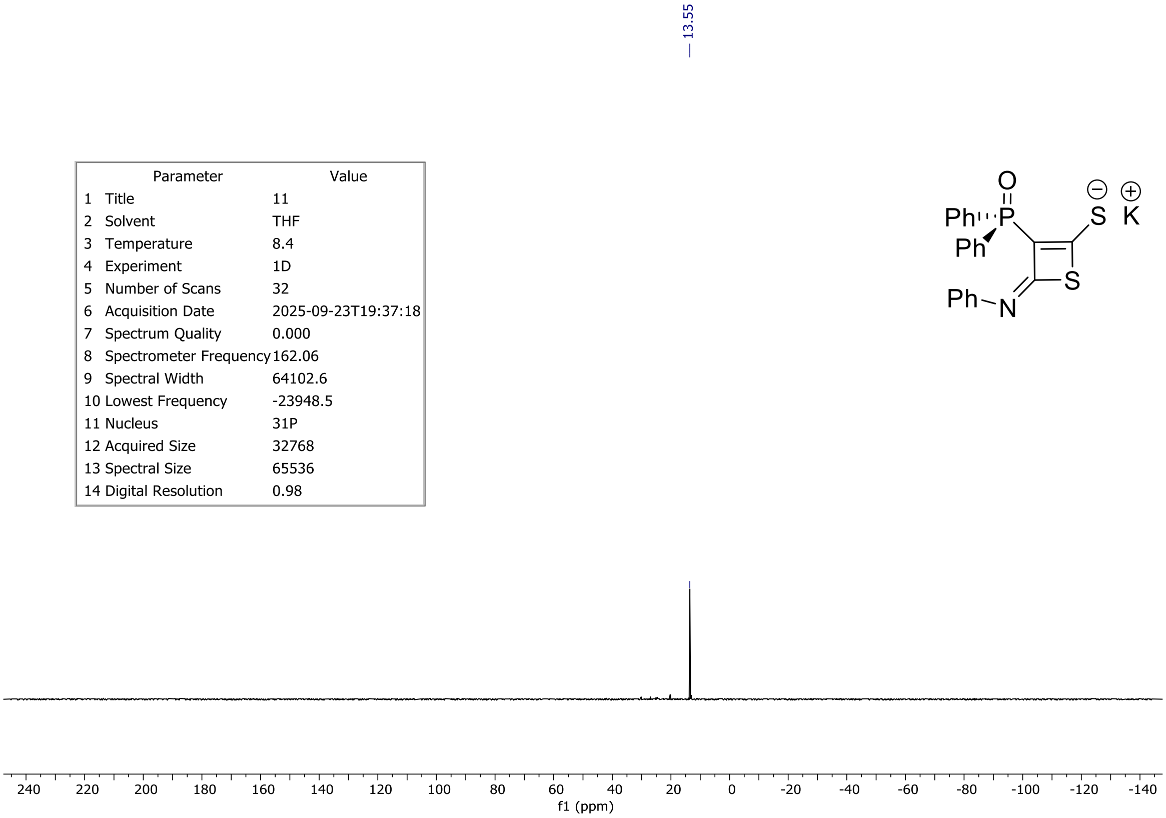

	Parameter	Value
1	Title	11
2	Solvent	THF
3	Temperature	8.4
4	Experiment	1D
5	Number of Scans	32
6	Acquisition Date	2025-09-23T19:37:18
7	Spectrum Quality	0.000
8	Spectrometer Frequency	162.06
9	Spectral Width	64102.6
10	Lowest Frequency	-23948.5
11	Nucleus	31P
12	Acquired Size	32768
13	Spectral Size	65536
14	Digital Resolution	0.98

Figure S60 $^{31}P\{^1H\}$ NMR spectrum of compound **11** in THF-d8. **$^{31}P\{^1H\}$-NMR** (162 MHz, THF-d_8): δ = 13.55 (s, *P*Ph_2O) ppm.

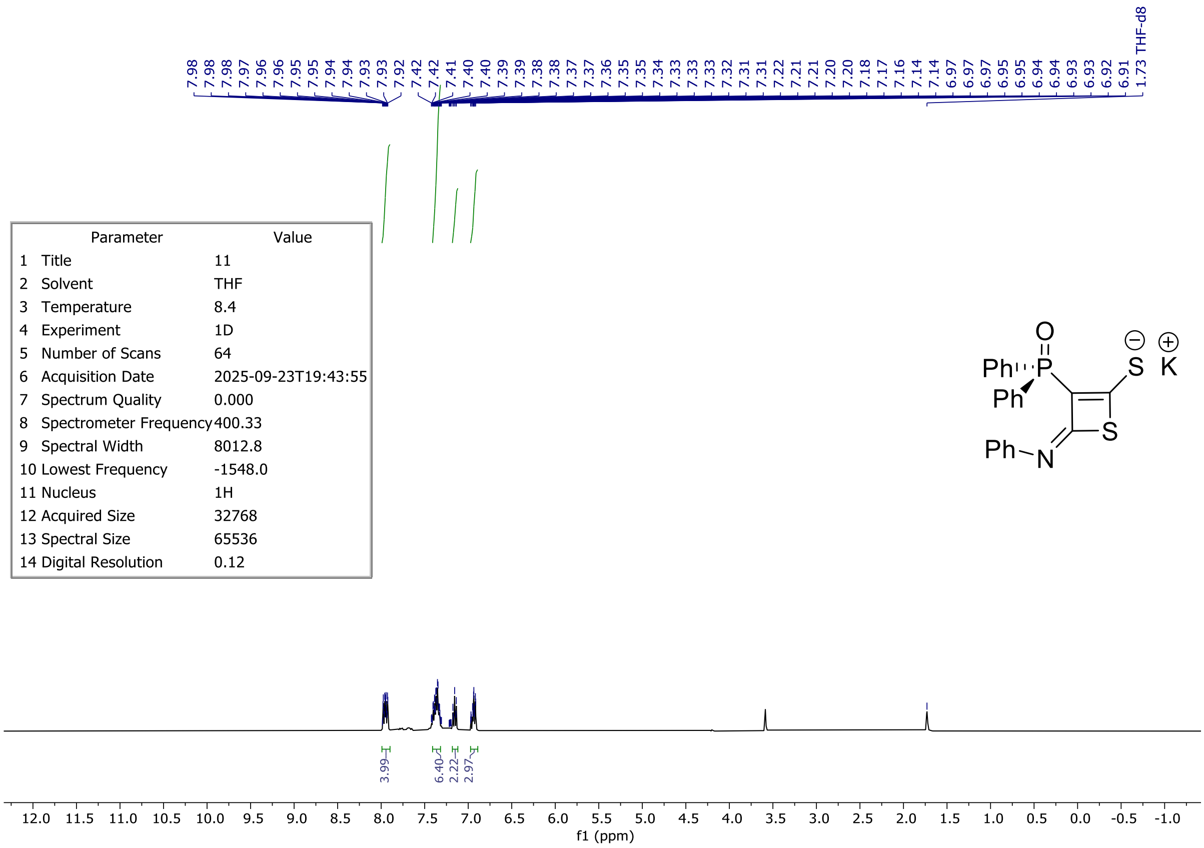

	Parameter	Value
1	Title	11
2	Solvent	THF
3	Temperature	8.4
4	Experiment	1D
5	Number of Scans	64
6	Acquisition Date	2025-09-23T19:43:55
7	Spectrum Quality	0.000
8	Spectrometer Frequency	400.33
9	Spectral Width	8012.8
10	Lowest Frequency	-1548.0
11	Nucleus	1H
12	Acquired Size	32768
13	Spectral Size	65536
14	Digital Resolution	0.12

Figure S61 ^1H NMR spectrum of compound **11** in THF-d8. **^1H-NMR** (400 MHz, THF-d_8): δ = 7.98 – 7.92 (m, 4H, PC$H_{Ph,ortho}$), 7.42 – 7.31 (m, 6H, PC$H_{Ph,meta,para}$), 7.16 (t, $^3J_{HH}$ = 7.8 Hz, 2H, NC$H_{Ph,ortho}$), 6.97 – 6.91 (m, 3H, NC$H_{Ph,meta,para}$), ppm.

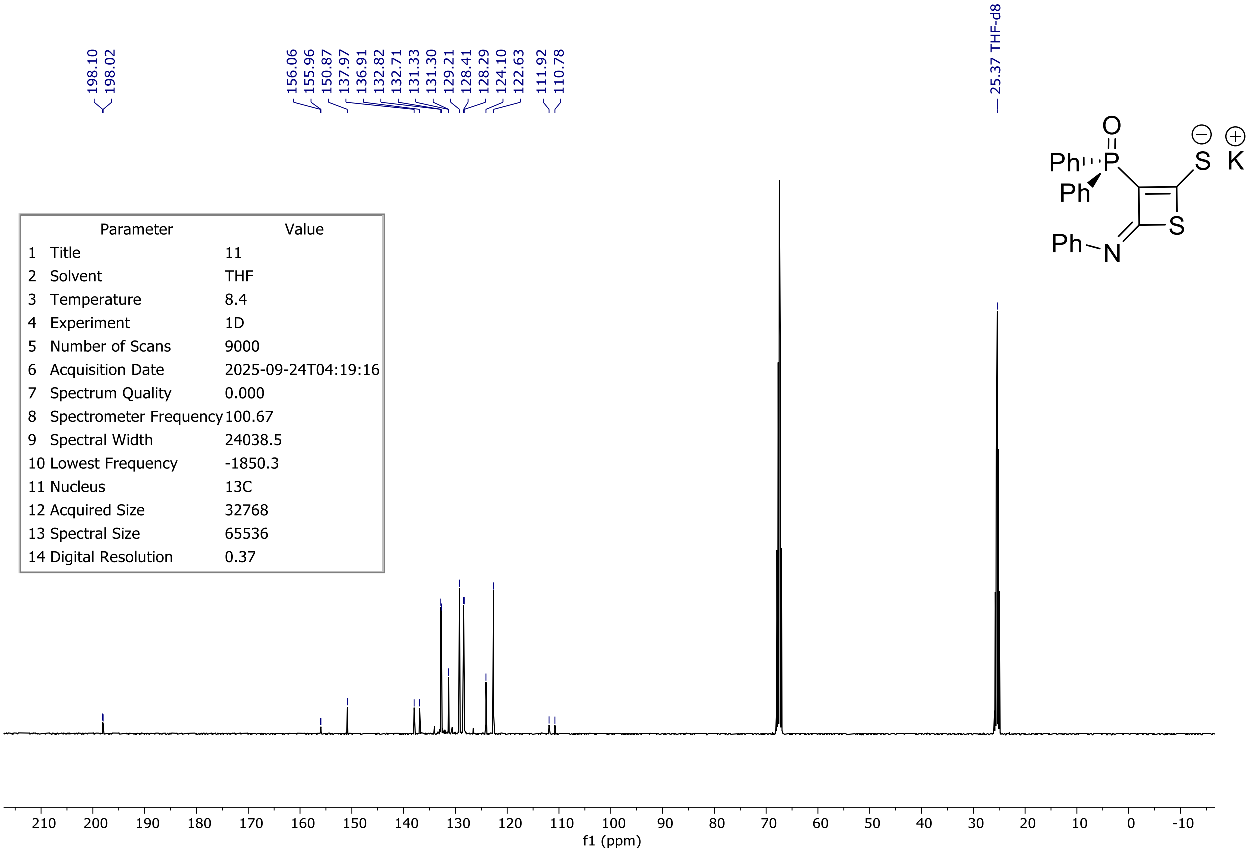

Figure S62 $^{13}C\{^1H\}$ NMR spectrum of compound **11** in THF-d_8. **^{13}C{^{1}H}-NMR** (101 MHz, THF-d_8): δ = 198.06 (d, $^2J_{CP}$ = 8.6 Hz, PC*C*S), 156.01 (d, $^2J_{CP}$ = 10.3 Hz, PC*C*N), 150.87 (s, N*C*$_{Ph,ipso}$), 137.44 (d, $^1J_{CP}$ = 105.9 Hz, P*C*$_{Ph,ipso}$), 132.77 (d, $^2J_{CP}$ = 10.8 Hz, P*C*H$_{Ph,ortho}$), 131.31 (d, $^4J_{CP}$ = 2.9 Hz, P*C*H$_{Ph,para}$),129.21 (s, N*C*H$_{Ph,ortho}$), 128.35 (d, $^3J_{CP}$ = 12.1 Hz, P*C*H$_{Ph,meta}$), 124.10 (s, N*C*H$_{Ph,para}$), 122.63 (s, N*C*H$_{Ph,meta}$), 111.35 (d, $^1J_{CP}$ = 113.8 Hz, P*C*CS) ppm.

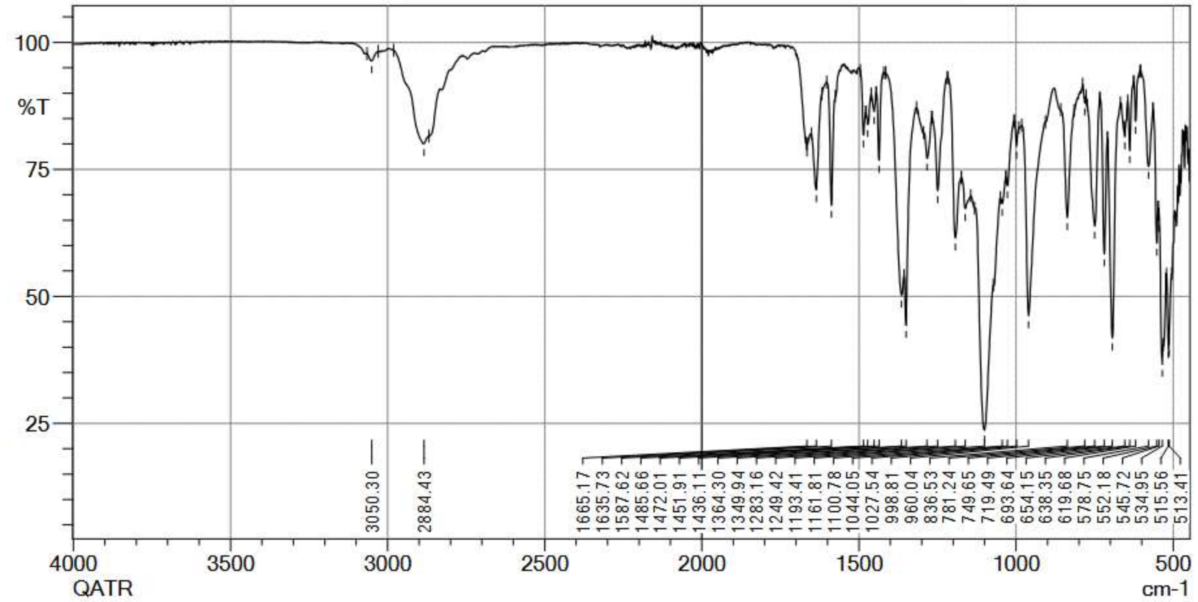

Figure S63 IR spectrum of compound **11** (solid state).

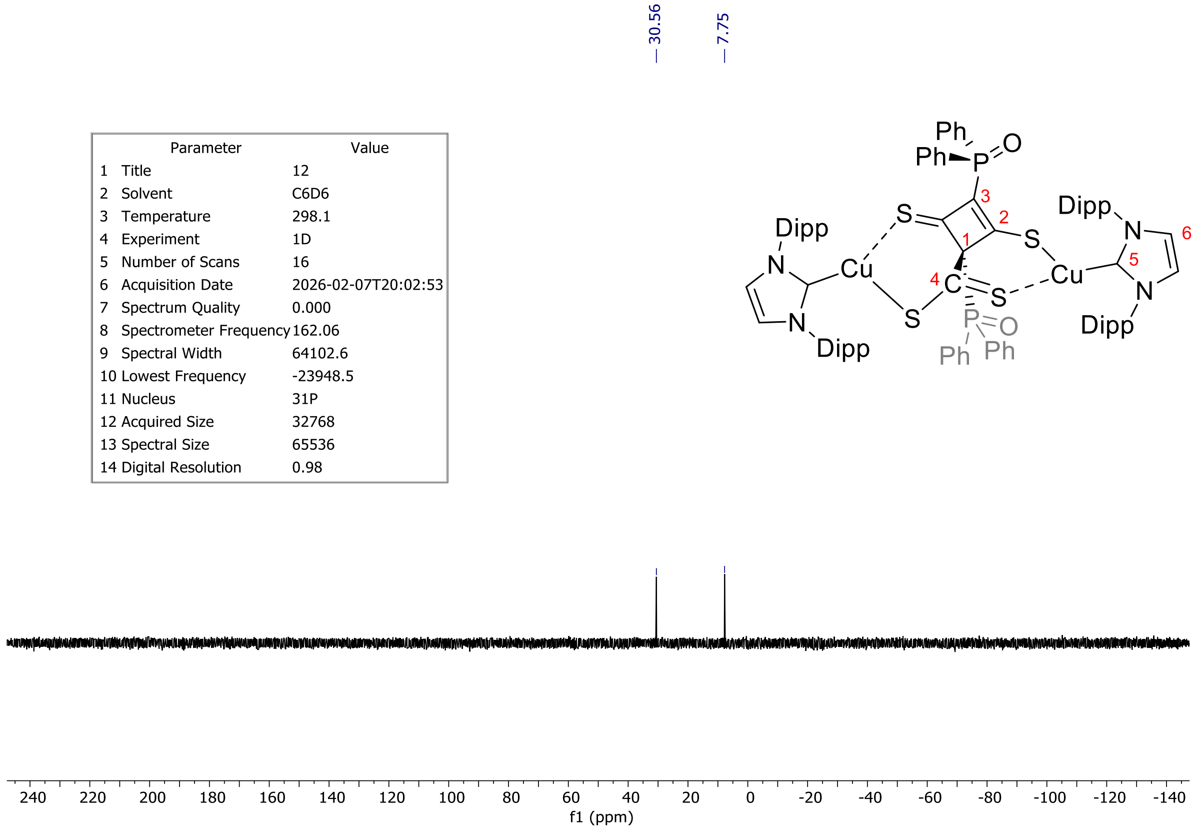

	Parameter	Value
1	Title	12
2	Solvent	C6D6
3	Temperature	298.1
4	Experiment	1D
5	Number of Scans	16
6	Acquisition Date	2026-02-07T20:02:53
7	Spectrum Quality	0.000
8	Spectrometer Frequency	162.06
9	Spectral Width	64102.6
10	Lowest Frequency	-23948.5
11	Nucleus	31P
12	Acquired Size	32768
13	Spectral Size	65536
14	Digital Resolution	0.98

Figure S64 $^{31}P\{^{1}H\}$ NMR spectrum of compound **12** in C_6D_6. **$^{31}P\{^{1}H\}$-NMR** (162 MHz, C_6D_6): δ = 30.56 (s, *P*Ph_2O), 7.75 (s, *P*Ph_2O) ppm.

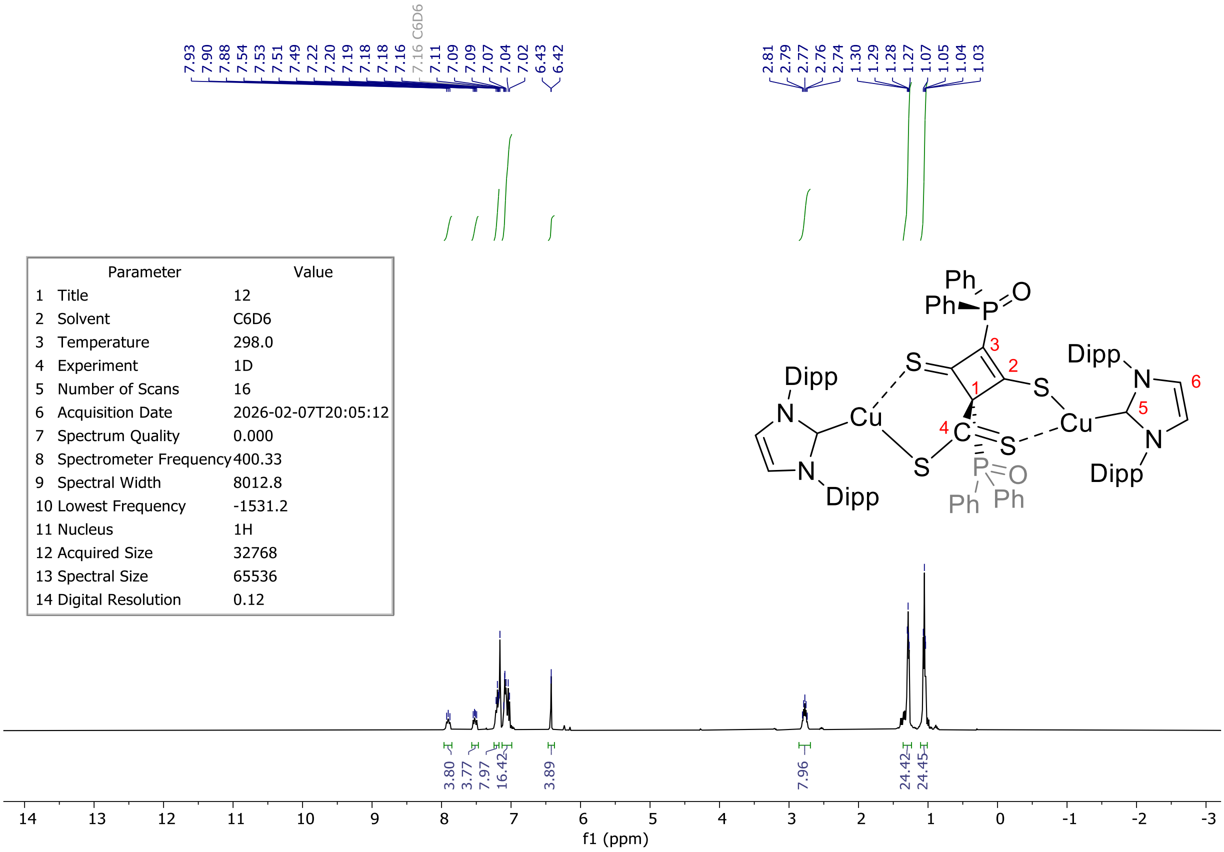

Parameter	Value
1 Title	12
2 Solvent	C6D6
3 Temperature	298.0
4 Experiment	1D
5 Number of Scans	16
6 Acquisition Date	2026-02-07T20:05:12
7 Spectrum Quality	0.000
8 Spectrometer Frequency	400.33
9 Spectral Width	8012.8
10 Lowest Frequency	-1531.2
11 Nucleus	1H
12 Acquired Size	32768
13 Spectral Size	65536
14 Digital Resolution	0.12

Figure S65 ^{1}H NMR spectrum of compound **12** in C_6D_6. **^{1}H-NMR** (400 MHz, C_6D_6): δ = 7.93 – 7.88 (m, 4H, C$H_{Ph,ortho}$), 7.54 – 7.49 (m, 4H, C$H_{Ph,ortho}$), 7.22 – 7.18 (m, 4H, C$H_{Dipp,para}$ + m, 4H, C$H_{Ph,para}$), 7.11 – 7.02 (m, 8H, C$H_{Ph,meta}$ + m, 8H, C$H_{Dipp,meta}$), 6.43 (s, 4H, C6*H*), 2.77 (septet, 8H, $^{3}J_{HH}$ = 6.9 Hz, C*H*(CH_3)$_2$), 1.30 – 1.27 (m, 24H, CH_3), 1.07 – 1.03 (m, 24H, CH_3)ppm.

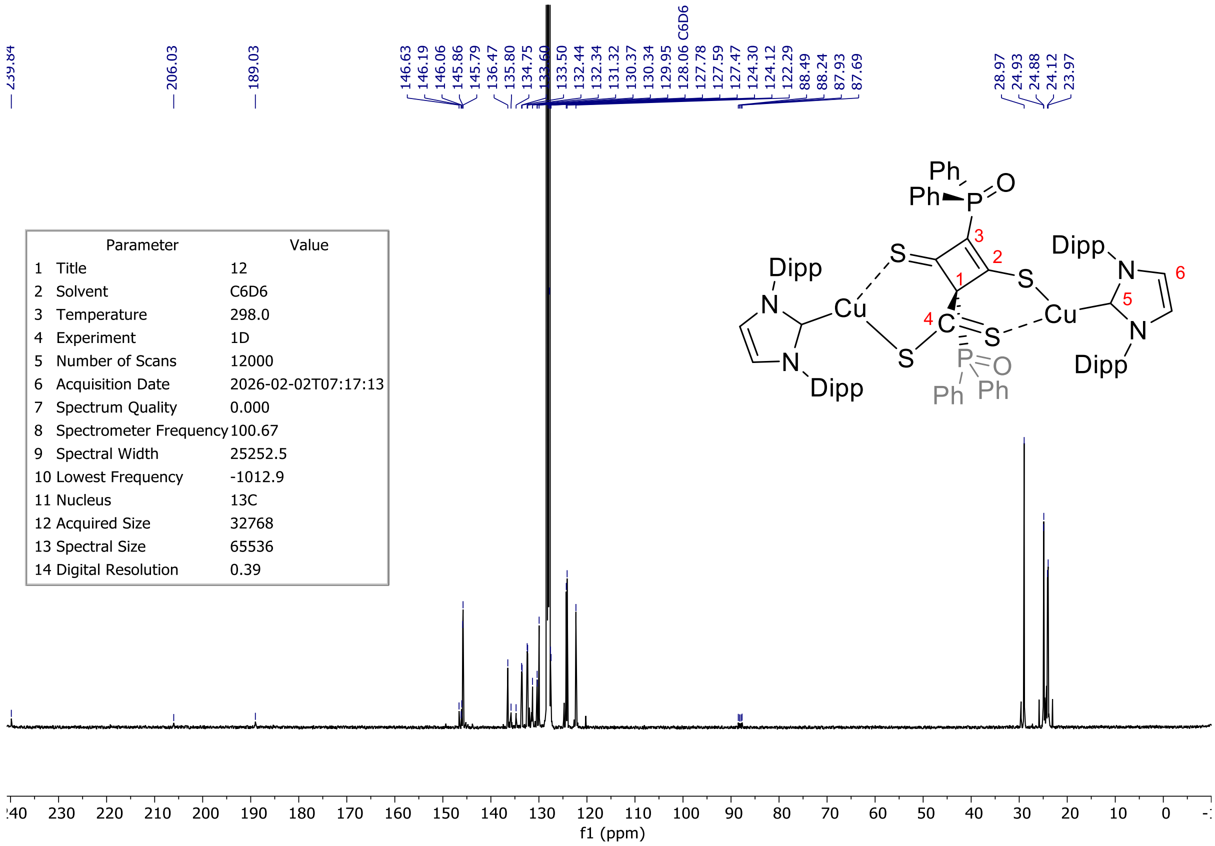

Figure S66 $^{13}C\{^1H\}$ NMR spectrum of compound **12** in C_6D_6. **$^{13}C\{^1H\}$-NMR** (101 MHz, C_6D_6): δ = 239.84 (s, *C*4), 206.03 (s, *C*2), 189.03 (s, *C*5), 146.34 (d, $^1J_{CP}$ = 56.9 Hz, $C_{Ph,ipso}$), 145.86 (s, $CH_{Dipp,ortho}$), 145.79 (s, $CH_{Dipp,ortho}$), 136.47(s, $C_{Dipp,ipso}$), 135.27 (d, $^1J_{CP}$ = 105.6 Hz, $C_{Ph,ipso}$), 133.55 (d, $^2J_{CP}$ = 9.9 Hz, $CH_{Ph,ortho}$), 132.39 (d, $^2J_{CP}$ = 10.1 Hz, $CH_{Ph,ortho}$), 131.32 (s, $CH_{Ph,para}$), 130.37 (s, $CH_{Ph,para}$), 129.95 (s, $CH_{Dipp,para}$), 127.53 (d, $^3J_{CP}$ = 12.3 Hz, $CH_{Ph,meta}$), 124.35 (d, $^3J_{CP}$ = 9.6 Hz, $CH_{Ph,meta}$), 124.30 (s, $CH_{Dipp,meta}$), 124.12 (s, $CH_{Dipp,meta}$), 122.29 (s, *C*6), 88.09 (dd, $^1J_{CP}$ = 55.9 Hz, $^3J_{CP}$ = 24.7 Hz, *C*3), 28.97 (s, $CH(CH_3)_2$), 24.93 (s, CH_3), 24.88 (s, CH_3), 24.12 (s, CH_3), 23.97 (s, CH_3), not observed (*C*1) ppm.

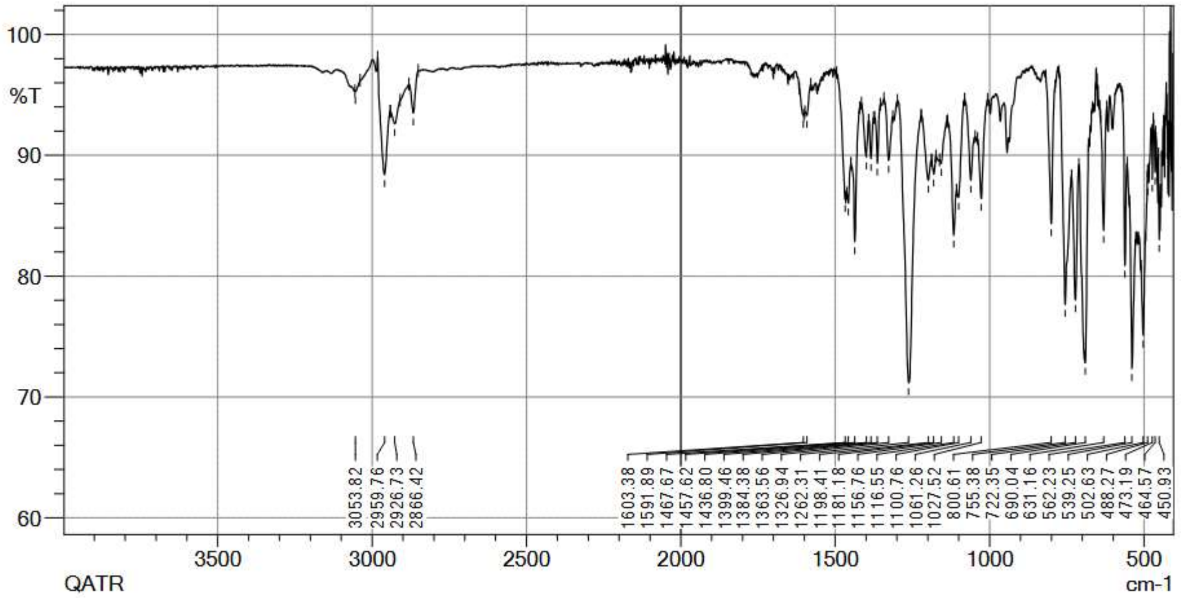

Figure S67 IR spectrum of compound **12** (solid state).

3. Real-Time IR Spectroscopy

General information

A two-neck Schlenk tube was equipped with an IR SiComp probe, and the immersion depth was adjusted using a Teflon QuickFit adapter. After recording a background spectrum, a 10 mL THF solution of **1**^{PO} was introduced via the side arm. The solution was cooled to 0 °C and this temperature was maintained throughout the reaction. Data acquisition was initiated using the software controlling the ReactIR spectrometer, and spectra were recorded every 30 s. At this stage, compound **1**^{PO} was observed with a band at 2092 cm^{-1}, corresponding to the CCO stretching vibration.

After a few spectra had been recorded, one equivalent of CS2 was added and the reaction was monitored. The signal of **1**^{PO} was gradually consumed and a new band appeared at 1732 cm-1. This new species was identified as compound **3**^{PO}, and the band at 1732 cm-1 was assigned to the CO stretching vibration. Subsequently, another compound appeared, characterized by two bands at 1964 and 2022 cm-1, concomitantly with the decrease of the signal corresponding to **3**^{PO}. This new species was identified as compound **2**^{PO}.

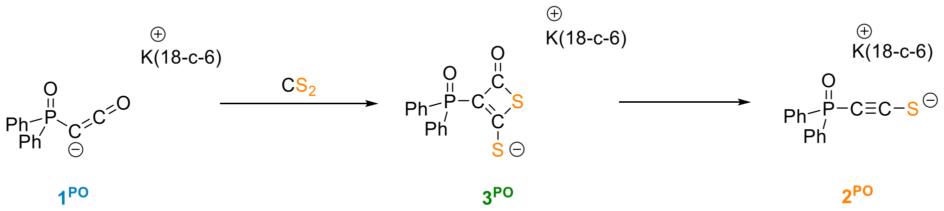

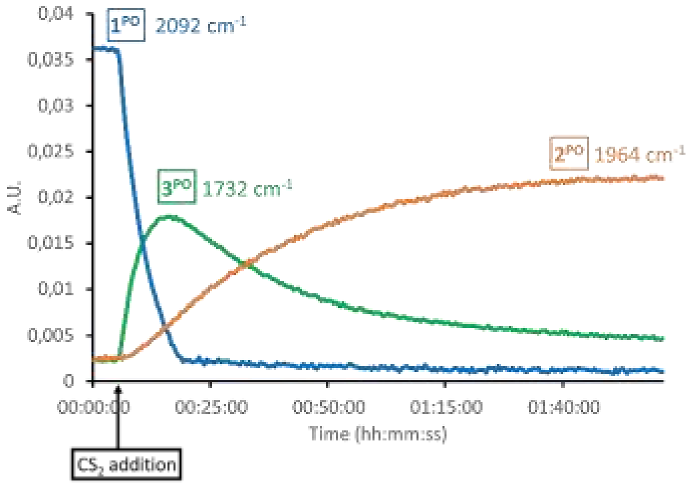

Figure S68 Kinetic plot of the formation of **2**^{PO} from **1**^{PO} and CS_2.

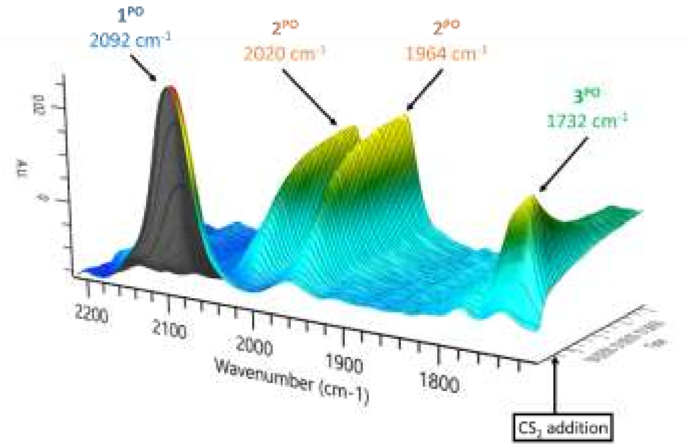

Figure S69 View of the surface plot of the formation of $\mathbf{2^{PO}}$ from $\mathbf{1^{PO}}$ and CS_2.

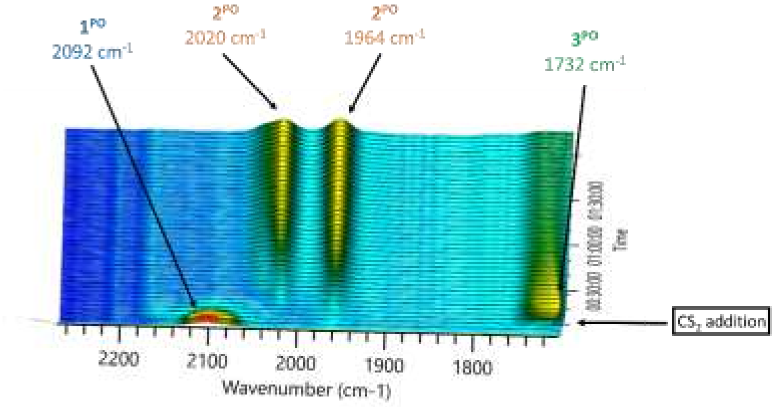

Figure S70 Top view of the surface plot of the formation of $\mathbf{2^{PO}}$ from $\mathbf{1^{PO}}$ and CS_2.

4. Crystal structure determination

4.1. General information

High-quality single crystals of appropriate dimensions were placed in an inert oil such as perfluoropolyalkylether, hand-picked under polarized optical microscopy and then mounted on the diffractometer. The data collection was done at 100 K. X-ray intensity data measurements of all compounds were carried out on an Oxford SuperNova diffractometer with graphite-monochromatized (CuK_{α}= 1.54184 Å) radiation. The X-ray generator was operated at 50 kV and 30 mA.

All structures were solved by intrinsic phasing and refined by the full-matrix least-squares on F^2 using software package and expanded using Fourier techniques.[4–7] Non-hydrogen atoms were refined anisotropically, while all hydrogen atoms were placed on the ideal positions using riding models and refined isotropically with displacement parameters constrained to those of the parent atoms (1.5 times Ueq for methyl groups and 1.2 times Ueq for all other carbon bound H).

Data collection and structure refinement details for all compounds are given in the following tables. Further details on the structure refinement are provided in the following sections for each individual structure.

Crystallographic data including structure factors have been deposited with the Cambridge Crystallographic Data Centre as supplementary publication no. CCDC - 2528269 to CCDC - 2528277 Copies of the data can be gained free of charge on application to Cambridge Crystallographic Data Centre, 12 Union Road, Cambridge CB2 1EZ, UK; [fax: (+44) 1223-336-033; email: deposit@ccdc.cam.ac.uk].

Table S1 Data collection and structure refinement details for compounds **4a**^{PO}; **2**^{PO} and **2**^{PS}

**Compound**	**4a**^{PO}	**2**^{PO}	**2**^{PS}
Formula	$C_{33}H_{39}KNO_8PS$	$C_{56}H_{76}K_2O_{15}P_2S_2$	$C_{26}H_{34}KO_6PS_2$
CCDC	2528273	2528269	2528270
Formula weight	679.78	1193.42	576.72
Temperature [K]	100(2)	100(2)	100(2)
Wave lenght [Å]	1.54184	1.54184	1.54184
Crystal system	Monoclinic	Monoclinic	Monoclinic
Space group	*P2₁/n*	*I2/a*	*P2₁/n*
a [Å]	12.92220(10)	20.5342(2)	8.69530(10)
b [Å]	14.17050(10)	14.81560(10)	17.9592(2)
c [Å]	18.81260(10)	19.97590(10	18.4090(2)
α [°]	90	90	90
β [°]	101.6710(10)	95.7480(10)	96.1080(10)
γ [°]	90	90	90
Volumen [Å^3]	3373.63(4)	6046.64(8)	2858.44(6)
Z	4	4	4
Calc. density [Mg·m^{-3}]	1.338	1.311	1.340
μ ($Mo_{K\alpha}$) [mm^{-1}]	2.827	3.053	3.835
F(000)	1432	2528	1216
Crystal dimensions [mm]	0.290 x 0.210 x 0.120	0.380 x 0.150 x 0.100	0.290 x 0.190 x 0.130
Theta range θ [°]	3.817 to 76.789	3.685 to 76.716	3.448 to 76.877
Index ranges	-15<=h<=16,<br>-17<=k<=17,<br>-23<=l<=22	-24<=h<=25,<br>-18<=k<=18,<br>-25<=l<=22	-9<=h<=10,<br>-21<=k<=22,<br>-22<=l<=23
Reflections collected	46549	41838	37510
Independent reflections	6983 [R(int) = 0.0322]	6203 [R(int) = 0.0249]	5882 [R(int) = 0.0338]
Data/Restraints/Parameter	6983 / 0 / 406	6203 / 0 / 370	5882 / 0 / 325
Goodness-of-fit on F^2	1.060	1.033	1.068
Final R indices [I>2sigma(I)]	R1 = 0.0292, wR2 = 0.0760	R1 = 0.0300, wR2 = 0.0808	R1 = 0.0279, wR2 = 0.0744
Largest diff. peak and hole [e·Å-3]	0.413 and -0.269	0.472 and -0.344	0.397 and -0.376

Table S2 Data collection and structure refinement details for compounds **2**[Ts], **3**[Ts] and **4**[Ts]

**Compound**	**2**[Ts]	**3**[Ts]	**4**[Ts]
Formula	$C_{25}H_{39}NaO_{10}S_2$	$C_{27}H_{41}Cl_2NaO_{11}S_3$	$C_{34}H_{43}KNO_7P$
CCDC	2528271	2528272	2528274
Formula weight	586.67	731.67	647.76
Temperature [K]	100(2)	100(2)	100(2)
Wave lenght [Å]	1.54184	1.5418	1.54184
Crystal system	Monoclinic	Monoclinic	Triclinic
Space group	*P2₁/c*	*P2₁/n*	*P-1*
a [Å]	13.0776(4)	15.9540(3)	10.3759(3)
b [Å]	15.3505(3)	11.4507(2)	12.3450(4)
c [Å]	15.3494(4)	18.4128(3)	14.6301(5)
α [°]	90	90	111.650(3)
β [°]	111.430(3)	91.333(2)°.	96.913(2)
γ [°]	90	90	99.598(2)
Volumen [$Å^3$]	2868.32(14)	3362.82(10)	1683.23(10)
Z	4	4	2
Calc. density [$Mg·m^{-3}$]	1.359	1.445	1.278
μ ($Mo_{K\alpha}$) [mm^{-1}]	2.286	4.083	2.218
F(000)	1248	1536	688
Crystal dimensions [mm]	0.439 x 0.193 x 0.054	0.442 x 0.204 x 0.114	0.340 x 0.140 x 0.100
Theta range θ [°]	3.631 to 76.687	3.624 to 76.993	3.316 to 67.078
Index ranges	-16<=h<=15,<br>-19<=k<=19,<br>-16<=l<=19	-20<=h<=20,<br>-13<=k<=14,<br>-21<=l<=23	-12<=h<=12<br>-14<=k<=14<br>-16<=l<=17
Reflections collected	5836	44041	19773
Independent reflections	5836 [R(int) = 0.1191]	6926 [R(int) = 0.0835]	6023 [R(int) = 0.0297]
Data/Restraints/Parameter	5836 / 0 / 345	6926 / 213 / 570	6023 / 0 / 399
Goodness-of-fit on F^2	1.254	1.046	1.046
Final R indices [I>2sigma(I)]	R1 = 0.0804, wR2 = 0.2470	R1 = 0.0594, wR2 = 0.1609	1 = 0.0333, wR2 = 0.0938
Largest diff. peak and hole [e·Å-3]	0.699 and -0.725	0.794 and -0.650	0.477 and -0.385

Table S3 Data collection and structure refinement details for compounds **8, 9** and **12**

**Compound**	**9**	**8**	**12**
Formula	$C_{17}H_{19}OPSSi$	$C_{39}H_{31}OPS$	$C_{86}H_{95}Cu_2N_4O_2P_2S_4$
CCDC	2528275	2528276	2528277
Formula weight	330.44	578.67	1533.91
Temperature [K]	100(2)	100(2)	100(2)
Wave lenght [Å]	1.54184	1.54184	1.54184
Crystal system	Triclinic	Monoclinic	Triclinic
Space group	*P-1*	*C2/c*	*P-1*
a [Å]	9.3963(2)	24.3183(2)	14.9350(2)
b [Å]	9.6673(2	8.28650(10)	17.8946(2)
c [Å]	9.8843(2)	30.2403(3)	18.9167(2)
α [°]	95.731(2)	90	112.8290(10)
β [°]	93.096(2)	96.1070(10)	109.2100(10)
γ [°]	104.320(2)	90	95.8350(10)
Volumen [$Å^3$]	862.72(3)	6059.25(11)	4244.64(9)
Z	2	8	2
Calc. density [$Mg \cdot m^{-3}$]	1.272	1.269	1.200
μ ($Mo_{K\alpha}$) [mm^{-1}]	3.168	1.674	2.246
F(000)	348	2432	1614
Crystal dimensions [mm]	0.330 x 0.240 x 0.190	0.320 x 0.120 x 0.100	0.270 x 0.180 x 0.090
Theta range θ [°]	4.511 to 76.796	2.939 to 76.789.	2.768 to 76.757
Index ranges	-11<=h<=11,	-30<=h<=30,	-18<=h<=18
	-11<=k<=12,	-10<=k<=10	-22<=k<=22
	-11<=l<=7	-25<=l<=37	-23<=l<=21
Reflections collected	9718	37796	56609
Independent reflections	3395 [R(int) = 0.0279]	6282 [R(int) = 0.0248]	17132 [R(int) = 0.0334]
Data/Restraints/Parameter	3395 / 0 / 193	6282 / 0 / 379	17132 / 0 / 917
Goodness-of-fit on F^2	1.049	1.028	1.019
Final R indices [I>2sigma(I)]	R1 = 0.0287, wR2 = 0.0775	R1 = 0.0310, wR2 = 0.0751	R1 = 0.0381, wR2 = 0.1004
Largest diff. peak and hole [e·Å-3]	0.370 and -0.344	0.374 and -0.426	0.960 and -0.468

Table S4 Data collection and structure refinement details for compounds **3^{PS}**, **10a** and **11**

**Compound**	**3^{PS}**	**11**	**10a**
Formula	$C_{27}H_{34}KO_7PS_3$	$C_{33}H_{39}KNO_7PS_2$	$C_{27}H_{34}KO_7PS_3$
CCDC			
Formula weight	636.79	695.84	636.79
Temperature [K]	100(2)	100(2)	100(2)
Wave lenght [Å]	1.54184	1.54184	1.54184
Crystal system	Monoclinic	Monoclinic	Triclinic
Space group	*P2₁/n*	*C2/c*	*P 21/c*
a [Å]	9.1263(2)	23.76730(10)	16.0687(3)
b [Å]	16.7335(3)	15.00430(10)	9.3354(2)
c [Å]	20.0231(4)	38.6631(2)	20.4788(4)
α [°]	90	90	90
β [°]	95.140(2)	97.0000(10)	91.607(2)
γ [°]	90	90	90
Volumen [$Å^3$]	3045.53(11)	13684.94(13)	3070.77(11)
Z	4	16	4
Calc. density [$Mg\cdot m^{-3}$]	1.389	1.351	1.377
μ ($Mo_{K\alpha}$) [mm^{-1}]	4.304	3.334	4.268
F(000)	1336	5856	1336
Crystal dimensions [mm]	0.182 x 0.154 x 0.101	0.490 x 0.220 x 0.130	0.500 x 0.410 x 0.150
Theta range θ [°]	3.448 to 77.127	3.491 to 76.779	2.751 to 76.905
Index ranges	-10<=h<=11,	-30<=h<=29,	-14 <= h <= 20,
	-21<=k<=21,	-18<=k<=15,	-11 <= k< = 11,
	-24<=l<=24	-46<=l<=47	-24 <= l< = 25
Reflections collected	101047	94311	37218
Independent reflections	6138 [R(int) = 0.1237]	14095 [R(int) = 0.0306]	6282 [Rint = 0.1004]
Data/Restraints/Parameter	6138 / 0 / 352	14095 / 1184 / 910	6282 / 0 / 352
Goodness-of-fit on F^2	1.110	1.092	2.610
Final R indices [I>2sigma(I)]	R1 = 0.0719, wR2 = 0.2038	R1 = 0.0733, wR2 = 0.1724	R1 = 0.1445, wR2 = 0.4954
Largest diff. peak and hole [e·Å-3]	0.610 and -1.512	1.466 and -0.687	8.008 and -2.357

4.2. Molecular structure of 4

Molecular structure of 4a^{PO}

All hydrogen atoms were placed on ideal positions.

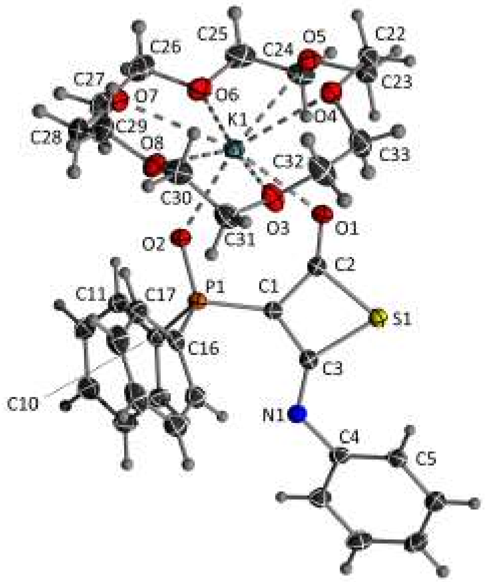

Figure S71 Molecular structure of compound **4a^{PO}**. Thermal ellipsoids at 50% probability level. Selected bond lengths [Å] and angles [°]: P1–C1 1.766(1), C1–C2 1.414(2), C1–C3 1.434(2), C2–S1 1.891(1), C3–S1 1.834(1), C3–N1 1.274(2), C2–O1 1.217(2), C2–C1–C3 99.0(1), C2–S1–C3 71.1(1), C1–C3–S1 95.8(1), C1–C2–S1 94.0(1).

Molecular structure of 4^{Ts}

All hydrogen atoms were placed on ideal positions. The crystal structure contained a disordered 12-crown-4 ligand, which was refined using the PART instructions and free variables, leading to occupancies of 56% and 44%.

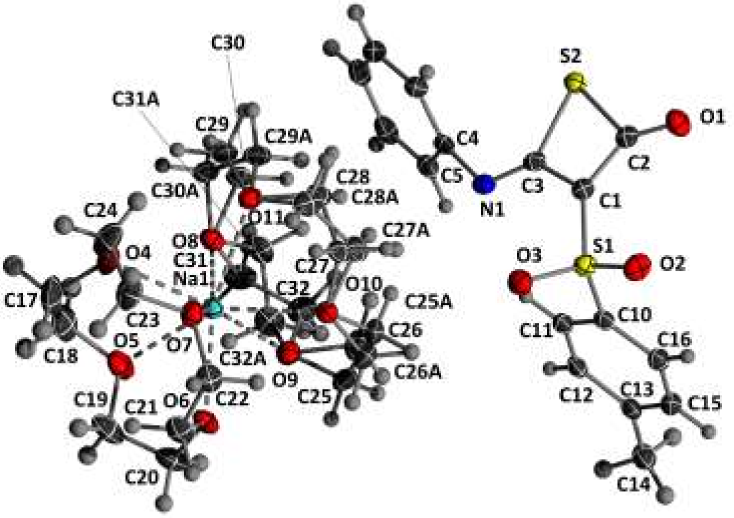

Figure S72 Molecular structure of compound **4^{Ts}**. Thermal ellipsoids at 50% probability level. Selected bond lengths [Å] and angles [°]: S1-C1 1.719(1), C1-C2 1.413(2), C1-C3 1.435(2), C2-S2 1.921(1), C3-S2 1.827(1), C3-N1 1.270(2), C2-O3 1.204(2), C2-C1-C3 101.0(1), C2-S2-C3 71.8(1), C1-C3-S2 95.2(1), C1-C2-S2 92.0(1).

4.3. Molecular structure of 3

Molecular structure of 3^{PS}

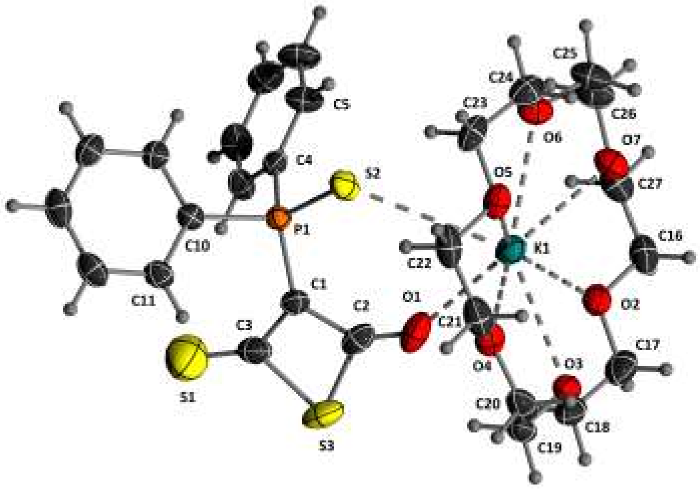

Figure S73 Molecular structure of compound **3^{PS}**. Thermal ellipsoids at 50% probability. The bond lengths and bond angles are not discussed due to the low quality of the crystal.

Molecular structure of 3^{Ts}

All hydrogen atoms were placed on ideal positions. The crystal structure contained a disordered 12-crown-4 ligand. The disorder was modelled using SAME, SIMU and DELU restraints and refined using the PART instructions and free variables, which optimized to occupancies of 81% and 19%. A second disorder of a four-member ring was modelled using the SIMU; DELU and SADI restraints and refined using the PART instructions and free variables, which optimized to occupancies of 64% and 36%. A third disorder in DCM was modelled using the EADP constraint and SADI restraint and refined using the PART instructions and free variables resulting in occupancies of 89% and 11%.

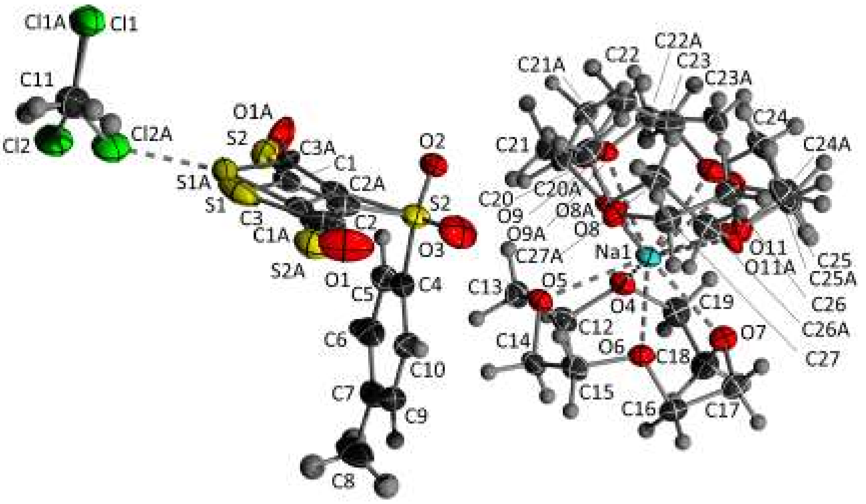

Figure S74 Molecular structure of compound **3^{Ts}**. Thermal ellipsoids at 50% probability level. Selected bond lengths [Å] and angles [°]: S3–C4 1.758(3), S3–C2 1.725(6), S3–C2A 1.794(9), C3–C2 1.407(8), C3A–C2A 1.421(1), C3–S1 1.899(9), C3A–S1A 1.900(1), C1–S1 1.816(5), C1A–S1A 1.821(1), C2–C1 1.411(8), C1A–C2A 1.418(1), C3–S1–C1 72.1(3), C3A–S1A–C1A 71.9(5), C3–C2–C1 101.9(6), C3A–C2A–C1A 100.7(9).

4.4. Molecular structure of 2

Molecular structure of 2^{PO}

All hydrogen atoms were placed on ideal positions. The crystal structure contained a THF molecule in a symmetry centre, which was refined using the PART -1 instructions and free variables, leading to occupancy of 50%.

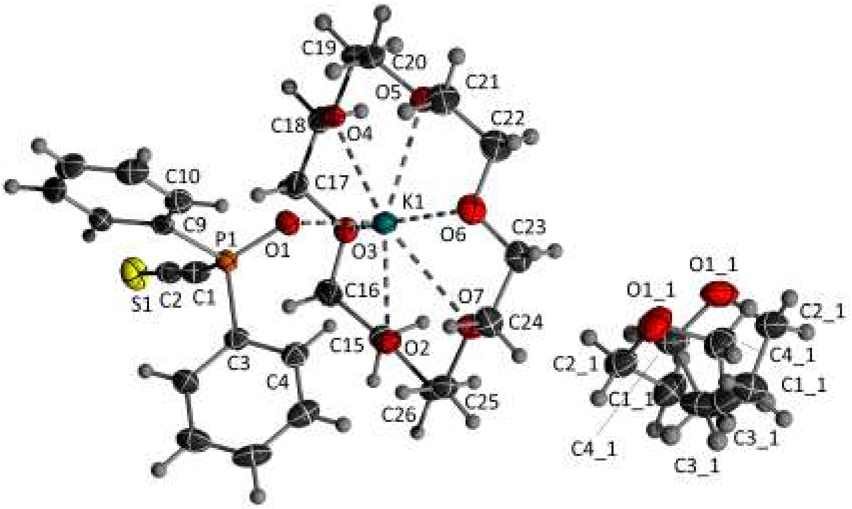

Figure S75 Molecular structure of compound $\mathbf{2^{PO}}$. Thermal ellipsoids at 50% probability level. Selected bond lengths [Å] and angles [°]: P1–O1 1.511(1), P1–C1 1.711(1), C1–C2 1.223(2), C2–S1 1.631(1), P1–C1–C2 170.0(1), C1–C2–S2 178.6(1).

Molecular structure of 2^{PS}

All hydrogen atoms were placed on ideal positions.

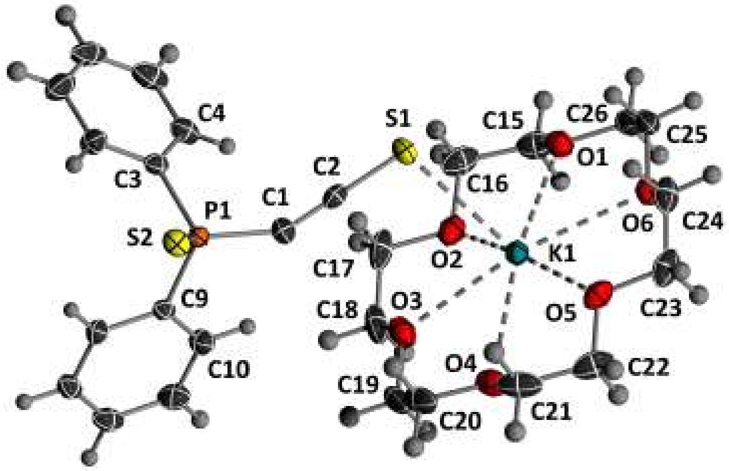

Figure S76 Molecular structure of compound $\mathbf{2^{PS}}$. Thermal ellipsoids at 50% probability level. Selected bond lengths [Å] and angles [°]: P1–S2 1.969(1), P1–C1 1.709(1), C1–C2 1.226(2), C2–S1 1.626(1), P1–C1–C2 150.9(1), C1–C2–S2 178.3(1).

Molecular structure of 2^{Ts}

All hydrogen atoms were placed on ideal positions. The structure has been solved as a two component twin using the MERG and BASF instructions refining to a ratio of 43% and 57% (twin law: 1.0 0.0 0.623 0.0 –1.0 0.0 0.0 0.0 –1.0)

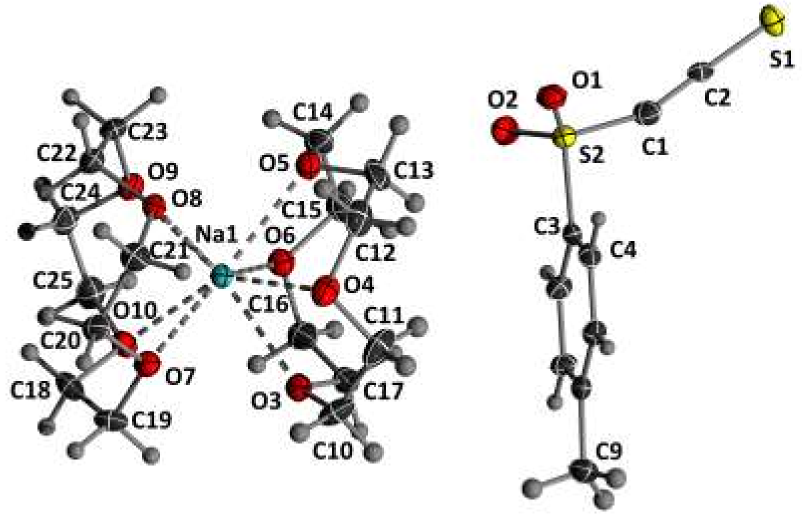

Figure S77 Molecular structure of compound 2^{Ts}. Thermal ellipsoids at 50% probability level. Selected bond lengths [Å] and angles [°]: S2–C3 1.780(6), S2–C1 1.661(6), C1–C2 1.228(9), C2–S1 1.618(6), S2–C1–C2 157.9(6), C1–C2–S2 178.0(5), C3–S2–C1 104.9(3).

4.5. Molecular structure of 8

All hydrogen atoms were placed on ideal positions.

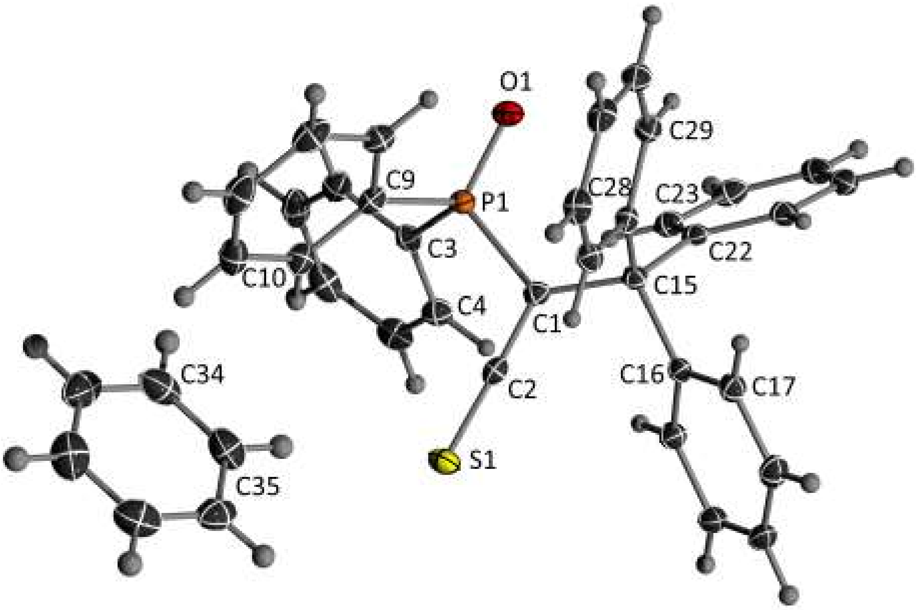

Figure S78 Molecular structure of compound **8**. Thermal ellipsoids at 50% probability level. Selected bond lengths [Å] and angles [°]: P1–O1 1.483(1), P1–C1 1.822(1), C1–C2 1.316(2), C2–S1 1.552(1), C1–C15 1.554(2), P1–C1–C2 114.1(1), C1–C2–S2 176.2(1).

4.6. Molecular structure of 9

All hydrogen atoms were placed on ideal positions.

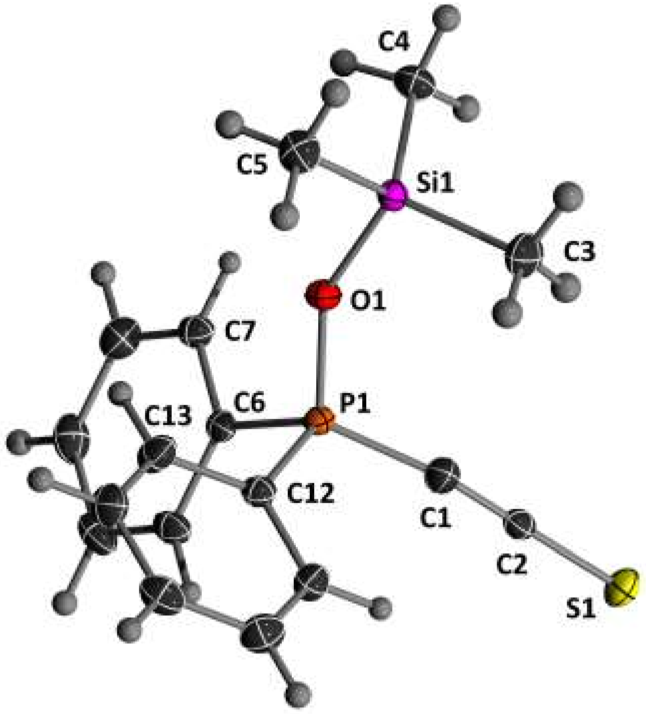

Figure S79 Molecular structure of compound **9**. Thermal ellipsoids at 50% probability level. Selected bond lengths [Å] and angles [°]: P1-O1 1.556(1), P1-C1 1.669(2), C1-C2 1.232(2), C2-S1 1.609(1), O1-Si1 1.690(1), P1-C1-C2 165.0(2), C1-C2-S2 179.0(1).

4.7. Molecular structure of 10a

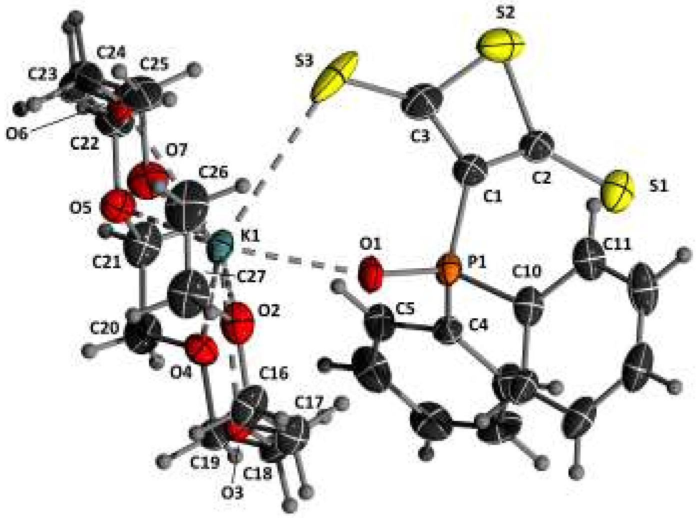

Figure S80 Molecular structure of compound **10a**. Thermal ellipsoids at 50% probability. The bond lengths and bond angles are not discussed due to the low quality of the crystal.

4.8. Molecular structure of 11

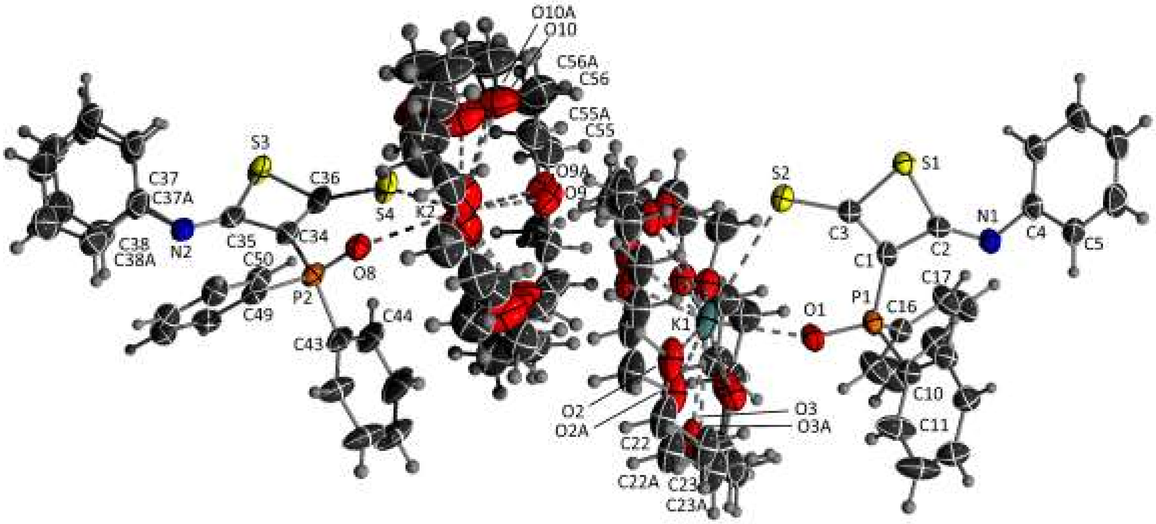

Figure S81 Molecular structure of compound **11**. Thermal ellipsoids at 50% probability. The bond lengths and bond angles are not discussed due to the low quality of the crystal.

4.9. Molecular structure of 12

All hydrogen atoms were placed on ideal positions. The crystal structure contained a disordered pentane, which was squeezed.

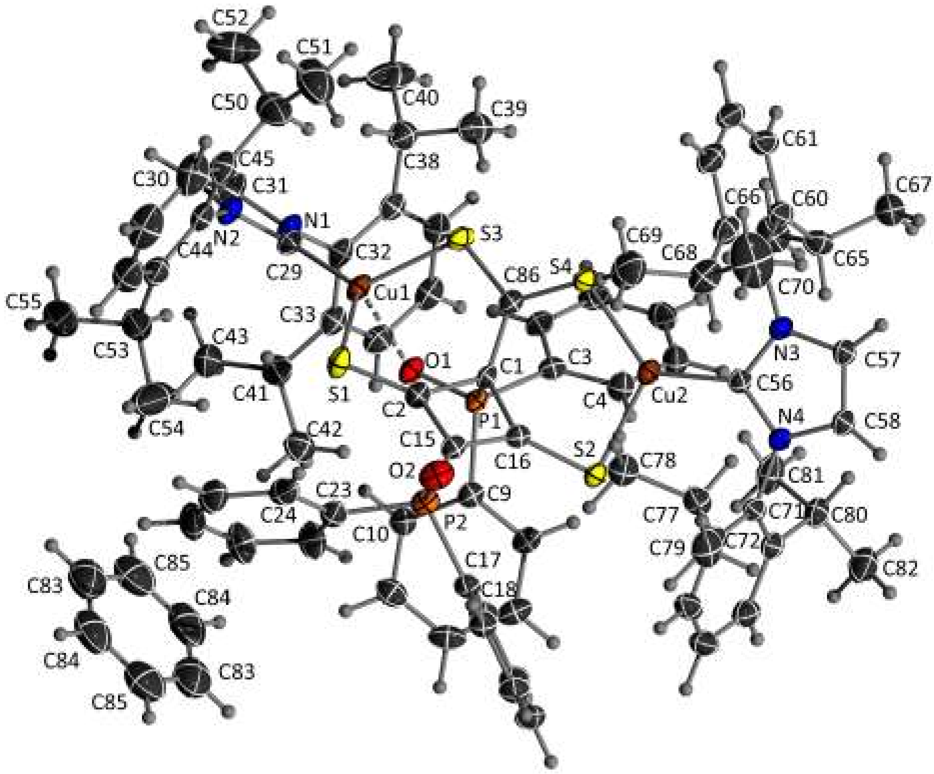

Figure S82 Molecular structure of compound **12**. Thermal ellipsoids at 50% probability level. Selected bond lengths [Å] and angles [°]: C1–C2 1.572(2), C2–C15 1.428(2), C15–C16 1.405(3), C16–C1 1.557(2), C1–C86 1.516(2), Cu1–C29 1.923(2), Cu2–C56 1.932(2), P1–C1–C2 109.3(1), P1–C15–C16 109.3(1).

5. DFT Calculations

5.1. Computational details

All calculations were carried out with the Amsterdam Density Functional program (ADF2024.1),[8–10] as implemented in the Amsterdam Modeling Suite (AMS2024.1) software package.[11,12] Geometries and energies were obtained with the BP86 level of the generalized gradient approximation (GGA).[13–15] Scalar relativistic effects were accounted for by using the zeroth-order regular approximation (ZORA)[16–18]. Molecular orbitals (MOs) were expanded in a large, uncontracted set of Slater-type orbitals (STOs), specifically the TZ2P basis set, which is of triple-ζ quality and is augmented with two sets of polarization functions.[19] All electrons were treated variationally. The numerical accuracy was set to VERYGOOD.[20,21] Voronoi deformation density (VDD)[22] charges and Mayer bond orders (MBO)[23,24] have also been computed at the same level of theory. All stationary points were verified to be minima (no imaginary frequencies) or transition states (only one imaginary frequency) on the potential energy surface through vibrational analysis.[25–27] Furthermore, the normal mode character of the imaginary frequency was analysed to ensure that it is associated with the reaction of interest.[28–30] Molecular structures were illustrated using CYLview.[31]

5.2. Activation strain model and energy decomposition analysis

To analyze the bonding situation between $[P(O)Me_2C]^-$ in its singlet valence configuration (Figure 8a) and L2 (Figure S83), the electronic bonding energy ΔE is divided into two major components using the activation strain model (ASM):[32–37]

$$\Delta E = \Delta E_{strain} + \Delta E_{int} \tag{S1}$$

Here, the strain energy (ΔE_{strain}) results from the distortion of the fragments $[P(O)Me_2C]^-$ and L2 from their equilibrium structure to the geometry they acquire in the final $[P(O)Me_2C\text{–}L2]^-$ molecule. The interaction energy (ΔE_{int}) accounts for all chemical interactions between the geometrically deformed fragments in the final molecule.

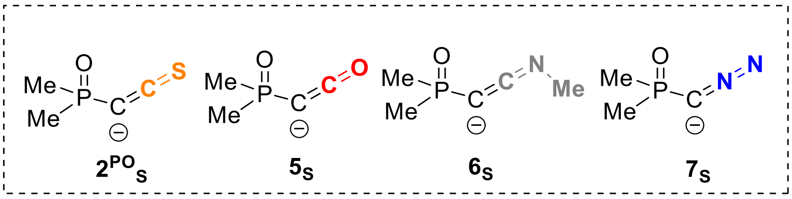

Figure S83 Simplified model series of thioketenyl anion and related compounds used in the bonding analysis.

The interaction energy ΔE_{int} is further analyzed within the framework of the quantitative Kohn-Sham molecular orbital (KS-MO) theory by partitioning it using our canonical energy decomposition analysis (EDA)[38–40] scheme into three physically meaningful energy terms, namely, electrostatic interaction (ΔV_{elstat}), Pauli repulsion (ΔE_{Pauli}), and (stabilizing) orbital interactions (ΔE_{oi}):

$$\Delta E_{int} = \Delta V_{elstat} + \Delta E_{Pauli} + \Delta E_{oi} \tag{S2}$$

The electrostatic energy ΔV_{elstat} corresponds to the electrostatic interactions between the unperturbed charge distribution of the distorted fragments, which is usually attractive. The Pauli repulsion ΔE_{Pauli} comprises the destabilizing interactions between occupied orbitals (or, more precisely, same-spin electrons on either fragment) and is responsible for any steric repulsion. The orbital interactions ΔE_{oi} term accounts for the stabilizing orbital interactions between the fragments, which include charge transfer (donor–acceptor interaction between an occupied orbital of one fragment with an empty orbital of the other fragment) and polarization (empty/occupied orbital mixing on one fragment due to the presence of another fragment).

The orbital interactions ΔE_{oi} can be further decomposed into the contributions from each irreducible representation (irrep) Γ of the point group of the corresponding system. In the case of our C_s symmetric $[P(O)Me_2C]^-$ fragments interacting with the L2 ligand, it can be divided into the in-plane (irreps A′) and out-of-plane (irrep A″) contributions.

$$\Delta E_{oi} = \Delta E_{oi,A'} + \Delta E_{oi,A''}$$

The open-source PyFrag19 program was used to automate the analysis of the bonding mechanism as a function of the P–C–L2 bond angle (where L2 = CS, CO, CNMe, N_2).[41–43]

5.3. Results from structure optimization

Table S5 Calculated and experimental bond lengths (in Å) and angles (in degree), electronic bonding energy ΔE, and bond dissociation energies D_e (in kcal mol^{-1}) of the anions **2**^{Ts}, **2**^{PO}, **1**^{PO}, **5** and **6**.

System	Structures		Calc.[a]	Exper.[b]
**2**^{Ts}	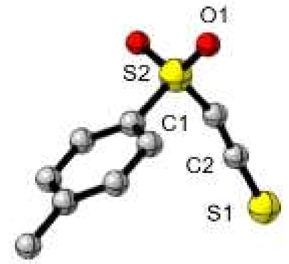	S2–O1	1.470	–
		S2–C1	1.649	1.661(6)
		C1–C2	1.253	1.228(9)
		C2–S1	1.619	1.618(6)
		S2–C1–C2	161.7	157.9(6)
		C1–C2–S1	177.7	178.0(5)
**2**^{PO}	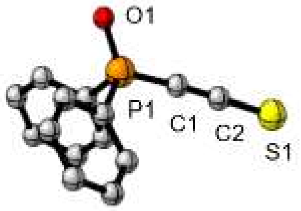	P1–O1	1.508	1.511(9)
		P1–C1	1.692	1.711(1)
		C1–C2	1.253	1.223(2)
		C2–S1	1.625	1.631(1)
		P1–C1–C2	176.9	170.0(1)
		C1–C2–S1	179.5	178.6(1)
		ΔE[c]	−146.9	–
		D_e[c]	143.3	–
**1**^{PO}	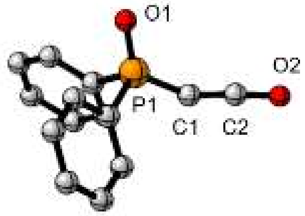	P1–O1	1.512	1.496(1)
		P1–C1	1.684	1.686(2)
		C1–C2	1.269	1.240(2)
		C2–O2	1.206	1.212(2)
		P1–C1–C2	151.3	148.9(1)
		C1–C2–O2	175.9	175.1(2)
		ΔE[c]	−106.2	–
		D_e[c]	102.8	–

**5**	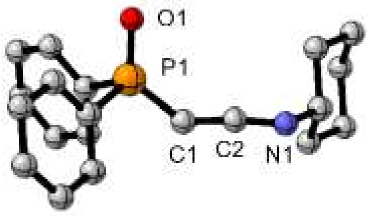	P1–O1	1.514	1.497(3)
		P1–C1	1.703	1.696(5)
		C1–C2	1.279	1.283(6)
		C2–N1	1.258	1.246(6)
		P1–C1–C2	138.9	139.3(4)
		C1–C2–N1	173.5	172.6(5)
		ΔE[c]	−100.5	–
		D_e[c]	98.5	–
**6**	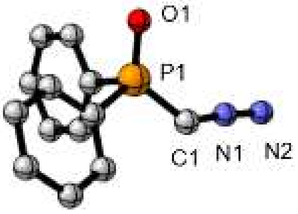	P1–O1	1.513	1.492(2)
		P1–C1	1.715	1.710(3)
		C1–N1	1.268	1.254(3)
		N1–N2	1.167	1.163(3)
		P1–C1–N1	126.4	125.9(2)
		C1–N1–N2	173.6	170.3(2)
		ΔE[c]	−54.7	–
		D_e[c]	51.2	–

[a] Computed at ZORA-BP86/TZ2P level of theory for the naked anions, that is, in the absence of the cationic counterion. [b] Experimental values are taken from the X–ray structure analysis of the complexes. [c] Energies calculated relative to the fragments $[Ph_2OPC]^-$ + L2 in their electronic ground states.

Table S6 Calculated frontier orbitals with associated energy level ε (in eV) of **2^{PO}** and **2^{Ts}** at ZORA–BP86/TZ2P level of theory

Species	**2^{PO}($POPh_2CCS$)**	**2^{Ts}($tolSO_2CCS$)**
LUMO	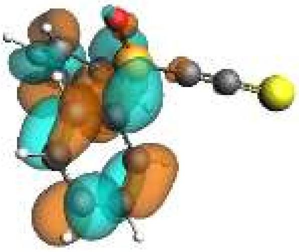 (ε= 0.88 eV)	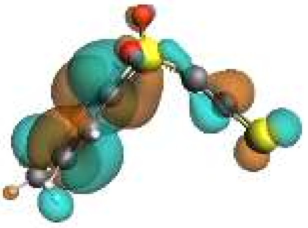 (ε= 1.11 eV)
HOMO	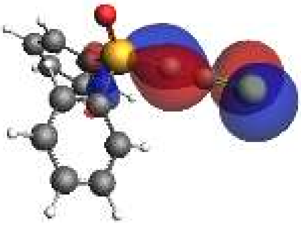 (ε= −1.15 eV)	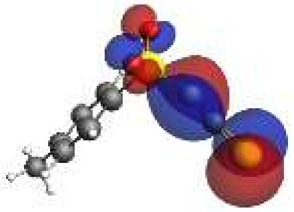 (ε= −1.11 eV)
HOMO−1	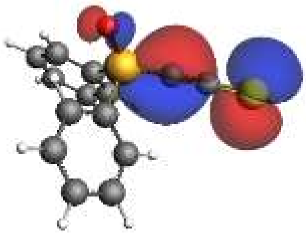 (ε= −1.21 eV)	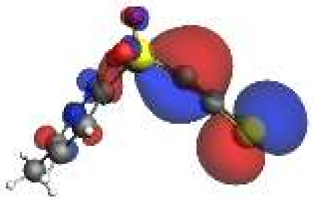 (ε= −1.36 eV)
HOMO–LUMO GAP	2.03 eV	2.22eV

Table S7 Calculated proton affinities PAs (in eV) and frontier orbitals with associated energy levels ε (in eV) of two isomers (Thioketene and Alkynyl sulfide) of protonated **2^{PO}** (**2^{PO}–H$_C$** and **2^{PO}–H$_S$**) and protonated **2^{Ts}** (**2^{Ts}–H$_C$** and **2^{Ts}–H$_S$**) at ZORA–BP86/TZ2P level of theory.

Isomer form	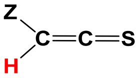 **Thioketene**		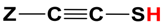 **Alkynyl Sulfide**	
Species	**2^{PO}–H$_C$**	**2^{Ts}–H$_C$**	**2^{PO}–H$_S$**	**2^{Ts}–H$_S$**
PAs	14.0	14.1	13.2	13.1
LUMO	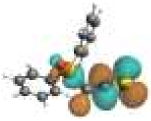 (ε= −3.40 eV)	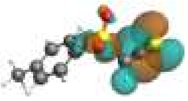 (ε= −3.89 eV)	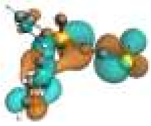 (ε= −2.29 eV)	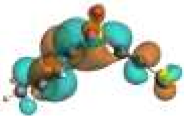 (ε= −2.46 eV)
HOMO	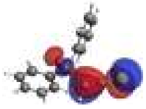 (ε= −5.90 eV)	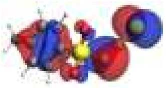 (ε= −6.13 eV)	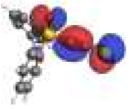 (ε= −6.16 eV)	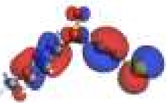 (ε= −6.34 eV)
HOMO-LUMO GAP	2.50 eV	2.24 eV	3.87 eV	3.88 eV

Table S8 Calculated frontier orbitals with associated energy levels ε (in eV) of **2^{PO}**, **1^{PO}**, **5** and **6** at ZORA-BP86/TZ2P level of theory.

Species	**POPh$_2$CCS**	**POPh$_2$CCO**	**POPh$_2$CCNCy**	**POPh$_2$CNN**
LUMO	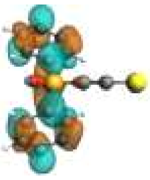 (ε= 0.88 eV)	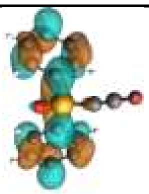 (ε= 1.15 eV)	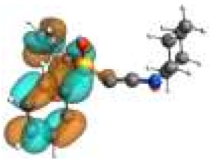 (ε= 1.13 eV)	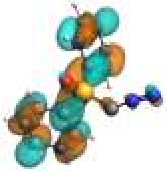 (ε= 1.18 eV)
HOMO	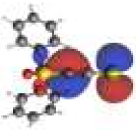 (ε= −1.15 eV)	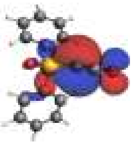 (ε= −1.16 eV)	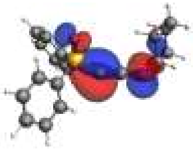 (ε= −0.83 eV)	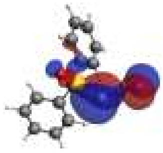 (ε= −0.74 eV)
HOMO–LUMO GAP	2.03 eV	2.31 eV	1.96 eV	1.92 eV

Table S9 Calculated bond lengths [Å] and angles [degree] of the anions **2**$^{PO}_{S}$, **1**$^{PO}_{S}$, **5**$_{S}$ and **6**$_{S}$. at ZORA-BP86/TZ2P level of theory. Calculated electronic binding energy ΔE [kcal mol^{-1}] and bond dissociation energies D_e [kcal mol^{-1}] to the fragments [$Me_2P(O)C$]$^-$ + L2 in the electronic ground states.

		**Bond/ angle**	**Calc.**
**2**$^{PO}_{S}$	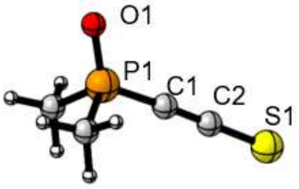	P1–O1	1.509
		P1–C1	1.701
		C1–C2	1.251
		C2–S1	1.632
		P1–C1–C2	177.5
		C1–C2–S1	179.8
	ΔE (D_e)	−150.7(147.1)	
**1**$^{PO}_{S}$	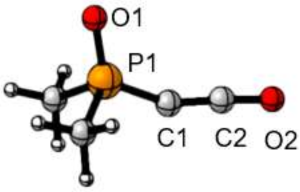	P1–O1	1.514
		P1–C1	1.692
		C1–C2	1.266
		C2–O2	1.212
		P1–C1–C2	153.1
		C1–C2–O2	176.0
	ΔE (D_e)	−108.0(105.2)	
**5**$_{S}$	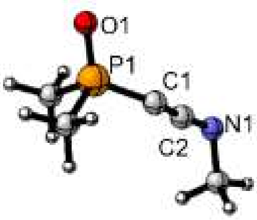	P1–O1	1.512
		P1–C1	1.712
		C1–C2	1.275
		C2–N1	1.266
		P1–C1–C2	139.4
		C1–C2–N1	174.0
	ΔE (D_e)	−101.8(99.6)	
**6**$_{S}$	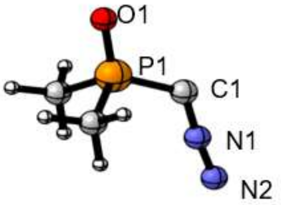	P1–O1	1.508
		P1–C1	1.726
		C1–N1	1.265
		N1–N2	1.175
		P1–C1–N1	123.2
		C1–N1–N2	174.8
	ΔE (D_e)	−55.6(52.7)	

Table S10 Calculated Voronoi deformation density (VDD) atomic charges (Q) of the anions $[C(Z)(L2)]^{-}$: **2**^{PO} (Z = $P(O)Ph_2$; L2 = CS), **2**$^{PO}_{S}$ (Z = $P(O)Me_2$; L2 = CS), **1**^{PO} (Z = $P(O)Ph_2$; L2 = CO), **1**$^{PO}_{S}$ (Z = $P(O)Me_2$; L2 = CO), **5** (Z = $P(O)Ph_2$; L2 = CNCy), **5**$_{S}$ (Z = $P(O)Me_2$; L2 = CNMe), **6** (Z = $P(O)Ph_2$; L2 = N2) **6**$_{S}$ (Z = $P(O)Me_2$; L2 = N2) and **2**^{Ts} (Z = SO_2tol; L2 = CS) at the ZORA–BP86/TZ2P level of theory.

Species	Charges Q		Species	Charges Q	
**2**^{PO}	C1	−0.30	**2**$^{PO}_{S}$	C1	−0.31
	$POPh_2$	−0.27		$POMe_2$	−0.21
	CS	−0.43		CS	−0.48
**1**^{PO}	C1	−0.39	**1**$^{PO}_{S}$	C1	−0.42
	$POPh_2$	−0.38		$POMe_2$	−0.30
	CO	−0.23		CO	−0.28
**5**	C1	−0.37	**5**$_{S}$	C1	−0.40
	$POPh_2$	−0.39		$POMe_2$	−0.28
	CNCy	−0.24		CNMe	−0.32
**6**	C1	−0.37	**6**$_{S}$	C1	−0.41
	$POPh_2$	−0.41		$POMe_2$	−0.28
	N_2	−0.22		N_2	−0.31
**2**^{Ts}	C1	−0.26			
	SO_2tol	−0.35			
	CS	−0.39			

5.4. Results from Bonding Analysis

Table S11 Activation strain model (ASM) and energy decomposition analysis (EDA) terms (in kcal mol^{-1}) in **2**$^{PO}_{S}$, **1**$^{PO}_{S}$, **5**$_{S}$, and **6**$_{S}$ using $[(POMe_2)C]^{-}$ + L2 as interacting fragments in their electronic singlet valence states and equilibrium geometries at the ZORA-BP86/TZ2P level of theory.

[P]	L2	ΔE	ΔE_{strain}	ΔE_{int}	ΔV_{elstat}	ΔE_{Pauli}	ΔE_{oi}
$P(O)Me_2C^{-}$	N2	−55.6	125.9	−181.5	−312.4	722.4	−591.5
$P(O)Me_2C^{-}$	CO	−108.0	122.6	−230.7	−223.8	473.7	−480.5
$P(O)Me_2C^{-}$	CNMe	−100.8	138.4	−239.1	−194.0	411.5	−456.7
$P(O)Me_2C^{-}$	CS	−150.7	121.5	−272.2	−194.5	421.7	−499.4

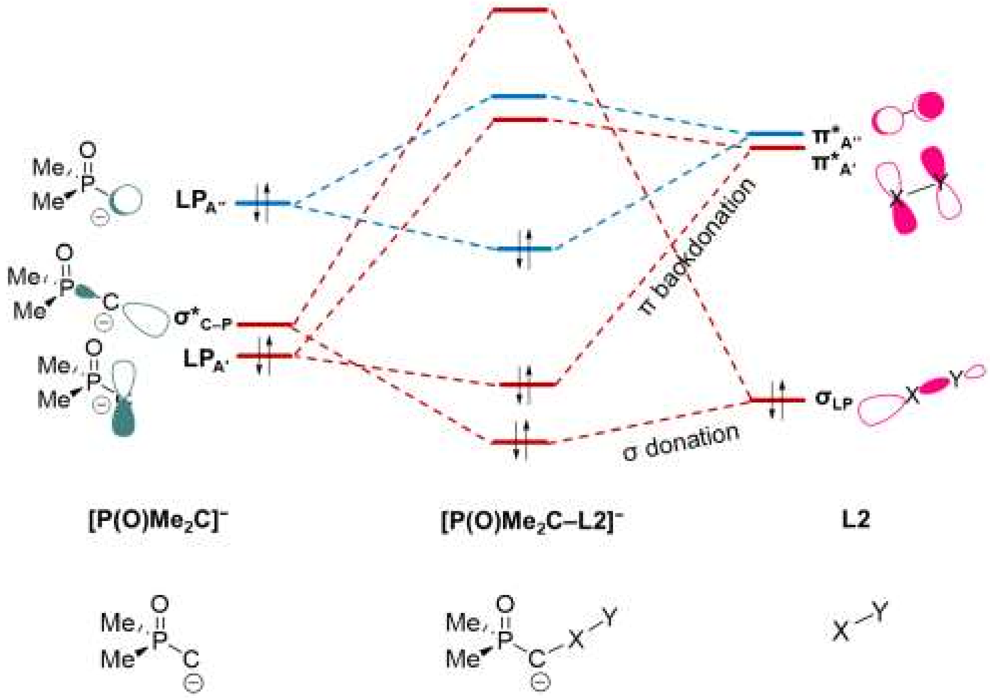

Figure S84 Schematic molecular orbital diagram for the σ-donation and π-backdonation interactions of the $[P(O)Me_2C]^-$ fragment with L2 (L2= CS, CO, CNMe, N_2).

Table S12. Activation strain model (ASM) and energy decomposition analysis (EDA) terms (in kcal mol^{-1}) in **2^{PO}$_S$**, **5$_S$**, **6$_S$**, and **7$_S$** using $[(POMe2)C]^-$ + L2 as interacting fragments in their electronic singlet valence states and consistent geometry with P–C–L2 = 120° at the ZORA-BP86/TZ2P level of theory.[a]

[P]	L	ΔE	ΔE_{strain}	ΔE_{int}	ΔV_{elstat}	ΔE_{Pauli}	ΔE_{oi}
P(O)Me2C$^-$	N_2	−55.6	125.1	−180.7	−311.8	720.9	−589.8
$P(O)Me_2C^-$	CO	−105.5	122.9	−228.5	−327.6	717.2	−618.1
$P(O)Me_2C^-$	CNMe	−93.7	135.0	−228.7	−327.4	717.6	−618.9
$P(O)Me_2C^-$	CS	−144.1	122.2	−266.3	−344.7	739.3	−660.9

[a] using C_S symmetry.

Table S13 Orbital interaction (ΔE_{oi}) terms of the A′ and A″ irreducible representations of C_S symmetry in the anionic systems $[P(O)Me_2C–L2]^-$ (where L2 = CS, CO, CNMe, N_2) in a consistent geometry with P–C–L2 = 120° at the ZORA-BP86/TZ2P level of theory.[a]

[P]	L	$\Delta E_{oi,A'}$	$\Delta E_{oi,A''}$
$P(O)Me_2C^-$	N_2	−502.5	−87.4
$P(O)Me_2C^-$	CO	−526.5	−91.5
$P(O)Me_2C^-$	CNMe	−538.6	−80.3
$P(O)Me_2C^-$	CS	−549.0	−112.0

[a] using C_S symmetry.

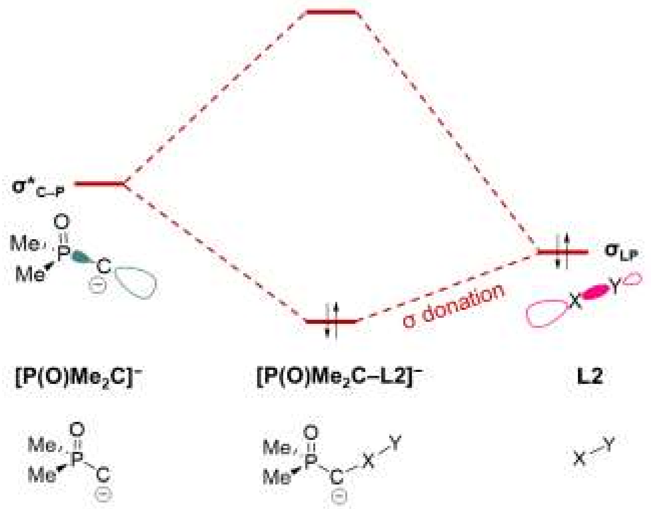

L2	ε[σLP]	Gp[σ*]	Gp[σLP]	S
N2	−10.2	1.409	1.404	0.23
CO	−9.3	1.428	1.104	0.35
CNMe	−7.5	1.401	1.233	0.27
CS	−7.7	1.363	1.034	0.31

Figure S85 Schematic representation of the σ-donation (lone-pair orbital on the L2 donates electrons into the empty $\sigma^*_{C\text{-}P}$ orbital of the $[P(O)Me_2C]^-$ fragment) in the A′ irreducible representation of the C_S symmetry at a consistent geometry with P–C–L2 = 120°. ε = Orbitals energy in eV; Gp = Gross Mulliken population; and S = Orbital overlap. All values computed at the ZORA-BP86/TZ2P level of theory.

a)

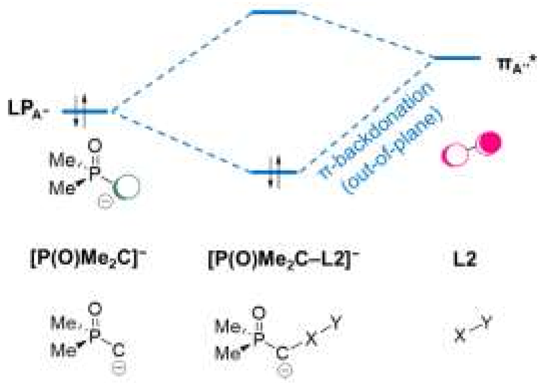

L2	ε[πA″*]	Gp[LPA″]	Gp[πA″*]	S
N2	−2.7	1.326	0.643	0.26
CO	−2.7	1.337	0.639	0.37
CNMe	−1.5	1.396	0.584	0.36
CS	−3.7	1.160	0.804	0.32

b)

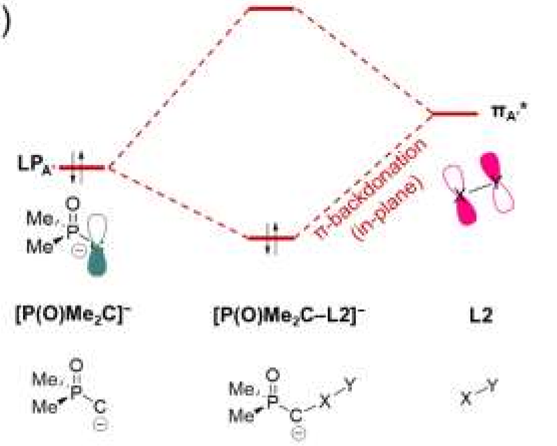

L2	ε[πA′*]	Gp[LPA′]	Gp[πA′*]	S
N2	−2.7	1.007	0.431	0.12
CO	−2.7	1.135	0.465	0.18
CNMe	−2.8	1.087	0.556	0.15
CS	−3.7	1.107	0.569	0.16

Figure S86 Schematic representation of the (a) out-of-plane and (b) in-plane π-backdonation (lone-pair orbitals of $[P(O)Me_2C]^-$ fragment donate electrons into the empty π*-orbitals of L2) at a consistent geometry with P–C–L2 = 120°. ε = Orbitals energy in eV; Gp = Gross Mulliken population; and S = Orbital overlap. All values computed at the ZORA-BP86/TZ2P level of theory.

5.5. Calculated Mechanism for the formation of 2

Formation of 2^{PO} via pathway 2

We probed the mechanism for the formation of $\mathbf{2^{PO}}$ upon reaction of phenyl isothiocyanate with ketenyl anion $\mathbf{1^{PO}}$. The first step involves the formation of a four-membered thietane ring (**4**) via a 2+2 cycloaddition reaction with an energy barrier of 25.8 kcal mol^{-1}. This step is exergonic, with an energy change of −4.5 kcal mol^{-1}. From this intermediate, two possible pathways emerge. **4** may first rearrange into a four-membered azetidine ring ($\mathbf{INT3^{A}}$) and subsequently undergo a stepwise 2+2 cycloreversion reaction to release PhNCO and the thioketenyl anion $\mathbf{2^{PO}}$ (PathA in Figure S87). Alternatively, it can directly undergo a 2+2 cycloreversion to release OCS and the keteniminyl anion (PathB in Figure S87).

Both pathways were examined, and their Gibbs free energy profiles are compared in Figure S88. The conversion of **4** to $\mathbf{P^{A}}$ (Thioketenyl anion + PhNCO) is calculated to be slightly endergonic (ΔG = +0.7 kcal mol^{-1}). It proceeds through multiple steps, including ring opening, rotation along the C–C bond, ring closing, and stepwise cycloreversion reaction. Contrastingly, the conversion of **4** to $\mathbf{P^{B}}$ (Keteniminyl anion + OCS) is calculated to be more endergonic, with an energy change of +5.9 kcal mol^{-1}. This justifies why no experimental evidence corresponding to the formation of keteniminyl anion (PathB) is found even under forcing conditions.

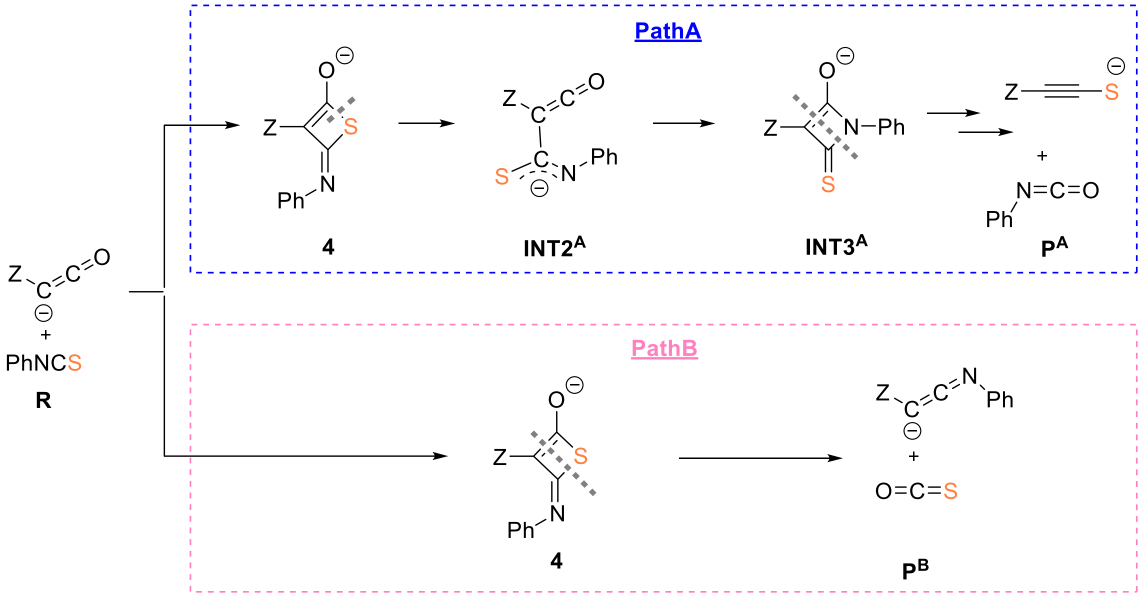

Figure S87 Possible pathways upon reaction of $\mathbf{2^{PO}}$ with PhNCS

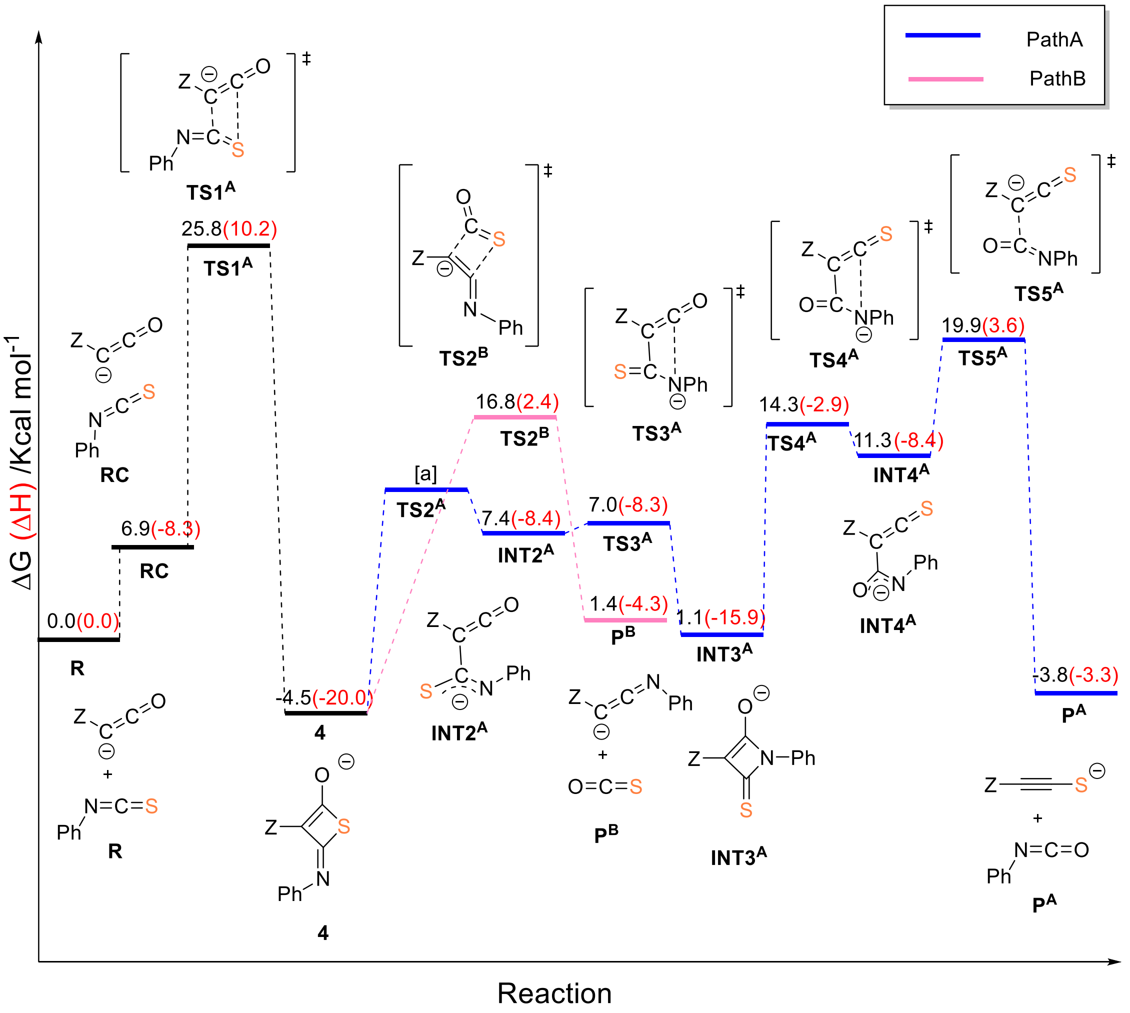

Figure S88 Energy profile for two possible pathways (Figure S87) upon reaction of Ketenyl anion **1**^{PO} with PhNCS at the ZORA-BP86/TZ2P level of theory. [a] Despite extensive computational searches, it was not possible to locate **TS2**^{A}.

6. References

[1] M. Jörges, S. Mondal, M. Kumar, P. Duari, F. Krischer, J. Löffler, V. H. Gessner, "Phosphinoyl-Substituted Ketenyl Anions: Synthesis and Substituent Effects on the Structural Properties" *Organometallics* **2024**, *43*, 585–593.

[2] M. Jörges, F. Krischer, V. H. Gessner, "Transition metal–free ketene formation from carbon monoxide through isolable ketenyl anions" *Science* **2022**, *378*, 1331–1336.

[3] F. Krischer, M. Jörges, T.-F. Leung, H. Darmandeh, V. H. Gessner, "Selectivity Control of the Ligand Exchange at Carbon in α-Metallated Ylides as a Route to Ketenyl Anions" *Angew. Chem. Int. Ed.* **2023**, *62*, e202309629.

[4] G. M. Sheldrick, "A short history of SHELX" *Acta Cryst A* **2008**, *64*, 112–122.

[5] G. M. Sheldrick, "Crystal structure refinement with SHELXL" *Acta Cryst C* **2015**, *71*, 3–8.

[6] G. M. Sheldrick, "SHELXT – Integrated space-group and crystal-structure determination" *Acta Cryst A* **2015**, *71*, 3–8.

[7] A. Thorn, B. Dittrich, G. M. Sheldrick, "Enhanced rigid-bond restraints" *Acta Cryst A* **2012**, *68*, 448–451.

[8] G. te Velde, F. M. Bickelhaupt, E. J. Baerends, C. Fonseca Guerra, S. J. A. van Gisbergen, J. G. Snijders, T. Ziegler, "Chemistry with ADF" *J. Comput. Chem.* **2001**, *22*, 931–967.

[9] C. Fonseca Guerra, J. G. Snijders, G. te Velde, E. J. Baerends, "Towards an order-N DFT method" *Theor. Chem. Acc.* **1998**, *99*, 391–403.

[10] ADF 2024.1; SCM: Theoretical Chemistry, Vrije Universiteit, Amsterdam (The Netherlands), 2024, http://www.scm.com.

[11] AMS 2024.1; SCM: Theoretical Chemistry, Vrije Universiteit, Amsterdam (The Netherlands), 2024, http://www.scm.com.

[12] E. J. Baerends, N. F. Aguirre, N. D. Austin, J. Autschbach, F. M. Bickelhaupt, R. Bulo, C. Cappelli, A. C. T. van Duin, F. Egidi, C. Fonseca Guerra, A. Förster, M. Franchini, T. P. M. Goumans, T. Heine, M. Hellström, C. R. Jacob, L. Jensen, M. Krykunov, E. van Lenthe, A. Michalak, M. M. Mitoraj, J. Neugebauer, V. P. Nicu, P. Philipsen, H. Ramanantoanina, R. Rüger, G. Schreckenbach, M. Stener, M. Swart, J. M. Thijssen, T. Trnka, L. Visscher, A. Yakovlev, S. van Gisbergen, "The Amsterdam Modeling Suite" *J. Chem. Phys.* **2025**, *162*, 162501.

[13] A. D. Becke, "Density-functional exchange-energy approximation with correct asymptotic behavior" *Phys. Rev. A* **1988**, *38*, 3098–3100

[14] J. P. Perdew, "Density-functional approximation for the correlation energy of the inhomogeneous electron gas" *Phys. Rev. B* **1986**, *33*, 8822–8824

[15] J. P. Perdew, "Erratum: Density-functional approximation for the correlation energy of the inhomogeneous electron gas" *Phys. Rev. B* **1986**, *34*, 7406–7406.

[16] E. van Lenthe, E. J. Baerends, J. G. Snijders, "Relativistic regular two-component Hamiltonians" *J. Chem. Phys.* **1993**, *99*, 4597–4610.

[17] E. van Lenthe, E. J. Baerends, J. G. Snijders, "Relativistic total energy using regular approximations" *J. Chem. Phys.* **1994**, *101*, 9783–9792.

[18] E. van Lenthe, A. Ehlers, E.-J. Baerends, "Geometry optimizations in the zero order regular approximation for relativistic effects" *J. Chem. Phys.* **1999**, *110*, 8943–8953.

[19] E. Van Lenthe, E. J. Baerends, "Optimized Slater-type basis sets for the elements 1–118" *J. Comput. Chem.* **2003**, *24*, 1142–1156.

[20] M. Franchini, P. H. T. Philipsen, L. Visscher, "The Becke Fuzzy Cells Integration Scheme in the Amsterdam Density Functional Program Suite" *J. Comput. Chem.* **2013**, *34*, 1819–1827.

[21] M. Franchini, P. H. T. Philipsen, E. van Lenthe, L. Visscher, "Accurate Coulomb Potentials for Periodic and Molecular Systems through Density Fitting" *J. Chem. Theory Comput.* **2014**, *10*, 1994–2004.

[22] C. Fonseca Guerra, J.-W. Handgraaf, E. J. Baerends, F. M. Bickelhaupt, "Voronoi deformation density (VDD) charges: Assessment of the Mulliken, Bader, Hirshfeld, Weinhold, and VDD methods for charge analysis" *J. Comput. Chem.* **2004**, *25*, 189–210.

[23] I. Mayer, "Charge, bond order and valence in the AB initio SCF theory" *Chem. Phys. Lett.* **1983**, *97*, 270–274.

[24] I. Mayer, "Bond order and valence: Relations to Mulliken's population analysis" *Int. J. Quantum Chem.* **1984**, *26*, 151–154.

[25] H. Jacobsen, A. Bérces, D. P. Swerhone, T. Ziegler, "Analytic second derivatives of molecular energies: a density functional implementation" *Comput. Phys. Commun.* **1997**, *100*, 263–276.

[26] A. Bérces, R. M. Dickson, L. Fan, H. Jacobsen, D. Swerhone, T. Ziegler, "An implementation of the coupled perturbed Kohn-Sham equations: perturbation due to nuclear displacements" *Comput. Phys. Commun.* **1997**, *100*, 247–262.

[27] S. K. Wolff, "Analytical second derivatives in the Amsterdam density functional package" *Int. J. Quantum Chem.* **2005**, *104*, 645–659.

[28] L. Deng, T. Ziegler, “The determination of intrinsic reaction coordinates by density functional theory” *Int. J. Quantum Chem.* **1994**, *52*, 731–765.
[29] L. Deng, T. Ziegler, L. Fan, “A combined density functional and intrinsic reaction coordinate study on the ground state energy surface of H_2CO” *J. Chem. Phys.* **1993**, *99*, 3823–3835
[30] K. Fukui, “The path of chemical reactions - the IRC approach” *Acc. Chem. Res.* **1981**, *14*, 363–368.
[31] C. Y. Legault, CYLview20; Université de Sherbrooke: Sherbrooke, Quebec, Canada, 2020; www.cylview.org.
[32] P. Vermeeren, S. C. C. van der Lubbe, C. Fonseca Guerra, F. M. Bickelhaupt, T. A. Hamlin, “Understanding chemical reactivity using the activation strain model” *Nat Protoc* **2020**, *15*, 649–667.
[33] F. M. Bickelhaupt, K. N. Houk, “Analyzing Reaction Rates with the Distortion/Interaction-Activation Strain Model” *Angew. Chem. Int. Ed.* **2017**, *56*, 10070–10086.
[34] I. Fernández, F. M. Bickelhaupt, “The activation strain model and molecular orbital theory: understanding and designing chemical reactions” *Chem. Soc. Rev.* **2014**, *43*, 4953–4967.
[35] L. P. Wolters, F. M. Bickelhaupt, “The activation strain model and molecular orbital theory” *Wiley Interdiscip. Rev.: Comput. Mol. Sci.* **2015**, *5*, 324–343.
[36] F. M. Bickelhaupt, “Understanding reactivity with Kohn–Sham molecular orbital theory: E2–SN2 mechanistic spectrum and other concepts” *J. Comput. Chem.* **1999**, *20*, 114–128.
[37] W.-J. van Zeist, F. M. Bickelhaupt, “The activation strain model of chemical reactivity” *Org. Biomol. Chem.* **2010**, *8*, 3118–3127.
[38] T. A. Hamlin, P. Vermeeren, C. F. Guerra, F. M. Bickelhaupt in *Complementary Bonding Analysis* (Ed.: S. Grabowsky), De Gruyter, **2021**, pp. 199–212.
[39] F. M. Bickelhaupt, E. J. Baerends in *Reviews in Computational Chemistry*, John Wiley & Sons, Ltd, **2000**, pp. 1–86.
[40] R. van Meer, O. V. Gritsenko, E. J. Baerends, “Physical Meaning of Virtual Kohn–Sham Orbitals and Orbital Energies: An Ideal Basis for the Description of Molecular Excitations” *J. Chem. Theory Comput.* **2014**, *10*, 4432–4441.
[41] W.-J. V. Zeist, C. F. Guerra, F. M. Bickelhaupt, “PyFrag—Streamlining your reaction path analysis” *J. Comput. Chem.* **2008**, *29*, 312–315.
[42] X. Sun, T. M. Soini, J. Poater, T. A. Hamlin, F. M. Bickelhaupt, “PyFrag 2019—Automating the exploration and analysis of reaction mechanisms” *J. Comput. Chem.* **2019**, *40*, 2227–2233.
[43] PyFrag 2007-2020: X. Sun, T. Soini, L. P. Wolters, W.-J. van Zeist, C. Fonseca Guerra, T. A. Hamlin, F. M. Bickelhaupt, Vrije Universiteit Amsterdam, The Netherlands.
